# H3.3-G34 mutations impair DNA repair and promote cGAS/STING-mediated immune responses in pediatric high-grade glioma models

**DOI:** 10.1172/JCI154229

**Published:** 2022-11-15

**Authors:** Santiago Haase, Kaushik Banerjee, Anzar A. Mujeeb, Carson S. Hartlage, Fernando M. Núñez, Felipe J. Núñez, Mahmoud S. Alghamri, Padma Kadiyala, Stephen Carney, Marcus N. Barissi, Ayman W. Taher, Emily K. Brumley, Sarah Thompson, Justin T. Dreyer, Caitlin T. Alindogan, Maria B. Garcia-Fabiani, Andrea Comba, Sriram Venneti, Visweswaran Ravikumar, Carl Koschmann, Ángel M. Carcaboso, Maria Vinci, Arvind Rao, Jennifer S. Yu, Pedro R. Lowenstein, Maria G. Castro

**Affiliations:** 1 Department of Neurosurgery,; 2 Department of Cell and Developmental Biology,; 3 Department of Pathology,; 4 Departments of Bioinformatics and Computational Biology, and; 5 Division of Pediatric Hematology/Oncology, Department of Pediatrics, C.S. Mott Children’s Hospital, University of Michigan, Ann Arbor, Michigan, USA.; 6 Institut de Recerca Sant Joan de Deu, Barcelona, Spain.; 7 Department of Onco-Haematology, Gene and Cell Therapy, Bambino Gesù Children’s Hospital-IRCCS, Rome, Italy.; 8 Radiation Oncology, University of Michigan, Ann Arbor, Michigan, USA.; 9 Department of Cancer Biology, Lerner Research Institute and; 10 Department of Radiation Oncology, Taussig Cancer Institute, Cleveland Clinic, Cleveland, Ohio, USA.

**Keywords:** Oncology, Therapeutics, Brain cancer, DNA repair, Drug therapy

## Abstract

Pediatric high-grade gliomas (pHGGs) are the leading cause of cancer-related deaths in children in the USA. Sixteen percent of hemispheric pediatric and young adult HGGs encode Gly34Arg/Val substitutions in the histone H3.3 (H3.3-G34R/V). The mechanisms by which H3.3-G34R/V drive malignancy and therapeutic resistance in pHGGs remain unknown. Using a syngeneic, genetically engineered mouse model (GEMM) and human pHGG cells encoding H3.3-G34R, we demonstrate that this mutation led to the downregulation of DNA repair pathways. This resulted in enhanced susceptibility to DNA damage and inhibition of the DNA damage response (DDR). We demonstrate that genetic instability resulting from improper DNA repair in G34R-mutant pHGG led to the accumulation of extrachromosomal DNA, which activated the cyclic GMP–AMP synthase/stimulator of IFN genes (cGAS/STING) pathway, inducing the release of immune-stimulatory cytokines. We treated H3.3-G34R pHGG–bearing mice with a combination of radiotherapy (RT) and DNA damage response inhibitors (DDRi) (i.e., the blood-brain barrier–permeable PARP inhibitor pamiparib and the cell-cycle checkpoint CHK1/2 inhibitor AZD7762), and these combinations resulted in long-term survival for approximately 50% of the mice. Moreover, the addition of a STING agonist (diABZl) enhanced the therapeutic efficacy of these treatments. Long-term survivors developed immunological memory, preventing pHGG growth upon rechallenge. These results demonstrate that DDRi and STING agonists in combination with RT induced immune-mediated therapeutic efficacy in G34-mutant pHGG.

## Graphical abstract

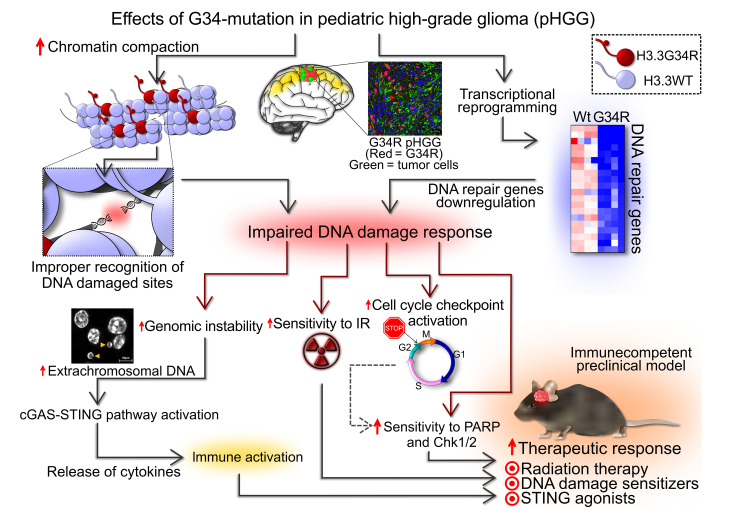

## Introduction

Pediatric high-grade gliomas (pHGGs) are aggressive brain tumors. With the current standard of care (SOC), patients with pHGG have a median overall survival of 9–15 months (1). Although the incidence of pHGG is low (1.78 per 100,000 people) (2), they are the primary cause of cancer-related deaths for patients under the age of 19 due to the lack of effective treatments (3).

In recent years, genetic and epigenetic hallmarks have been associated with various clinical features such as age of incidence, anatomical distribution, prognosis, and histological characteristics (4, 5). These findings revealed that the molecular alterations in pHGG differ from their adult counterparts. The understanding of the molecular hallmarks of pHGG allows the identification of specific therapeutic targets. Until now, pHGG therapies have targeted oncogenes such as PDGFRA originally identified in other cancers (6), rare mutational variants (e.g., BRAF V600E) (7, 8), or genetic mutations that are unique to midline diffuse gliomas (9).

Mutations in histones H3.3 and H3.1 are prevalent in pHGG (4, 10). The H3.3 lysine 27 to methionine (K27M) and H3.1-K27M mutations are found in midline high-grade gliomas (HGGs), whereas the H3.3-G34R/V mutations are present in cerebral hemispheric pHGG, correlating with different clinicopathological and biological subgroups (11). The molecular mechanisms mediated by these histone mutations are not well established, thus specific therapies for histone-mutated pHGG are not currently available.

Approximately 15% of hemispheric pHGGs encode G34R/V mutations on the H3.3 variant histone. H3.3-G34R/V-mutant pHGGs are located predominantly in the temporal and parietal lobes (2, 12, 13) and normally have *p53* and α-thalassemia/mental retardation, X-linked (*Atrx*) mutations (5).

H3.3-G34R expression was shown to alter H3 lysine 36 (K36) trimethylation (2, 14, 15). H3.3-G34R/V mutations were recently reported to affect K36me3 by inhibition of KDM4 demethylases (16), which leads to an abnormal accumulation of K36me3 and H3K9me3. Additionally, K36 can be acetylated, although the effects of G34 mutations on this mark have not been studied. H3.3-G34R/V can also affect the interaction of the histone tail with proteins that recognize K36 marks. For example, MutSα, a protein involved in mismatch repair (MMR) that normally recognizes K36me3 in replicating chromatin to prime MMR, was shown to be inhibited by G34 mutations (17). This was suggested to affect genomic instability and mutation rates (17). The clinical course of the H3.3-G34R/V subgroup is not completely clear. Some studies reported a moderate survival benefit over other hemispheric pHGGs (5, 13), while others have claimed that H3.3-G34R/V mutations correlate with a worse prognosis (18). Current SOC for non-midline pHGG, regardless of H3 status, involves maximal surgical resection followed by focal irradiation. For G34-mutant pHGG, the addition of chemotherapy beyond radiation remains controversial, in part because recent cooperative group trials preceded the increased testing for the G34 mutations. H3.3-G34R/V pHGGs have an increased frequency of *O*-6-methylguanine-DNA methyltransferase (*MGMT*) promoter methylation (18), which could lead to a better response to temozolomide (TMZ). Before the era of the molecular stratification, TMZ failed to improve outcomes in pHGG but was slightly more effective in children with low *MGMT* expression (Children’s Oncology Group [COG] study ACNS0126) (19). This would have included children with G34-mutant tumors, but given the lack of molecular stratification of the patients in this trial, it is unclear if that the patients in that group had better outcomes. A follow-up study (COG ACNS0423) revealed that lomustine (also called CCNU) added to TMZ increased 3-year event-free survival (EFS) rates from 11% to 22% in children with HGG, most significantly in children with low expression of *MGMT* (20, 21). These data overall support the use of TMZ and CCNU after radiation (COG ACNS0423) in patients with G34-mutant pHGG. However, the outcomes were poor with this protocol, thus, at most centers, patients with this type of pHGG consider participation in clinical trials. A study of patients with non-brainstem pHGG (COG ACNS1721) who were treated with the PARP inhibitor veliparib alongside TMZ ended early because of poor outcomes compared with historical controls (COG ACNS1721 study progress report; personal communication, Carl Koschmann, Pediatric Neuro-Oncology, University of Michigan, Ann Arbor, Michigan, USA). However, veliparib showed weak potency compared with other PARP inhibitors, and this clinical trial did not stratify for H3.3-G34R/V pHGG.

In this work, we demonstrate that H3.3-G34R/V expression leads to DNA repair deficiency in patients with pHGG, which translates into increased susceptibility to DNA damage from therapies such as ionizing radiation (IR). We show that this vulnerability could be further exploited by treating patients with H3.3-G34R pHGG with DNA damage response inhibitors (DDRi). We also demonstrate that impairment of DNA repair also induced genomic instability, leading to activation of the cyclic GMP–AMP synthase/stimulator of IFN genes (cGAS/STING) pathway and antitumor immunity in patients with H3.3-G34R pHGG.

## Results

### De novo–induced pHGG in genetically engineered mouse models and human models carrying the H3.3-G34R mutation, together with Atrx and p53 downregulation: phenotypic and molecular characterization.

**Figure 1:**
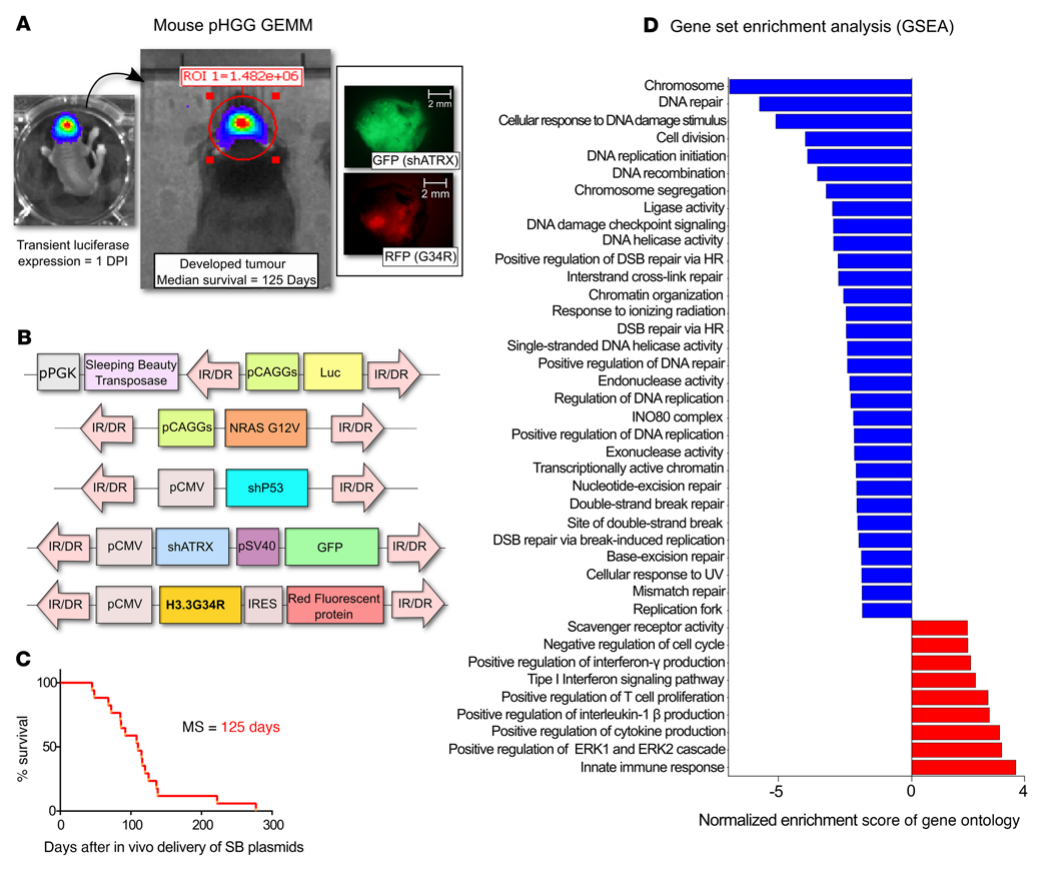
Characterization of a GEMM of G34R pHGG. (**A**) Procedure to induce genetically engineered H3.3-G34R pHGG in mice. Neonatal murine brain stem cells were transfected in vivo with SB transposase integration sequences to incorporate pHGG-inducing genetic lesions into the cells, including H3.3-G34R expression. pHGG development was monitored in vivo by luminescence driven by luciferase expression, and mice were perfused once signs of pHGG burden appeared; tumor tissue can be identified by its red (G34R) and green (ATRX-KO) fluorescence. Scale bars: 2 mm. DPI, days post implantation. (**B**) Illustration of the transposable fragments of the plasmids used to induce H3.3-G34R pHGG in mice via SB transposition. (**C**) Survival of animals transfected in vivo to develop de novo H3.3-G34R pHGG. (**D**) Selection of differentially enriched GOs between H3.3-G34R and H3.3-WT de novo–induced mouse pHGG, arranged by NES.

We used a Sleeping Beauty–based (SB-based) system to induce de novo pHGG in mice through stable integration of genetic lesions in brain neural precursor cells (22–24) (Figure 1, A and B, and Supplemental Figure 1A; supplemental material available online with this article; https://doi.org/10.1172/JCI154229DS1). The H3.3-G34R mutation in human pHGG co-occurs with *TP53*- and *ATRX*-inactivating mutations (2, 4, 12), and RTK/RAS/PI3K activation is common in these tumors (2). Thus, we designed the mouse H3.3-G34R pHGG model to also express shRNAs against *p53* and *Atrx*, together with RTK/RAS/PI3K activation via a constitutively active NRAS (Figure 1B). We compared the H3.3-G34R group of mice (shP53 + shATRX + NRAS activation + H3.3-G34R) with the WT H3.3 control group (H3.3-WT) (shP53 + shATRX + NRAS activation). The H3.3-G34R mice had a median survival of 125 days (Figure 1C). We observed heterogeneous expression of the G34R mutation in mouse pHGG (Supplemental Figure 1A), similar to the expression of G34R observed in patient-derived pHGG cells (Supplemental Figure 1E). Histopathological analysis of H3.3-G34R de novo–induced SB tumors showed necrotic areas with pseudopalisades and hemorrhages, which are distinctive clinical features of HGG (Supplemental Figure 1, C and D).

We confirmed the expression of the mutant histone and the downregulation of *Atrx* in the H3.3-G34R tumors by IHC (Supplemental Figure 1B). IHC to detect phospho-ERK (phospho-Thr202/Tyr204 p44/42 MAPK, a marker of Ras/Raf/MEK/ERK signal transduction cascade activation) was performed to confirm the activation of the Ras signaling pathway in the H3.3-WT and the H3.3-G34R tumors. Ras signaling activation is a hallmark of H3.3-G34R pHGG (2). We observed no differences in phospho-ERK levels between H3.3-WT and H3.3-G34R tumors (Supplemental Figure 2, A–C). We also observed no differences in normalized phospho-ERK levels between G34R and WT pHGG cells by Western blotting (Supplemental Figure 2, D and E) in the mouse or human models. Both H3.3-G34R and H3.3-WT pHGG cells were positive for Nestin (Supplemental Figure 3, G–I), a marker of HGG (25). Also, *DLX6* upregulation was observed in H3.3-G34R pHGG (4) (Supplemental Figure 3, J–L). DLX6 has been reported to be upregulated as a result of epigenetic reprogramming induced by H3.3-G34R expression (26). Immunostainings for H3 histone marks revealed increased levels of K36Ac in H3.3-G34R tumors and no difference in K36me3 levels between H3.3-G34R and H3.3-WT tumors (Supplemental Figure 3, A–F).

To confirm our results in a human pHGG model, we developed an H3.3-G34R model based on hemispheric pHGG–derived SJ-GBM2 primary cells (Supplemental Figure 4), which harbor TP53- and ATRX-inactivating mutations (27, 28). SJ-GBM2 cells were stably transfected with constructs to express a 3X-FLAG–tagged WT H3.3 (generating the SJ-GBM2-H3.3-WT cells) or a 3X-FLAG–tagged H3.3 harboring the G34R mutation (generating the SJ-GBM2-H3.3-G34R cells) (Supplemental Figure 4). We verified the integration of the constructs by Western blotting for FLAG (Supplemental Figure 5C). Consistent with the results from the mouse pHGG model (Supplemental Figure 3, A–F), we identified upregulation on H3K36Ac in SJ-GBM2-H3.3-G34R cells compared with SJ-GBM2-H3.3-WT cells by Western blotting (Supplemental Figure 5C), and we observed no changes in H3K36me3 levels (Supplemental Figure 5C). By immunofluorescence, we identified downregulation of OLIG1, OLIG2, Nestin, and GFAP (Supplemental Figure 5, A and B) in human H3.3-G34R pHGG cells. Loss of oligodendrocyte differentiation markers is a characteristic of H3.3-G34R/V pHGG (5). These results indicate that our human model recapitulated the molecular features of H3.3-G34R/V pHGG.

### Whole RNA-Seq analysis reveals that H3.3-G34R expression correlates with the downregulation of DNA repair–related gene ontologies.

**Figure 2:**
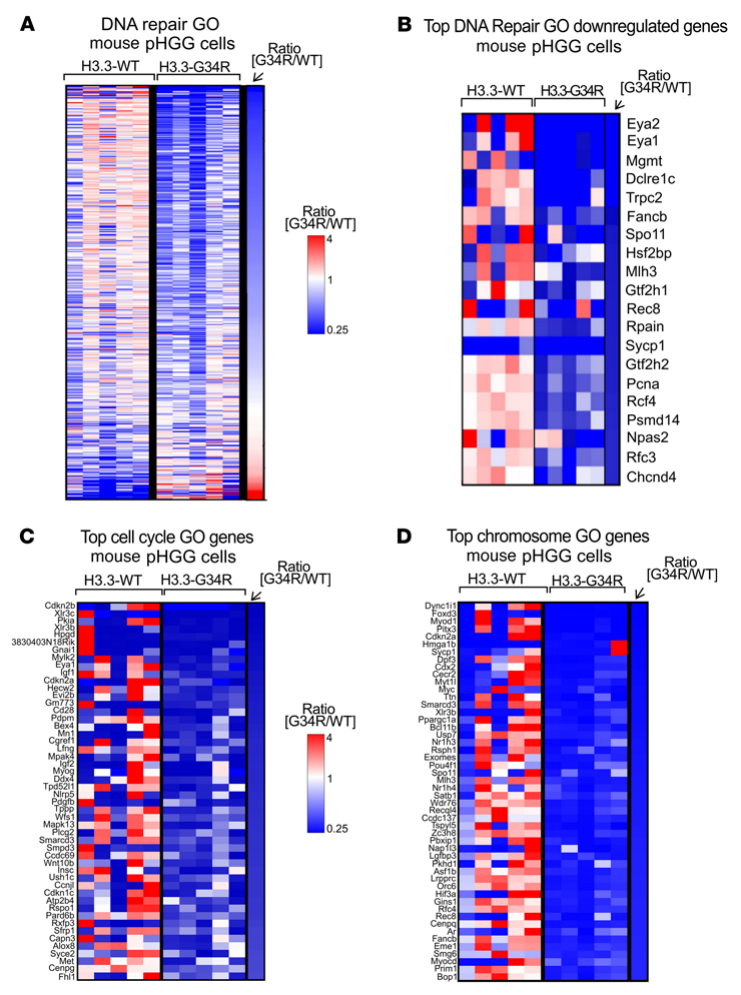
Transcriptomic analysis of relevant pathways in G34R mouse pHGG. (**A**) Heatmap depicting the expression levels of each gene within the “DNA repair” GO, comparing H3.3-G34R with H3.3-WT mouse pHGG cells. (**B**) Heatmap highlighting the top DNA repair genes that were more downregulated in H3.3-G34R than in H3.3-WT mouse pHGG cells. (**C** and **D**) Heatmaps highlighting the top genes that were more downregulated for “cell cycle” (**C**) and “chromosome” GOs (**D**) in H3.3-G34R versus H3.3-WT mouse pHGG cells.

We performed bulk RNA-Seq to compare the transcriptomes of H3.3-G34R versus H3.3-WT primary cells derived from a de novo genetically engineered mouse model (GEMM) of pHGG. We used five pHGG tumors, induced de novo using the SB system, to generate five H3.3-G34R and five H3.3-WT independent cell cultures, which we used to perform RNA-Seq. Gene set enrichment analysis (GSEA) revealed differentially expressed gene ontologies (GOs) and hallmarks in mouse H3.3-G34R pHGG cells (Figure 1D and Supplemental Figure 6). Among the GOs downregulated in H3.3-G34R cells, we found clusters associated with the response to DNA repair (Figure 2, A and B, and Supplemental Figure 7), the cell cycle (Figure 2C and Supplemental Figure 8), and chromatin structure (Figure 2D and Supplemental Figure 8). The full differential expression (DE) analysis and counts are provided in Supplemental Tables 1 and 2.

**Figure 3:**
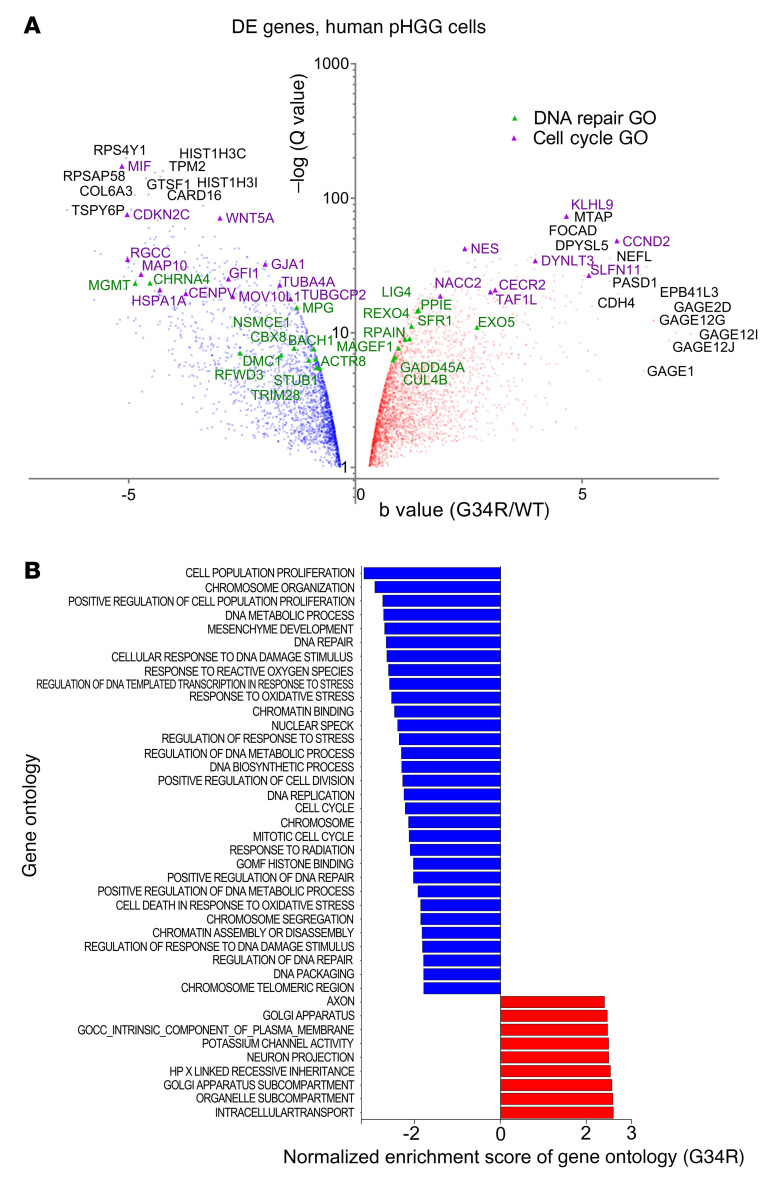
Transcriptomic analysis of a human model of H3.3-G34R pHGG. (**A**) Volcano plot of genes differentially expressed between H3.3-G34R and H3.3-WT human pHGG cells. DNA repair GO genes are highlighted in green, and cell cycle GO genes are highlighted in purple. (**B**) Selection of differentially enriched GOs between H3.3-G34R and H3.3-WT human pHGG cells, arranged by NES.

**Figure 4:**
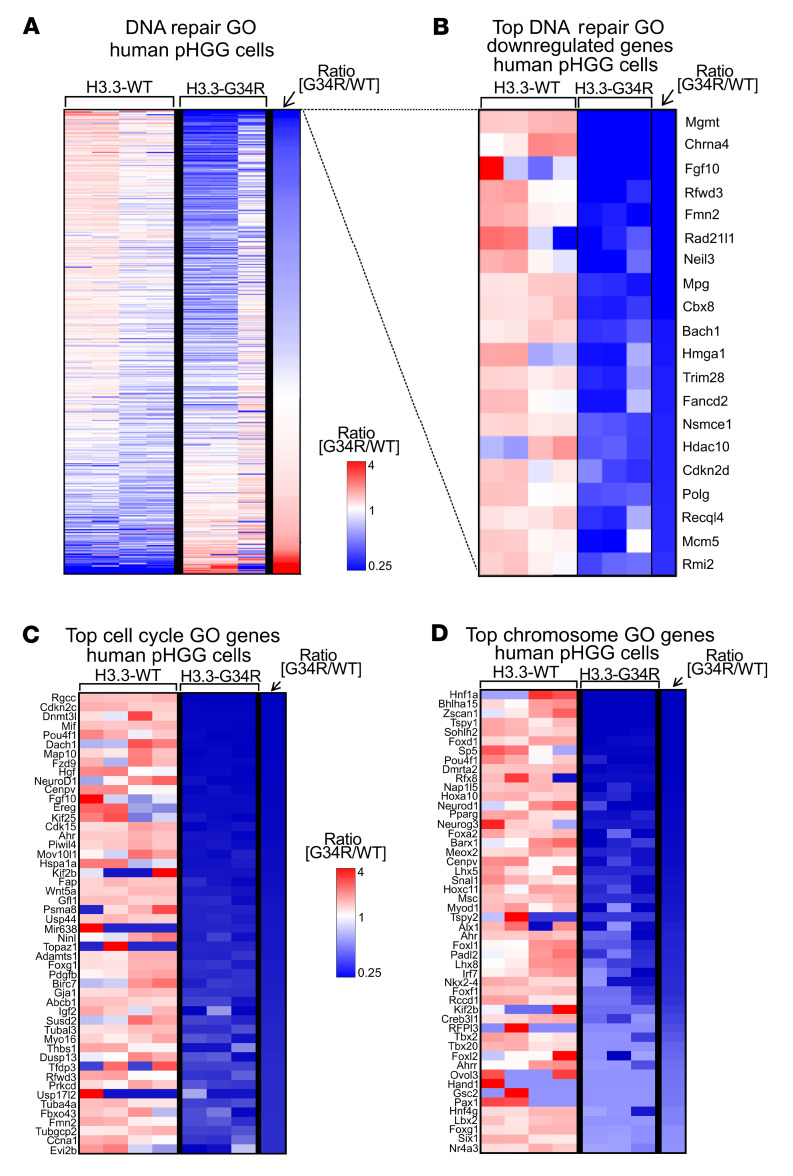
Transcriptomic analysis of relevant pathways in G34R human pHGG. (**A**) Heatmap depicting the gene expression levels of each gene within the “DNA repair” GO, comparing H3.3-G34R with H3.3-WT human pHGG cells. (**B**) Top most downregulated DNA repair GO genes in H3.3-G34R versus H3.3-WT human pHGG cells. (**C** and **D**) Heatmaps highlighting the top most downregulated genes for the GOs “cell cycle” (**C**) and “chromosome” (**D**) in H3.3-G34R versus H3.3-WT human pHGG cells.

RNA-Seq was also performed in human pHGG cells (Figure 3 and Supplemental Figure 9). We observed a high concordance with the mouse RNA-Seq results, i.e., GSEA analysis revealed that H3.3-G34R expression correlated with the downregulation of DNA repair (Figure 4, A and B, and Supplemental Figure 10) as well as cell-cycle– and chromatin-related GOs (Figure 4, C and D, Supplemental Figure 8, and Supplemental Table 3 shows the full DE analysis).

RNA-Seq, genomic, and clinical data from pediatric patients with HGG from public databases (29, 30) revealed similar results (Supplemental Figure 11; the criteria applied to assign patients to the H3.3-WT group are detailed in S7 of Supplemental Methods). We found a significant decrease in the expression levels of DNA repair–related genes in the H3.3-G34R/V patient group compared with the H3.3-WT patient group (Supplemental Figure 11), which is consistent with our results in the mouse and human models (Supplemental Table 5 shows the full DE analysis). Among the downregulated DNA repair genes identified in all the systems analyzed, G34-mutant pHGG showed downregulation of *MGMT* expression compared with the H3.3-WT group (Supplemental Figure 8C). MGMT catalyzes the transfer of methyl groups from *O*(6)-alkylguanine and other methylated moieties to its own molecule, repairing the lesions caused by alkylating agents, i.e., TMZ. Thus, *MGMT* downregulation has been associated with an increased response to TMZ when combined with RT in glioma (31).

### H3.3-G34R expression correlates with reduced proliferation in vitro and in vivo.

**Figure 5:**
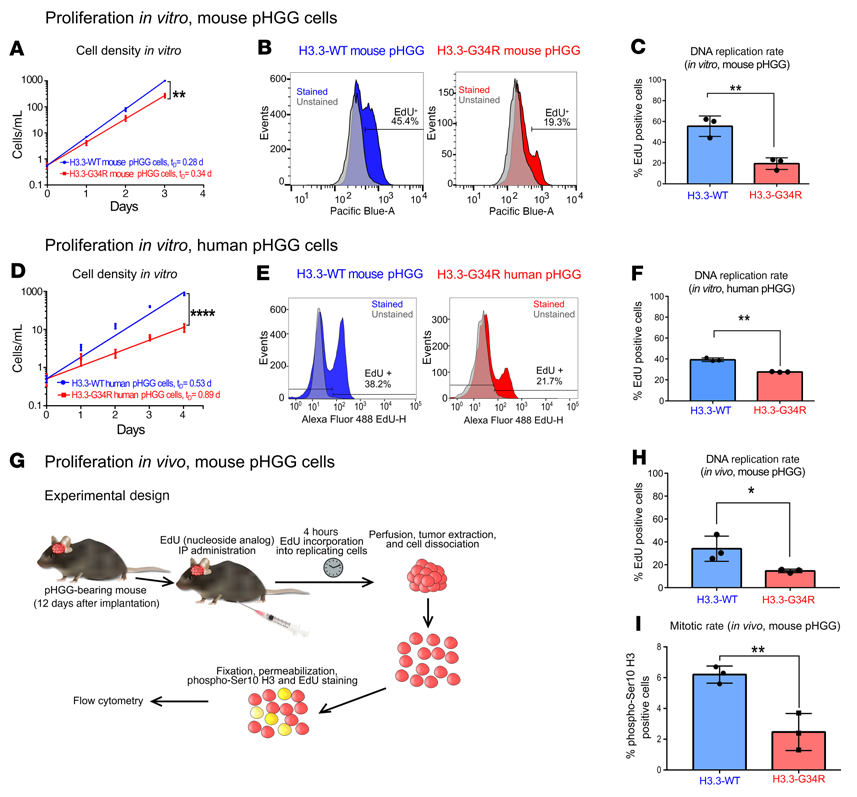
Analysis of proliferation of H3.3-G34R pHGG in vitro and in vivo. (**A**) In vitro cell growth curve comparing mouse H3.3-G34R and H3.3-WT pHGG cells. *t_D_*, cell doubling time, in days. (**B**) Analysis of the fraction of replicating cells by cytometry-based quantification of EdU incorporation comparing mouse H3.3-G34R and H3.3-WT pHGG cells. (**C**) Statistical analysis of the fraction of replicating cells from **B**. (**D**) In vitro cell growth curve comparing human H3.3-G34R and H3.3-WT pHGG cells. (**E**) Analysis of the fraction of replicating cells by cytometry-based quantification of EdU incorporation, comparing human H3.3-G34R and H3.3-WT pHGG cells. (**F**) Statistical analysis of the fraction of replicating cells from **E**. (**G**) Scheme depicting the experimental strategy to analyze the fraction of replicating cells and mitotic rates in H3.3-G34R and H3.3-WT mouse pHGG cells in vivo. (**H**) Statistical analysis of the fraction of replicating cells from the experiment illustrated in **G**. (**I**) Statistical analysis of the fraction pHGG cells undergoing mitosis (stained with H3 phospho-Ser10 in cells from the experiment illustrated in **G**); *P < 0.05, **P < 0.01, and ****P < 0.001; analysis of the slope difference in the nonlinear regression model (**A** and **D**); unpaired *t* test (**C**, **F**, **H**, and **I**). Data represent the mean ± SD of 3 identical experimental.

Following our observation that the expression of genes associated with the cell cycle were downregulated in H3.3-G34R mouse and human pHGG cells (Supplemental Figure 8B), we analyzed whether cell proliferation was affected by H3.3-G34R expression in vitro (Figure 5, A and D). Consistent with the transcriptome results, both mouse (Figure 5A) and human (Figure 5D) H3.3-G34R pHGG cells exhibited reduced proliferation compared with H3.3-WT cells. To confirm these results, we evaluated the percentage of cells synthesizing DNA by measuring the incorporation of 5-ethynyl-2′-deoxyuridine (EdU), a thymidine nucleoside analog, into replicating chromosomes. Both mouse (Figure 5, B and C) and human (Figure 5, E and F) H3.3-G34R–expressing cells showed a significantly reduced fraction of EdU^+^ cells (*P* < 0.01) compared with the cells from the H3.3-WT group. Additionally, we assessed DNA replication by EdU incorporation and the fraction of cells in the mitotic cell cycle phase by H3 (phospho-Ser10) staining (Figure 5, G–I), in vivo, in H3.3-G34R and H3.3-WT tumors. H3.3-G34R tumors had significantly decreased EdU incorporation and mitotic rates compared with H3.3-WT tumors, indicating a decreased level of cell proliferation (*P* < 0.05) (the gating strategy applied for these experiments is detailed in Supplemental Figure 12). These results are in accordance with the decreased Ki-67 and H3 (phospho-Ser10) levels observed in mouse H3.3-G34R tumors (Supplemental Figure 3, M–R).

### Analysis of stem cell–like features in mouse and human H3.3-G34R pHGGs.

Limiting dilution assays (LDAs) revealed that mouse and human H3.3G34R pHGG cells had a reduced colony-forming ability in vitro compared with their H3.3-WT counterparts (Supplemental Figure 13, A and B), indicating a decreased proportion of colony-initiating cells among the G34-mutant cells in the G34-mutant cells. To determine whether this was associated with a reduction in stem cell populations in H3.3-G34R cells, we evaluated the percentages of cells expressing neural stem cell markers (CD133^+^, ALDH1^+^, and CD44^+^) in the mouse and human models. Our results showed significantly reduced frequencies of CD133^+^ and ALDH1^+^ cells in mouse H3.3-G34R cells (Supplemental Figure 13, C and D) compared with frequencies in H3.3-WT cells. Consistently, we found significantly reduced frequencies of CD133^+^, ALHD1^+^, and CD44^+^ cells in human H3.3-G34R cells compared with frequencies in H3.3-WT cells (Supplemental Figure 13, E and F). The percentages of ALDH1^+^ cells among H3.3-WT and H3.3-G34R mouse pHGG cells were 99.7% and 97.4%, respectively, a finding that was statistically significant but not biologically relevant. The percentage of triple-positive cells (CD133^+^ALDH1^+^CD44^+^) was significantly lower (*P* = 0.000107) in H3.3-G34R cells than in H3.3-WT cells (Supplemental Figure 14; the gating strategy applied for these experiments is shown in Supplemental Figure 15). Additionally, we evaluated the tumor-initiating capacity in vivo by implanting H3.3-G34R and H3.3-WT mouse–derived cells into the striatum and consistently found that H3.3-G34R cells had reduced frequencies of tumor-initiating cells compared with H3.3-WT cells (Supplemental Figure 13, G and H, and statistical analysis in Supplemental Table 9).

### H3.3-G34R cells exhibit reduced basal DNA repair activity, causing delayed repair of double-stranded breaks and correlating with reduced chromatin accessibility.

**Figure 6:**
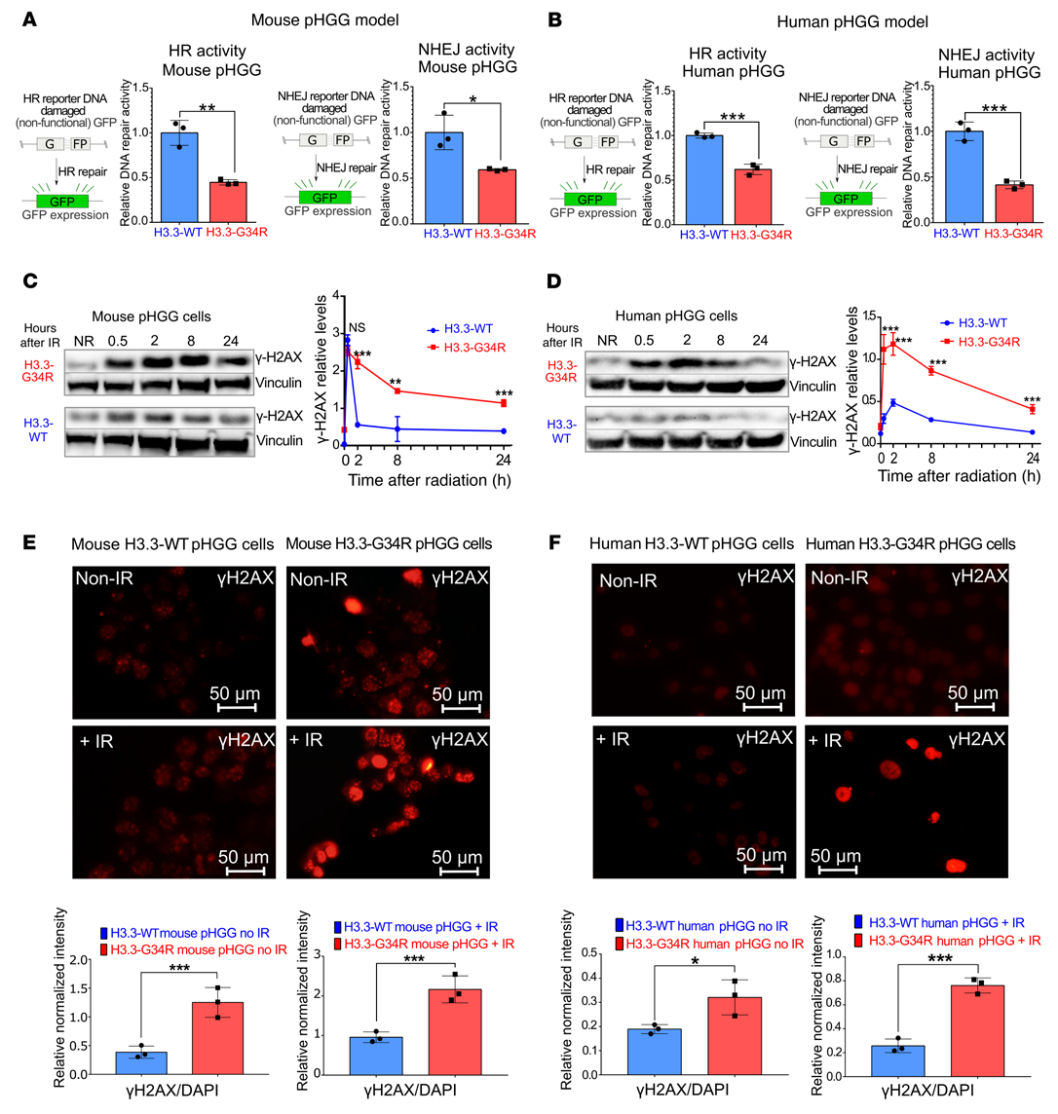
DNA repair activity is diminished in mouse G34R pHGG cells. (**A** and **B**) Schemes on the left in **A** and **B** show plasmid-based reporter assays to assess HR DNA repair activity levels. The HR plasmid was linearized to disrupt the *Gfp* gene, and the repair of the plasmid through HR reconstituted GFP expression. Graphs on the left show in **A** and **B** HR DNA repair levels in H3.3-G34R and H3.3-WT mouse (**A**) and human (**B**) pHGG cells. Schemes on the right in **A** and **B** show plasmid-based reporter assays to assess NHEJ DNA repair activity levels. The NHEJ plasmid was linearized to disrupt the *Gfp* gene, and the repair of the plasmid through NHEJ reconstituted GFP expression. Graphs on the right in **A** and **B** show NHEJ DNA repair levels on H3.3-G34R and H3.3-WT mouse (**A**) and human (**B**) pHGG cells. (**C** and **D**) Western blotting for γH2AX levels of H3.3-G34R and H3.3-WT mouse (**C**) and human (**D**) pHGG cells at different time points after 3 Gy IR. Graphs on the right show quantification of γH2AX levels from the experiment described. (**E** and **F**) Immunofluorescence images of γH2AX levels in H3.3-G34R and H3.3-WT mouse (**E**) and human (**F**) pHGG cells processed 4 hours after 3 Gy IR. Scale bars: 50 μm. Graphs on the bottom show quantification of γH2AX levels determined by immunofluorescence. *P < 0.05, **P < 0.01, and ***P < 0.005; unpaired *t* test (**A**–**F**). Data represent the mean ± SD of 3 technical replicates.

On the basis of the transcriptomic analysis evidencing downregulation of DNA repair genes in H3.3-G34R pHGG, we assessed the DNA repair activity by performing functional DNA repair assays. In these assays, cells were transfected with linearized plasmids that could be repaired either by homologous recombination (HR) or nonhomologous end-joining (NHEJ) (32). GFP expression from the HR plasmid can only be reestablished when the linearized plasmid is repaired through HR, whereas the NHEJ plasmid expresses GFP only when it is repaired through NHEJ. Both mouse and human H3.3-G34R pHGG cells (Figure 6, A and B, and Supplemental Figure 16) exhibited significantly diminished HR and NHEJ DNA repair activity when compared with their H3.3-WT counterparts. Additionally, we observed reduced HR and NHEJ activity in 2 patient-derived endogenous G34R-mutant cell cultures compared with a patient–derived H3.3-WT cell culture (Supplemental Figure 17, C and D).

To evaluate whether impaired DNA repair activity has an impact on the kinetics of DNA damage repair, we evaluated the repair of double-stranded breaks (DSBs) by measuring γH2AX [H2AX (phospho-Ser139)] levels at different time points after IR treatment in vitro in mouse and human pHGG cells (Figure 6, C and D). H2AX is normally phosphorylated in response to DSBs, thus γH2AX is a sensitive target for assessing the presence of DSBs in cells. The decrease in γH2AX levels, after its initial accumulation, indicated the repair of the DSB lesions. We observed slower DNA repair in mouse and human H3.3-G34R cells compared with the H3.3-WT counterpart cells, as evidenced by the persistence of high γH2AX levels at later time points in H3.3-G34R cells after IR. We also performed immunofluorescence in nonirradiated and irradiated mouse and human cells (4 hours after 3 Gy IR) and likewise observed increased γH2AX in H3.3-G34R pHGG cells (Figure 6, E and F). These results were further confirmed by a neutral comet assay (Supplemental Figure 18), in which we observed increased DSB signals 4 hours after IR (3 Gy) in mouse and human H3.3-G34R cells compared with H3.3-WT controls.

**Figure 7:**
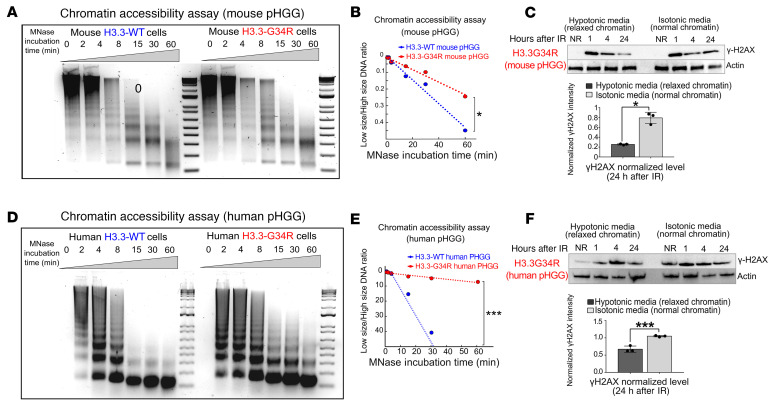
Chromatin accessibility is reduced in G34R pHGG. (**A**) DNA gel depicting chromatin accessibility analyzed by timed MNase digestion of chromatin from H3.3-G34R and H3.3-WT mouse pHGG cells. (**B**) Statistical analysis of MNase digestion from **A**. (**C**) Western blot depicting γH2AX levels in response to IR under normal conditions (isotonic media) or under conditions that favored chromatin relaxation in H3.3-G34R and H3.3-WT mouse pHGG cells. Graph shows statistical analysis of γH2AX levels 24 hours after IR. (**D**) Chromatin accessibility analyzed by timed MNase digestion of chromatin from H3.3-G34R and H3.3-WT human pHGG cells. (**E**) Statistical analysis of MNase digestion of chromatin from **D**. (**F**) Western blot depicting γH2AX levels in response to IR under normal conditions (isotonic media) or under conditions that favored chromatin relaxation in H3.3-G34R and H3.3-WT human pHGG cells. Graph below shows statistical analysis of the γH2AX levels 24 hours after IR. *P < 0.05 and ***P < 0.005; unpaired *t* test (**C** and **F**); analysis of the slope difference in the nonlinear regression model (**B** and **E**). Data represent the mean ± SD of 3 technical replicates (**C** and **F**).

Next, we asked whether the chromatin structure imposed by H3.3-G34R expression played a role in the DNA repair deficiency of these cells. To evaluate whether H3.3-G34R expression alters the compactness of the chromatin, we performed MNase accessibility assays. In these experiments, chromatin is digested for increasing durations with MNase, and the fraction of digested chromatin over time is reflective of its accessibility. In 3 different systems: (a) mouse-derived cells harboring H3.3-G34R or H3.3-WT (Figure 7, A and B); (b) pHGG human cells stably transfected with H3.3-WT or H3.3-G34R (Figure 7, D and E); and (c) patient-derived stem cell cultures with an endogenous H3.3-G34R mutation versus a H3.3-WT pHGG (Supplemental Figure 17, A and B), we found that cells expressing H3.3-G34R mutations had higher levels of chromatin compaction. We hypothesized that an increase of the chromatin compactness in H3.3-G34R cells could affect the accessibility of DDR proteins to the DNA damaged sites. To evaluate this hypothesis, we assessed DNA repair kinetics after IR under normal conditions and under conditions that induced chromatin relaxation in mouse and human H3.3-G34R cells. To induce chromatin relaxation, we reduced the osmolarity of the growth media from the isotonic value (150 mM) to hypotonic conditions (75 mM) (33). We observed that the DNA repair of DSBs in hypotonic conditions was faster than in isotonic conditions in mouse (Figure 7C) and human (Figure 7F) H3.3-G34R pHGG cells, demonstrating that chromatin relaxation improved DNA repair efficiency in the G34-mutant cells.

### Activation of DNA repair pathways in mouse and human H3.3-G34R pHGG cells in response to IR-induced DNA damage.

We characterized the activation of DNA repair proteins upon IR-induced DNA damage. We performed Western blotting to measure the levels of proteins and posttranslational modifications (PTMs) involved in DNA damage sensing (ATM, ATM phospho-Ser1981); HR repair (RAD51, RAD51 phospho-Thr309; FANCD2, FANCD2 phospho-Thr691; RPA32, RPA32 phospho-Ser33); NHEJ (DNA-PKs, DNA-PKs phospho-Ser2056; 53BP1; Ku80, Ku80 phospho-Thr714); and DSB-induced cell cycle checkpoint activation (CHK2, CHK2 phospho-Thr68) (Supplemental Figure 19, A and B). Our data revealed that the levels of PTMs that indicate activation of the DDR proteins remained high at later time points after IR in H3.3-G34R human cells, while they returned to basal levels earlier for H3.3-WT cells (Supplemental Figure 19B) in the pHGG models. These results indicate that the DNA was not efficiently repaired in G34-mutant cells and that the DDR machinery needed more time to repair the DNA damage in these cells. We also analyzed cell cycle CHK2 phospho-Thr68 levels by immunofluorescence in human pHGG cells and observed higher levels 12 and 96 hours after irradiation in H3.3-G34R cells than in H3.3-WT cells (Supplemental Figure 20).

### High-throughput profile of proteins and PTMs related to the DDR in mouse and human H3.3-G34R pHGG cells.

**Figure 8:**
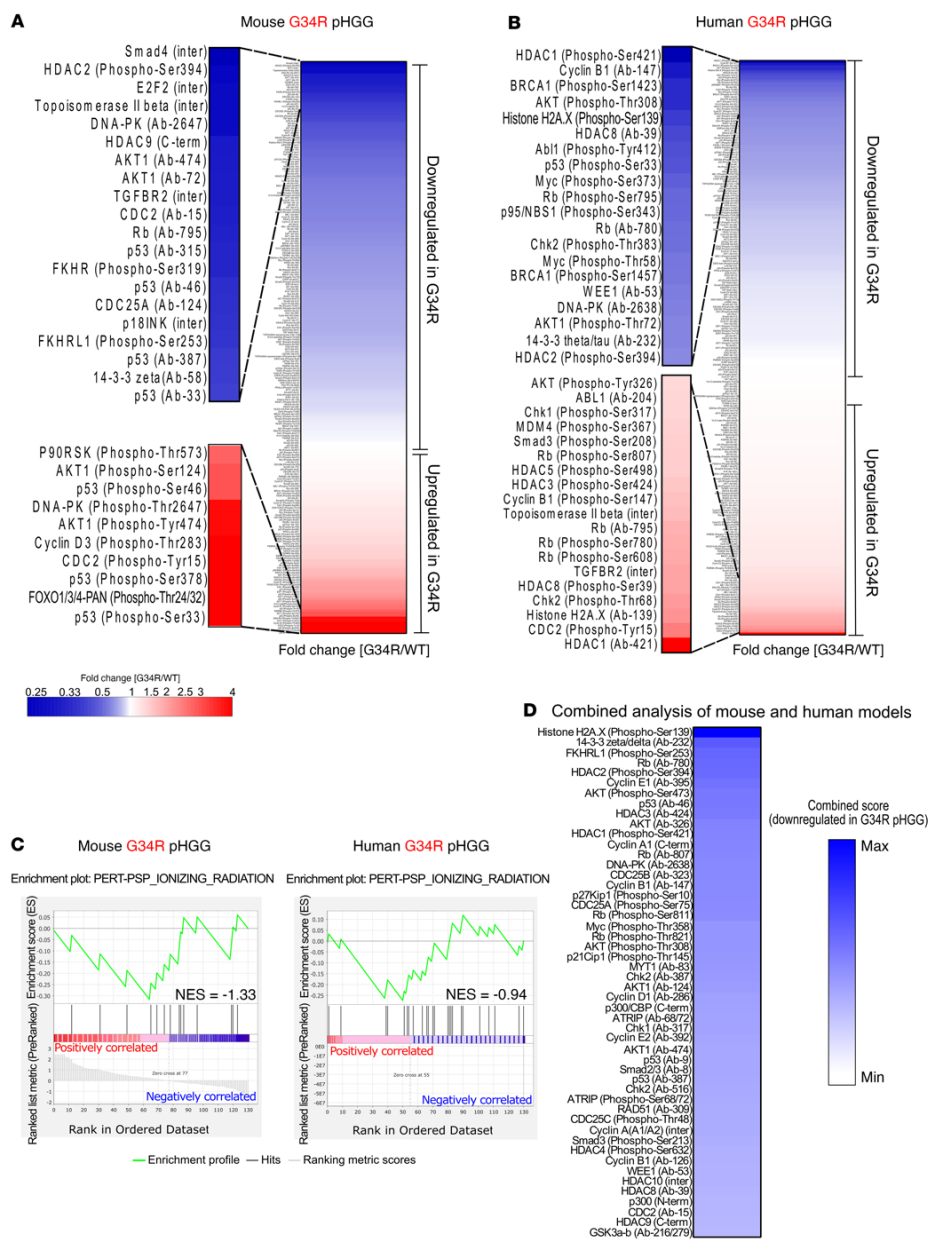
Analysis of DNA repair pathway activity according to protein and PTM levels in G34R pHGG cells. (**A**) Heatmap showing the results of total DNA repair proteins and PTM levels in G34R mouse pHGG cells versus H3.3-WT cells, highlighting the main upregulated and downregulated proteins and PTMs. (**B**) Heatmap showing the results of total DNA repair proteins and PTM levels in G34R human pHGG cells versus WT cells, highlighting the main upregulated and downregulated proteins and PTMs. (**C**) Result of GSEA using the PTM signature PSP_IONIZING_RADIATION (a signature composed of PTMs induced by irradiation of cells). (**D**) Heatmap of a combined analysis highlighting the marks that were most downregulated in G34R pHGG cells in both the mouse and human models.

To further characterize the DNA repair and cell cycle responses in G34-mutant cells, we used a phospho-array that allows characterization of the levels of multiple DNA repair and cell cycle–associated proteins and their corresponding PTMs. We compared the results from H3.3-G34R and H3.3-WT mouse (Figure 8A) and human (Figure 8B) pHGG cells and observed a global downregulation of DNA repair and cell cycle total proteins and their corresponding posttranslationally activated forms in H3.3-G34R cells. More specifically, we performed PTM signature enrichment analysis (PTM-SEA) with the PTM Signatures Database (PTMsigDB) (34) ontology “ionizing radiation.” The “ionizing radiation” PTM signature is composed of PTMs that are upregulated upon IR-mediated DNA damage. Our analysis indicates that this signature was downregulated in H3.3-G34R mouse (normalized enrichment score [NES] = –1.33) and human (NES = –0.94) pHGG cells (Figure 8C). The reduced number of targets in this PTM signature did not allow for a robust significance analysis. We performed a combined analysis to identify the marks and proteins that were downregulated in both the mouse and human models (Figure 8D). We annotated the relative protein and PTM levels in H3.3-G34R over H3.3-WT cells to the “DNA repair” pathway network, which showed downregulation of the proteins and PTMs in this pathway in the G34-mutant cells (Supplemental Figure 21). Full analyses of the results of the protein arrays performed in human and mouse pHGG cells are provided in Supplemental Tables 10 and 11, respectively.

### DNA repair impairment increases the susceptibility of H3.3-G34R pHGG to DNA damage and DDRi.

**Figure 9:**
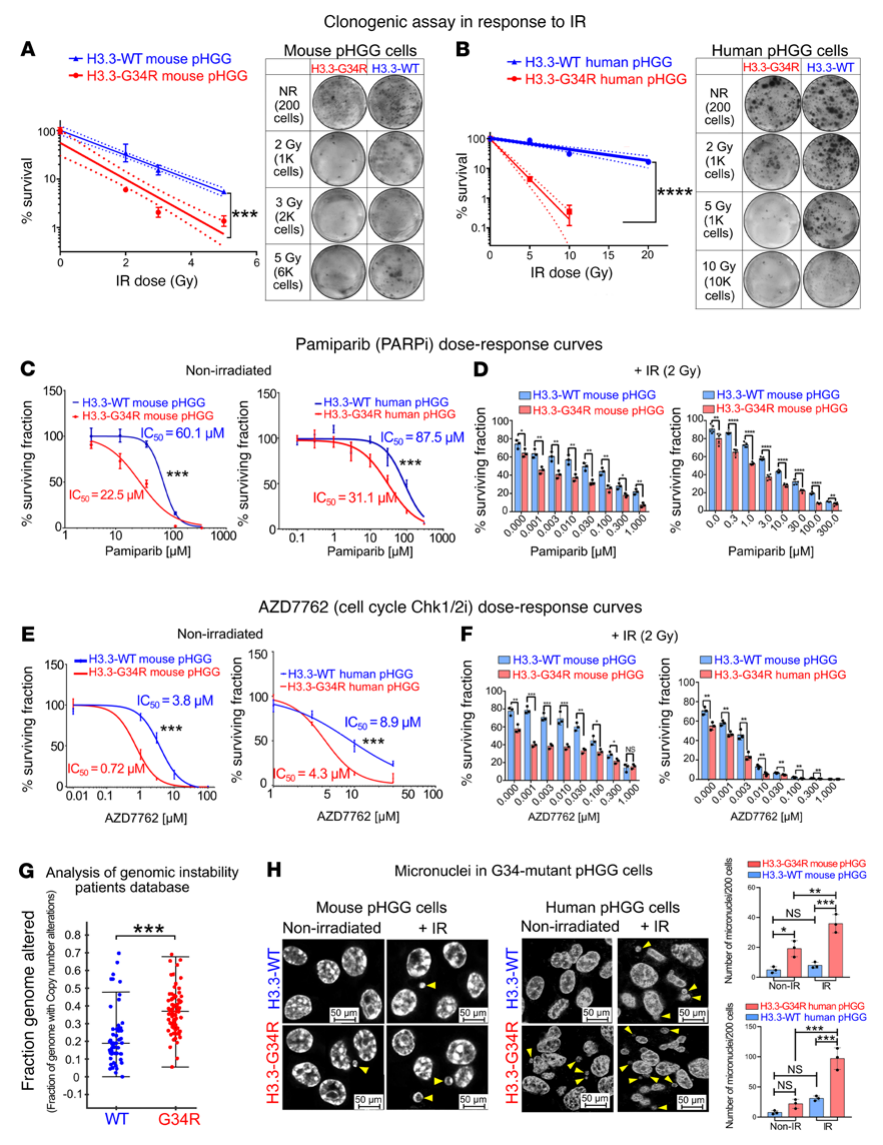
H3.3-G34R pHGG cells exhibit increased susceptibility to IR and to DDRi in vitro. Clonogenic assay in H3.3-G34R and H3.3-WT mouse (**A**) and human (**B**) pHGG cells in response to increasing doses of IR. NR, no radiation. (**C**) Dose-response curves of H3.3-G34R and H3.3-WT mouse and human pHGG cells in response to the PARPi pamiparib. (**D**) Survival of H3.3-G34R and H3.3-WT mouse and human pHGG cells in response to the combination of IR and increasing doses of pamiparib. (**E**) Dose-response curves of H3.3-G34R and H3.3-WT mouse and human pHGG cells in response to the CHK1/2i AZD7762. (**F**) Survival of H3.3-G34R and H3.3-WT mouse and human pHGG cells in response to the combination of IR and increasing doses of AZD7762. (**G**) Analysis of the CNAs of genomes of patients carrying H3.3-WT or H3.3-G34R pHGG, from the PedcBioPortal database. (**H**) Analysis of the number of micronuclei in H3.3-G34R and H3.3-WT mouse and human pHGG cells under basal conditions and after IR. Yellow arrowheads indicate micronuclei. Scale bars: 50 μm. *P < 0.05, **P < 0.01, ***P < 0.005, and ****P < 0.001; analysis of the slope difference in the nonlinear regression model (**A** and **B**); the survival fraction for each irradiation dose was calculated for each irradiated plate as follows: (colonies on nontreated plate)/(cells plated on nontreated plate)/(colonies on case plate)/(cells plated on case plate); analysis of the IC_50_ difference in the sigmoid nonlinear regression model (**C** and **E**); unpaired *t* test (**D** and **F**), Wilcoxon test (**G**), and 1-way ANOVA with Šidák’s multiple-comparison correction (**H**). Data represent the mean ± SD of 3 identical experiments (**A**–**F**).

To further evaluate the effects of H3.3-G34R expression on DNA repair, we compared the sensitivity of in vitro–cultured cells to IR by performing colony formation assays after subjecting the cells to incremental doses of IR. Both mouse (Figure 9A) and human H3.3-G34R (Figure 9B) pHGG cells showed increased sensitivity to IR when compared with H3.3-WT cells, as reflected in the reduced number of colonies formed.

Taking into consideration the diminished DNA repair activity in cells harboring the H3.3-G34R mutation, we assessed the susceptibility of in vitro–cultured cells to the poly ADP ribose polymerase inhibitor (PARPi) pamiparib. PARPi are DDRi known to sensitize cells that have diminished DNA repair responses (35). We assessed the effects of pamiparib on our mouse and human pHGG cells (Figure 9C). We also evaluated an additional syngeneic H3.3-G34R model with an alternative genetic background (*Cdkn2a* deletion, PDGFRα constitutively active mutation, *ATRX* and *P53* downregulation) (Supplemental Figure 22D), and human pHGG cells with endogenous expression of H3.3-G34R (Supplemental Figure 22F). In all systems, H3.3-G34R cells were more susceptible to pamiparib when compared with their respective H3.3-WT controls. When the PARPi pamiparib was combined with IR, H3.3-G34R cells were more susceptible to the PARPi at all the doses evaluated compared with their H3.3-WT counterparts for all IR doses (Figure 9D).

We also evaluated the effect of the cell cycle checkpoint kinase 1/2 inhibitor AZD7762 in 3 systems: mouse-derived H3.3-G34R pHGG cells, human SJ-GBM2-H3.3-G34R pHGG cells (Figure 9E), and patient-derived pHGG cells with endogenous expression of H3.3-G34R (Supplemental Figure 22E). We found that H3.3-G34R cells were more susceptible to AZD7762 when compared with their respective H3.3-WT controls. When cell cycle checkpoint inhibition was combined with IR (Figure 9F), H3.3-G34R cells were more susceptible to AZD7762 at all the doses evaluated compared with their H3.3-WT counterparts for all IR doses.

Our RNA-Seq analyses revealed that *MGMT* was one of the most downregulated DNA repair–related genes in the H3.3-G34R group found in our mouse and human pHGG models (Supplemental Figure 8C). Moreover, our analysis of patient databases indicated reduced *MGMT* expression in patients with H3.3-G34R/V pHGG compared with patients with other hemispherical pHGGs (Supplemental Figure 22H). The protein produced by the *MGMT* gene catalyzes the transfer of methyl groups from *O*(6)-alkylguanine and other methylated moieties to its own molecule, repairing the lesions caused by alkylating agents, such as TMZ. *MGMT* epigenetic silencing has been associated with increased susceptibility to TMZ in glioma (31). We evaluated the effect of TMZ in G34-mutant mouse and human pHGG cells. In both systems, H3.3-G34R expression led to increased susceptibility to TMZ (Supplemental Figure 22, A and B). Additionally, we performed clonogenic assays to further assess the effect of TMZ on H3.3-G34R pHGG cells. Our results demonstrated that H3.3-G34R mouse (Supplemental Figure 23, A and C) and human pHGG cells (Supplemental Figure 23, B and D) were more susceptible to TMZ than were H3.3-WT pHGG cells.

### Analysis of human databases reveals that patients with H3.3-G34R/V exhibit increased genomic rearrangements.

The compromised DDR seen in the H3.3-G34R group prompted us to analyze the effects of the H3.3-G34R mutation on the genomic integrity in patient pHGGs. We compared the number of mutations and the number of genomic rearrangements between the H3.3-G34R/V and H3.3-WT groups and observed that H3.3-G34R/V tumors had a higher number of copy number alterations (Figure 9G), while there were no significant differences in the number of single nucleotide mutations (Supplemental Figure 22G). In line with these results, we analyzed the levels of micronuclei in H3.3-G34R cells in both mouse and human models (Figure 9H). Micronuclei arise from acentric chromosome fragments that result from genomic rearrangements. As these fragments are unable to migrate to the mitotic spindle poles during the cell cycle anaphase, they are not incorporated into either of the daughter nuclei, thus emerging in the cytoplasm. Micronuclei are therefore indicative of genomic instability. We observed an increased number of micronuclei in the G34-mutant cells compared with their respective H3.3-WT controls under basal conditions (in the mouse model) and after IR (in both models) (Figure 9H). These results indicate increased genomic instability in H3.3-G34R pHGG cells.

### Impairment of DNA repair in H3.3-G34R pHGG cells correlates with increased susceptibility of cells to RT-induced damage in vivo.

**Figure 10:**
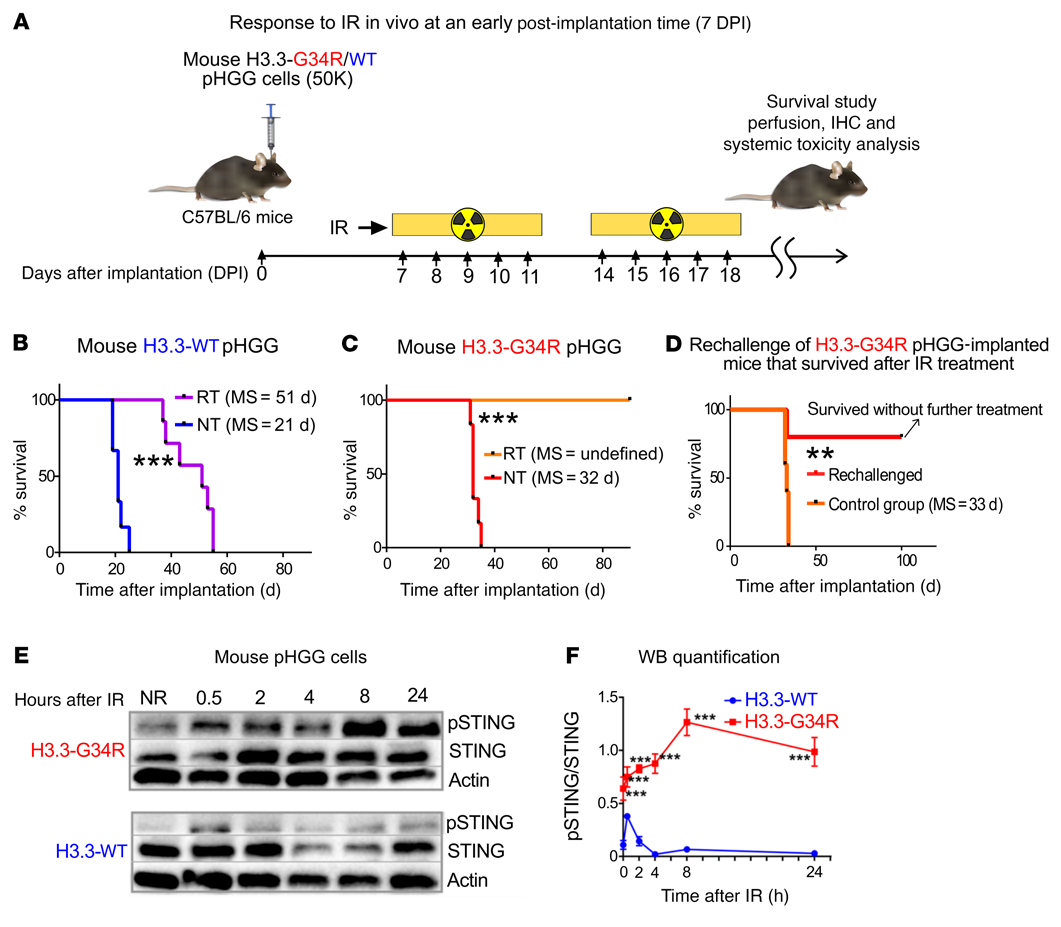
H3.3G34R pHGG shows an improved therapeutic response to RT, and DNA damage in these cells mediates cGAS/STING pathway activation. (**A**) H3.3-G34R and H3.3-WT mouse cells were implanted to generate allogenic pHGG in mice. Mice were subjected to 20 Gy RT starting on day 7 after implantation according to the schedule indicated in the scheme (2 Gy/d, 10 days). (**B**) Kaplan-Meier survival plot of H3.3-WT–bearing mice treated with RT compared with NT mice. (**C**) Kaplan-Meier survival plot of H3.3-G34R–bearing mice treated with RT compared with NT mice. (**D**) Kaplan-Meier survival plot of H3.3-G34R–bearing mice that survived after RT treatment as indicated in **C** and that were rechallenged by implantation of H3.3-G34R cells into the contralateral hemisphere. (**E**) STING (phospho-Ser365) levels in H3.3-G34R and H3.3-WT mouse cells at different time points after 3 Gy IR. (**F**) Quantification of the Western blot (WB) results represented in **E**. **P < 0.01 and ***P < 0.005; analysis of MS from the Kaplan-Meier curve; *n* = 5 mice/group (**B**–**D**). Data were analyzed by log-rank (Mantel-Cox) test (**B**–**D**) and unpaired *t* test (**F**). Data in **F** represent the mean ± SD of 3 technical replicates.

To assess the response of H3.3-G34R pHGGs to RT, H3.3-G34R and H3.3-WT cells were implanted into the mouse striatum, and mice were subjected to RT. We treated H3.3-G34R pHGG–bearing mice at an early point, i.e., 7 days, after implantation, when the tumors were small (Figure 10A). All of the mice implanted with H3.3-WT pHGG cells reached an endpoint, with a median survival (MS) of 51 days, whereas all mice implanted with H3.3-G34R pHGG cells treated with IR achieved long-term survival (Figure 10, B and C). When the surviving mice implanted with H3.3-G34R pHGG cells were rechallenged with a second implantation of H3.3-G34R tumor cells in the contralateral hemisphere, 80% of the animals did not develop tumors (Figure 10D and Supplemental Figure 24, A and B). This indicates that the mice that survived following RT developed antitumor immunological memory.

### Activation of the cGAS/STING pathway and release of damage-associated molecular patterns are exacerbated in H3.3-G34R pHGG cells upon DNA damage.

**Figure 11:**
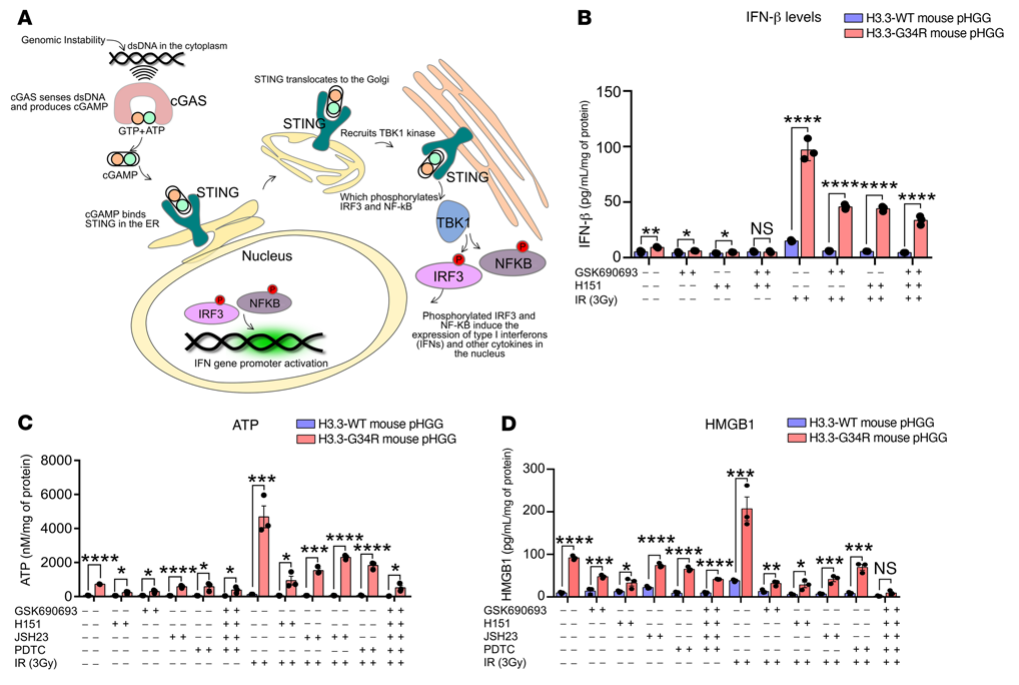
H3.3G34R pHGG mediates cytokine and DAMP release via cGAS/STING pathway activation. (**A**) Scheme illustrating the cGAS/STING pathway and the link between cytosolic dsDNA and activation of the immune system. (**B**) Release of IFN-β in H3.3-G34R and H3.3-WT mouse cells in response to 3 Gy IR and inhibition of IFN-β release by the STING inhibitors GSK690693 and H151. (**C**) Levels of soluble DAMP ATP in H3.3-G34R and H3.3-WT mouse cells in response to 3 Gy IR and inhibition of the cGAS/STING pathway with GSK690693 (STING-dependent IRF3 activation inhibitor); H151 (STING inhibitor); JSH23 (NF-κB activation inhibitor); and PDTC (NF-κB inhibitor). (**D**) Levels of soluble DAMP HMGB1 in H3.3-G34R and H3.3-WT mouse cells in response to IR and inhibition of the cGAS/STING pathway with the inhibitors GSK690693, H151, JSH23, and PDTC. *P < 0.05, **P < 0.01, ***P < 0.005, and ****P < 0.001; unpaired *t* test (**B**–**D**). Data in **B**–**D** represent the mean ± SD of 3 experimental replicates.

We demonstrated that H3.3-G34R pHGG cells had increased genomic instability (Figure 9, G and H). It was shown that genomic instability can lead to activation of the cGAS/STING signaling pathway in cancer cells (36). Therefore, we asked whether this pathway, which senses aberrant cytosolic dsDNA to induce the expression of type I IFNs, is more active in H3.3-G34R pHGG cells. We analyzed STING and STING phospho-Ser366 levels in G34-mutant and WT mouse and human pHGG cells upon DNA damage induced by IR. We observed higher levels of phospho-STING (Ser366) in H3.3-G34R mouse (Figure 10, E and F) and human (Supplemental Figure 25, A and B) pHGG cells. We also analyzed the levels of secreted IFN-β, an effector of the cGAS/STING pathway (Figure 11A), in response to IR and observed increased levels of this cytokine in mouse (Figure 11B) and human (Supplemental Figure 25C) H3.3-G34R pHGG cells, both in basal conditions and upon DNA damage induced by IR. Moreover, we demonstrated that the IR-dependent increase in IFN-β levels in H3.3-G34R cells could be reversed by inhibition of the cGAS/STING pathway with GSK690693 (STING-dependent IRF3 activation inhibitor) and H151 (STING inhibitor). These results indicate that IFN-β release after DNA-damaging treatment was cGAS/STING pathway dependent.

Our data indicate that H3.3-G34R pHGG were more susceptible to IR-induced DNA damage, which leads to cGAS/STING pathway activation. This observation suggests that IR-induced DNA damage mediated the development of immunological memory against G34R pHGG. We hypothesized that the immunological memory is mediated by the stimulation of effective antiglioma immunity induced by cGAS/STING activation and in situ immunogenic cell death (ICD). ICD is elicited by exposure of antigen-presenting cells to damage-associated molecular patterns (DAMPs) in the tumor microenvironment (TME), which can lead to antitumor immunity. To test this hypothesis, we analyzed DAMP levels in H3.3-G34R and H3.3-WT mouse and human pHGG cells in response to IR. Additionally, we assessed the effect of 4 cGAS/STING inhibitors on IR-induced release of DAMPs. Our results indicate that the release of ATP and HMGB1 (representative DAMPs) was higher in H3.3-G34R mouse (Figure 11, C and D) and human (Supplemental Figure 25, D and E) pHGG cells under basal conditions and after IR, and that the IR-dependent release of these DAMPs was reduced in the presence of cGAS/STING pathway inhibitors (GSK690693 and H151, STING inhibitors, and JSH-23 and NF-κB inhibitors). This indicates that the stimulation of DAMPs released upon IR-induced DNA damage was in part mediated by the cGAS/STING pathway, which in turn was activated by the intrinsic genomic instability of H3.3-G34R pHGG cells.

### The combination of radiotherapy and DDRi improves survival and elicits immunological memory in H3.3-G34R pHGG–bearing mice.

**Figure 12:**
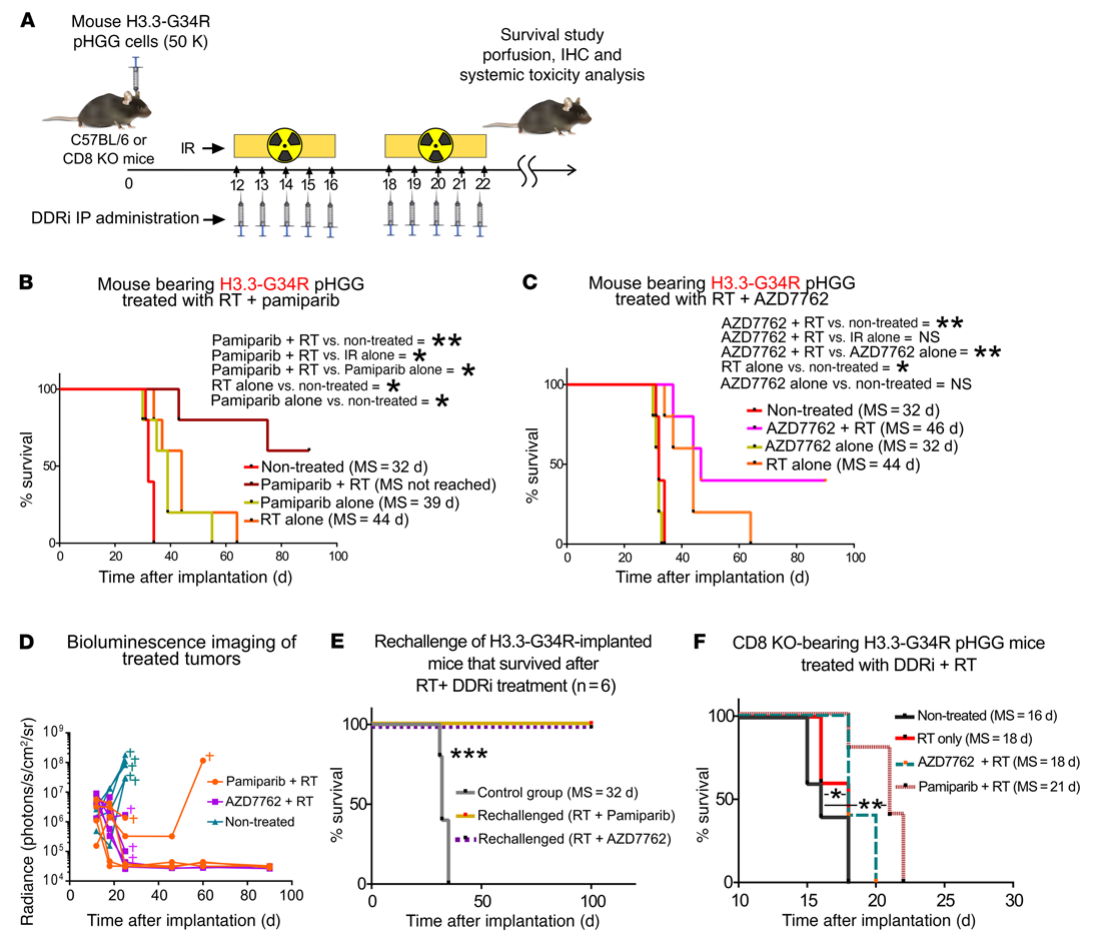
H3.3-G34R pHGG shows an improved therapeutic response to DDRi in combination with RT, and long-term survivors acquire antitumor immunological memory. (**A**) Illustration depicting the time frame of the combined treatment with DDRi and RT. (**B**) Kaplan-Meier survival plot of H3.3-G34R–bearing mice treated with RT alone or in combination with the PARPi pamiparib. (**C**) Kaplan-Meier survival plot of H3.3-G34R–bearing mice treated with RT alone or in combination with the CHK1/2i AZD7762. (**D**) Results of the in vivo imaging of tumor size in response to the DDRi plus RT treatment. The mark indicates that the animal was euthanized because of signs of tumor burden. (**E**) Kaplan-Meier survival plot of H3.3-G34R–bearing mice that survived following RT plus DDRi therapies and that were rechallenged with H3.3-G34R pHGG cells, compared with naive mice implanted with the same cells (control group). (**F**) Kaplan-Meier survival plot of CD8-KO mice implanted with H3.3-G34R cells and treated with RT alone or in combination with the CHK1/2i AZD7762 or the PARPi pamiparib. *n* = 5 mice/group. *P < 0.05, **P < 0.01, and ***P < 0.005; log-rank (Mantel-Cox) test.

We next assessed the therapeutic efficacy of combining radiotherapy (RT) with DDRi in H3.3-G34R pHGG–bearing mice. Mice were implanted with H3.3-G34R pHGG cells, and the treatment was started 12 days after implantation (Figure 12A), the point at which the tumors were large enough to render RT ineffective as a monotherapy, thus preventing long-term survival as a result of the RT. We chose these conditions in which RT only extended survival to emulate the treatment conditions of patients for whom RT is not curative. Mice were subjected to the following treatments: vehicle control, RT only, or RT plus DDRi. We used the DDRi pamiparib (PARPi) and AZD7762 (CHK1/2i) that we previously evaluated in vitro. The combination of RT plus pamiparib led to long-term survival of 60% of the mice, and therefore MS was not reached in this treatment group (Figure 12, B and D, and Supplemental Figure 26). The combination of RT plus AZD7762 led to long-term survival of 40% of the mice. The treatment with pamiparib alone was found to improve survival compared with the saline-treated group, whereas the survival of mice treated with AZD7762 alone was not significantly different from the survival of the control group mice (Figure 12, C and D, and Supplemental Figure 26). Altogether, the results indicate that the therapeutic efficacy of RT could be improved by combining it with a DDRi and that pamiparib was more efficient alone and in combination with RT than was AZD7762. We also analyzed the toxicity of the treatment by assessing liver histology, complete blood counts (CBCs), and brain architecture in surviving mice treated with DDRi plus RT (Supplemental Figure 24, F–I). We observed no histological abnormalities in livers between the nontreated (NT) animals and those treated with RT plus DDRi, and there was no deviation from CBC levels in mice treated with RT plus DDRi compared with NT animals. When long-term survivors in each treatment group were rechallenged in the contralateral cerebral hemisphere with H3.3-G34R pHGG cells, none of the animals developed tumors (Figure 12E and Supplemental Figure 24, C–E). The ability of the treatment to lead to long-term survival, and the fact that these animals did not develop tumors after tumor rechallenge, indicates that the combination of RT plus DDRi not only caused in situ damage to the tumor cells, but also that the cGAS/STING-mediated cytokine release and ICD mechanisms could contribute to the development of an adaptive immune response. To evaluate the importance of the immune system in the therapeutic response, we assessed the efficacy of the RT plus DDRi therapy in mice deficient in functional cytotoxic T cells, i.e., CD8-KO mice. Notably, the MS of H3.3-G34R pHGG–implanted CD8-KO mice with no treatment was significantly reduced when compared with that of C57BL/6 (WT) mice (Figure 12F), which indicates that the adaptive immune system played a role in delaying H3.3-G34R pHGG tumor growth. The efficacy of the therapy in CD8-KO mice was reduced, and only the RT plus pamiparib treatment significantly improved the MS from 18 days (RT alone) to 21 days (RT plus pamiparib), with no long-term survivors.

**Figure 13:**
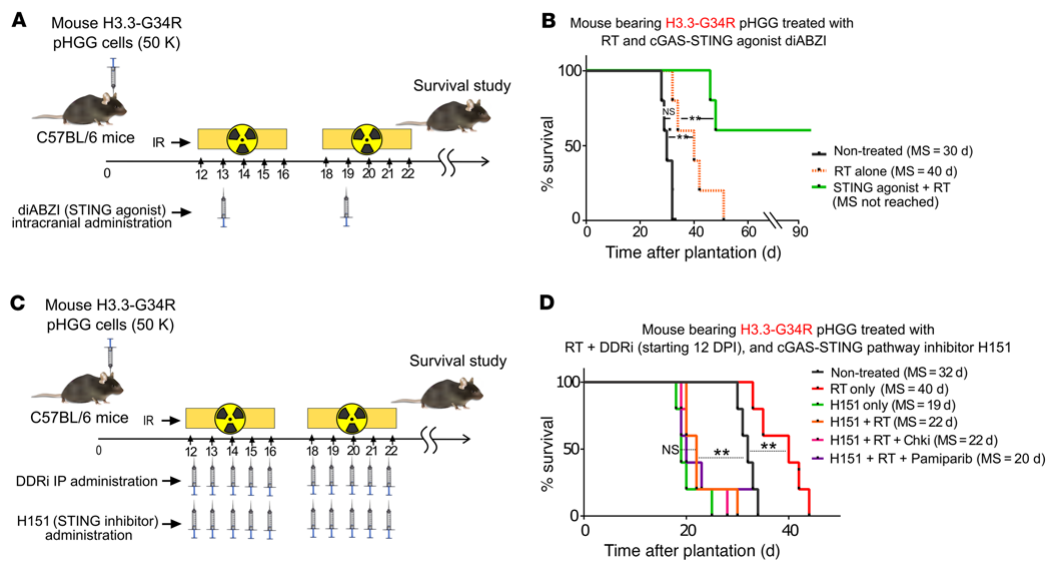
H3.3-G34R pHGG shows an improved therapeutic response to a STING agonist, and a STING inhibitor diminishes RT and DDRi efficacy. (**A**) Illustration depicting the time frame of the combined treatment with DDRi, RT, and the STING agonist diABZI. (**B**) Survival of H3.3-G34R–bearing mice treated with RT alone or with RT in combination the STING agonist diABZI. (**C**) Illustration depicting the time frame of the combined treatment with DDRi, RT, and the STING inhibitor H151. (**D**) Survival of H3.3-G34R–bearing mice that received no treatment or that were treated with RT alone; the STING inhibitor H151 alone; or with RT in combination with H151, H151 plus CHK1/2i, or H151 plus the PARPi pamiparib. *n* = 5 mice/group. **P < 0.01; log-rank (Mantel-Cox) test.

To analyze the role played by the cGAS/STING-mediated immune response in the efficacy of the RT plus DDRi therapy, we tested RT in combination with DDRi in the presence of a STING agonist and in the presence of a STING inhibitor. The combination of the STING agonist with RT (without a DDRi) significantly improved the survival of mice in this group (resulting in approximately 60% long-term survivors) in comparison with RT alone (Figure 13, A and B). The addition of the STING agonist to the RT plus DDRi (i.e., the PARPi pamiparib or the cell cycle checkpoint inhibitor AZD7762) treatment did not increase the efficacy of these treatments (Supplemental Figure 27, A–C). None of the long-term surviving animals from these treatment groups developed tumors after being rechallenged with G34-mutant pHGG cells in the contralateral hemisphere (Supplemental Figure 27D), indicating development of antitumoral immunological memory. In contrast, the STING inhibitor H151 abolished the efficacy of the combined RT plus DDRi therapy (Figure 13, C and D). Notably, the experimental group treated with the STING inhibitor alone showed reduced MS in comparison with the group of NT mice, indicating that the cGAS/STING pathway played a critical role in mediating survival. These results demonstrate that the cGAS/STING pathway played an essential role in the efficacy of the RT plus DDRi therapy.

### Veliparib, a PARPi used in pHGG clinical trials, is less potent than pamiparib in G34-mutant pHGG.

Veliparib, a blood-brain barrier–permeable (BBB-permeable) PARPi, was evaluated for non-brainstem pHGG in a clinical trial (ACNS1721) (37). This clinical trial was cancelled because of poor results, although G34R/V pHGGs were not evaluated as a separate group. We evaluated the potency of veliparib against G34-mutant pHGG. Our results demonstrate that the IC_50_ values for veliparib were consistently higher than the respective IC_50_ values for pamiparib in all the mouse and human models evaluated (Supplemental Figure 28, A–D), indicating that pamiparib was more potent than veliparib against G34-mutant pHGG in vitro. Moreover, we evaluated the combination of veliparib with RT in vivo in our mouse G34-mutant pHGG model (Supplemental Figure 28, E and F). Our results show that veliparib failed to improve the survival of the animals compared with RT treatment alone, demonstrating that veliparib was less potent than pamiparib in vivo. These results are in accordance with previous reports indicating that the potency of veliparib is reduced compared with other PARPi, including pamiparib, probably due to its relatively diminished PARP-trapping activity (38). Pamiparib (BGB-290) inhibits PARP1 and PARP2 with IC_50_ values of 0.83 and 0.11 nM, whereas veliparib (ABT-888, also referred to as NSC 737664) inhibitory constant (Ki) for PARP1 and PARP2 are of 5.2 nM and 2.9 nM (38). Pamiparib in particular exhibited potent DNA-trapping activity (with an EC_50_ of 13 nM) at a level comparable to that seen with olaparib and 30-fold more potent than veliparib (38).

## Discussion

To uncover the molecular mechanisms that lead to tumor progression and therapeutic resistance in pHGGs harboring H3.3-G34R mutations, we developed a model system that induced the formation of genetically engineered de novo pHGG in mice (Supplemental Figure 1) (23, 39). We show that the H3.3-G34R tumors induced de novo had the histopathological characteristics of pHGG (Supplemental Figures 1 and 2), and the transplantable models derived from H3.3-G34R GEMM pHGG allowed for the evaluation of experimental therapies in an immunocompetent context. The variance of the survival of our G34-mutant GEMM was comparable to the variance of the survival of patients (data not shown), demonstrating that the development and growth of pHGG in mice was heterogenous, like the human pHGG. This might be attributable in part to the well-documented tumoral intra- and interheterogeneity (40).

Altogether, our data indicate reduced basal activation of DNA repair genes and proteins and delayed activation of these responses upon DNA damage in H3.3-G34R pHGG. A link between DNA repair deficiency and the G34R-mutant histone was suggested (2, 17, 41–44) but not demonstrated in the context of pHGG, and therapies targeting these deficiencies have not been explored in the H3.3-G34R pHGG subtype. In our study, the genetic backgrounds of our H3.3-WT and H3.3-G34R models were identical, therefore we demonstrate that the impaired DNA repair was a direct consequence of H3.3-G34R expression. For example, our results indicate that H3.3-G34R expression directly caused *Mgmt* transcriptional downregulation. This observation encourages the study of the epigenetic mechanisms by which H3.3-G34R might mediate the downregulation of *Mgmt* and other DNA repair–related genes. Notably, we found that H3.3-G34R expression caused an increase in chromatin compactness, which could indicate the existence of an epigenetic mechanism mediating transcriptional silencing (45).

As oncohistone mutations cause chromatin remodeling, a possible reason for DNA repair impairment in these cells is that the H3.3-G34R mutation causes epigenetic changes that ultimately restrict or hinder the ability of DDR proteins to access damaged areas within the chromatin. We identified a global increase in chromatin compactness in pHGG cells harboring the H3.3-G34R mutation (Figures 7 and Supplemental Figure 17). Moreover, induction of chromatin relaxation improved the DNA repair efficiency in these cells (Figure 7). This indicates that accessibility to damaged DNA may be a limiting factor to DNA repair efficiency in H3.3-G34R pHGG.

To validate our molecular and functional results emerging from our models with patient-derived molecular data, we interrogated patient databases to compare data from patients with hemispheric H3.3-G34R/V with those from patients with H3.3-WT pHGG. The results indicate that the expression of DNA repair–related genes was significantly downregulated in patients with G34-mutant pHGG (Supplemental Figure 11), with *MGMT* being one of the most significant genes in this group. This indicates that patients with H3.3-G34R/V pHGG might benefit from TMZ treatment.

Our patient database analysis also demonstrated that human H3.3-G34R/V gliomas exhibit more genomic rearrangements (Figure 9G). DNA repair defects lead to DNA-damaged sites that remain unrepaired for longer durations. These sites can be improperly ligated/recombined, leading to heritable genomic rearrangements. Thus, we hypothesize that the increase in copy number alterations (CNAs) in H3.3-G34R/V pHGG is due to impairment of DNA repair.

The diminished DNA repair capability was also reflected in an increased susceptibility to IR treatments in vitro and in vivo (Figures 9 and 10). We demonstrate that IR-mediated DNA damage induced activation of the cGAS/STING pathway in H3.3-G34R pHGG (Figures 10 and 11). The cGAS/STING pathway senses the presence of aberrant dsDNA in the cytoplasm, inducing the release of type I immune-stimulatory IFNs (36, 46). In cancer cells, cytoplasmic DNA is present as a consequence of genomic instability (47). We demonstrate that G34R pHGGs exhibit genomic instability, as evidenced by an increased number of micronuclei (Figure 9H) under basal conditions and after IR treatment in mouse and human pHGG cells. We also show that the release of IFN-β was cGAS/STING dependent. Of note, activation of the cGAS/STING pathway was recently shown to negatively regulate DNA repair (48).

Tumors with DNA repair defects were shown to be more susceptible to DDRi, particularly when this treatment was combined with RT. Thus, we assessed the efficacy of DDRi in combination with RT, starting the treatment when the tumors were large enough that RT alone would not result in long-term survival. We chose these conditions, in which RT alone only extended survival, to model the conditions of patients’ responses to RT when the treatment is not curative (49). As we observed a general impairment of DNA repair in H3.3-G34R–expressing cells, affecting both HR and NHEJ, we opted not to use specific HR or NHEJ inhibitors. Thus, we selected a PARPi (pamiparib) and a CHK1/2i (AZD7762), as the radiosensitizing ability of these compounds is well established (50, 51). Our data demonstrate that the efficacy of RT both in vitro and in vivo was enhanced when used in combination with these 2 DDRi (Figures 9 and 12). The ability of pamiparib to penetrate the BBB was recently reported (38), and we demonstrated here that pamiparib was efficient against pHGG when administered intraperitoneally. The efficacy of RT in combination with the PARPi veliparib and of veliparib combined with TMZ in non-brainstem pHGG was recently evaluated (ACNS1721). The study was closed because of treatment failure or lack of adequate evidence of efficacy. Nevertheless, in this trial, G34R/V pHGGs were not stratified. ACNS1721 selected veliparib on the basis of the ability of this compound to penetrate the BBB, but the potency of the compound was reduced compared with other PARPi (38). We demonstrated here that pamiparib was more potent than veliparib against G34-mutant pHGG in vitro and in vivo, and our results showed that veliparib failed to improve the survival of the animals compared with animals treated with RT alone.

Our preclinical efficacy results indicate that DDRi plus RT was curative for more than half of the pHGG-bearing animals, and long-term survivors remained tumor free after being rechallenged in the contralateral hemisphere. This indicates that the immune responses had become active enough to eradicate any surviving tumor cells. The rechallenge experiment could be considered as a model of pHGG recurrence; thus, the treatment was able to prevent glioma reemergence in mice. Of note, the combined therapy was inefficient in CD8-KO animals (Figure 12F), indicating that the adaptive immune system played a key role in the therapeutic outcome. Our results highlight the importance of assessing the therapeutic efficacy in immune-competent models, as this allows an evaluation of the interaction of a treatment with the host’s immune system.

We showed that the accumulation of DNA damage following IR and the inability of repairing it in the presence of DDRi induce the activation of the cGAS/STING pathway, evoking an immune response. It is well established that the activation of innate immune cells is essential for the development of an adaptive memory response (52). We demonstrated here that the presence of a functional cGAS/STING pathway was essential for the success of the therapy. Moreover, cGAS/STING stimulation via a STING agonist was sufficient to improve the efficacy of RT, making STING agonists attractive candidates for therapies against G34-mutant pHGGs. Additionally, we demonstrated that DDRi plus RT can induce in situ ICD. The ICD effect was stronger in H3.3-G34R cells because of the increased susceptibility of these cells to the treatment. Additionally, the increased genomic instability in these cells could result in a higher number of neoantigens. As high radiation doses can cause long-lasting damage to the pediatric patient’s developing brain, DDRi and/or STING agonists could emerge as alternative solutions to achieving an acceptable therapeutic response at lower radiation doses in the treatment of H3.3-G34R pHGG. More generally, our results open the possibility of analyzing the interaction of immune therapies with DDRi in tumors with DNA repair defects.

While some of the effects we uncovered regarding the role of the G34R mutation in the context of tumor development might appear counterintuitive (i.e., reduced proliferation, chromatin compaction, reduced colony formation ability, etc.), we showed that G34R conferred genomic instability. Therefore, we hypothesize that G34 mutations behave as classical tumor drivers, allowing the development of cancer by facilitating the accumulation of additional genomic aberrations. A quicker acquisition of mutations or genomic rearrangements could result in tumor development, adaptation to changing conditions, development of resistance, or immune evasion. As an example of these scenarios, it is well established that germline mutations affecting DNA repair genes increase the rate of tumor incidence, thus, these mutations can be considered tumor drivers while at the same time conferring vulnerabilities to the cells (i.e., genomic instability, replication stress, reduced proliferation due to cell cycle checkpoint activation, increased susceptibility to DDR inhibitors) (53). Along this line, we showed that human G34-mutant pHGGs had increased genomic CNAs compared with H3.3-WT pHGGs. The results uncovered in this study predicted that patients with G34-mutant pHGG would respond better to the current SOC than would those with WT tumors. Nevertheless, this prediction is difficult to demonstrate, as it must be considered that there are no survival data for untreated pHGG patients, which does not allow for isolation of the treatment’s efficacy in each molecular subgroup. Additionally, few patients with histone-WT pHGG have ATRX and P53 loss-of-function mutations and lack additional mutations that might affect DNA repair. Moreover, as molecular stratification is only beginning to be a standard practice in pHGG treatment, most of the clinical trials performed to date have not separated G34-mutant pHGG as a different clinical entity, thus, the benefit of the evaluated therapies for patients with G34-mutant pHGG is not clear. The preclinical data presented in this study represent the first report to our knowledge assessing the isolated effect of the G34 mutations on the efficacy of RT and RT plus DDRi, in the context of *TP53* and *ATRX* downregulation in pHGG. Moreover, we have uncovered a prominent role for the immune system in the efficacy of DDRi therapies. In particular, we have shown that the intrinsic genetic instability of G34-mutant pHGG stimulates the immune system via cGAS/STING pathway activation. We have also shown that pharmacological stimulation of the cGAS/STING pathway via a STING agonist improved the efficacy of RT. These findings support targeting the cGAS/STING pathway as a therapeutic intervention for G34-mutant pHGG.

## Methods

Additional methods are described in the Supplemental Methods. See a detailed list of the cells used in this study in the supplemental materials.

### Reagents.

A list of the primers used in this study is provided in Supplemental Table 6, and a list of reagents used in this study is provided in Supplemental Table 7.

### Genetically engineered mouse glioma models.

To develop the main GEMM pHGG model, neonatal P01 WT C57BL/6 mice were used. We used the SB transposase system to integrate genetic lesions into the genomic DNA of neonatal mice. The following plasmids were used: (a) SB transposase and luciferase (pT2C-LucPGK-SB100X, Addgene, catalog 20207) (22, 39); (b) a short hairpin against *TP53* (pT2-shp53-GFP, Addgene, catalog 124261) (22, 39); (c) a constitutively active mutant of NRAS, NRAS-G12V (pT2CAG-NRASV12, Addgene, catalog 20205) (22, 39); (d) a short hairpin against *Atrx* (pT2-shATRX53-GFP, Addgene, catalog 124259) (23, 24); and (e) mutant H3F3A-G34R (pKT-H3.3G34R-IRES-Katushka). The genotype of pHGG generated involved the following plasmids: shp53, NRAS, and shATRX (H3.3-WT pHGG) or shp53, NRAS, shATRX, and H3.3G34R (H3.3-G34R mouse pHGG). We opted to use the H3.3-G34R mutation as representative of the G34-mutant pHGG (encompassing both H3.3-G34R and H3.3-G34V mutant pHGG), since H3.3-G34R mutations represent the most common G34 mutation on pHGG (~90%), and, to our knowledge, no mechanistic differences between H3.3-G34R and H3.3-G34V mutations have been described to date (generation of the G34R expression transposable plasmid described in S1 (Supplemental Method 1). SB/Luc, shp53, and NRAS plasmids (22) were a generous gift of John Ohlfest (University of Minnesota, Minneapolis, USA, deceased). All the plasmids’ full sequences were verified by Sanger sequencing. The in vivo transfection of SB plasmids is described in S2 (Supplemental Method 2).

### Generation of primary mouse glioma neurospheres.

Mouse glioma neurospheres were generated by harvesting brain tumors at the time of euthanasia by transcardial perfusion with Tyrode’s solution. The brains were extracted, and pHGGs generated using the SB transposon system were identified by GFP (linked to shATRX) and Katushka (RFP) expression (linked to G34R expression) with epifluorescence microscopy at the time of resection (Figure 1A). The tumor mass was dissociated using nonenzymatic cell dissociation buffer (Gibco, Thermo Fisher Scientific, catalog 13151-014), filtered through a 70 μm strainer and maintained in neural stem cell medium (DMEM/F12 with l-glutamine, Gibco, Thermo Fisher Scientific, catalog 11320-033), B-27 supplement (1×) (Gibco, Thermo Fisher Scientific, catalog 12587-010), N-2 supplement (1×) (Gibco, Thermo Fisher Scientific, catalog 17502-048), penicillin-streptomycin (100 U/mL) (Cellgro, catalog 30-001-CI), and normocin (1X) (InvivoGen, catalog ant-nr-1) at 37°C, 5% CO_2_. FGF and EGF (Shenandoah Biotech, catalogs 100-26 and 100-146) were added twice weekly at a concentration of 1 μL (20 ng/μL each stock, 1,000× formulation) per 1 mL medium. Cells expressing GFP and Katushka were sorted by flow cytometry. Five clones with each phenotype (H3.3-WT and H3.3-G34R mouse pHGG) were obtained, characterized, and used for the experiments.

### Cell culture and generation of human pHGG stably transfected cells expressing G34R and cell cultures of pHGG patient–derived cells expressing the G34R mutation.

SJ-GBM2 cells (CVCL_M141) were a gift of the COG Repository at the Health Science Center, Texas Tech University (Lubbock, Texas, USA). SJ-GBM2 cells were grown in IMDM (Gibco, Thermo Fisher Scientific, catalog 1244005320) supplemented with 20% FBS at 37.0°C, 5% CO_2_, 20% O_2_ according to a previously published report (27) and were used in early passages and tested regularly for mycoplasma. SJ-GBM2 cells were transfected using jetPRIME (Polyplus, catalog 114-01) with the plasmids pLVX-BFP-H3.3WT and pLVX-BFP-H3.3G34R described above (Supplemental Figure 4). After transfection, cells were allowed to grow for 4 days, and selected with puromycin (10 μg/μL, Goldbio, catalog P-600-10). After amplification in the presence of selection, cells were subjected to FACS for isolation of BFP^+^ cells. Three independent polyclonal populations were obtained for each genotype (SJ-GBM-2-H3.3-G34R and SJ-GBM-2-H3.3-WT). These cells are referred to as H3.3-G34R and human pHGG cells.

### RNA-Seq data availability.

The RNA-Seq data sets have been deposited in the NCBI’s Gene Expression Omnibus (GEO) database (GEO GSE182068 and GSE182069).

### Statistics.

All experiments were performed with at least 3 biological or technical replicates. Small sample number comparisons were analyzed with an unpaired *t* test. Linear regressions and nonlinear regression curves were compared by statistical tests on the parameters of the curves using the extra sum of squares *F* test. Animal experiments were performed using at least 5 animals per experimental group and were analyzed with a Mantel-Cox test. Experiments with large sample numbers (e.g., Figure 9G) were analyzed by Wilcoxon test.

### Study approval.

All animal studies were conducted according to guidelines approved by the IACUC of the University of Michigan (protocols PRO00009578 and PRO00009546). All animals were housed in an AAALAC-accredited animal facility and were monitored daily. Studies did not discriminate by sex; both male and females were used. The strains of mice used in the study were C57BL/6 (the Jackson Laboratory, strain no. 000664), CD8-KO (the Jackson Laboratory, strain no. B6.129S2-Cd8atm1Mak/J, stock no. 002665), and CDKN2A-KO mice (Frederick National Library for Cancer Research strain no. 01XB1).

## Author contributions

SH, KB, AAM, MH, FMN, MSA, PK, SC, MNB, AT, FJN, CK, EB, ST, JD, CA, MBGF, and AC performed experiments. SH, KB, AAM, MSA, VR, AR, PRL, and MGC analyzed the data. SH, KB, PRL, and MGC designed the figures. SH, KB, AAM, MH, PK, FJN, SV, CK, AR, JSY, AMC, MV, PRL, and MGC designed the research and wrote and edited the manuscript.

## Supplementary Material

Supplemental data

Supplemental tables 1, 2, 5, 6, 7, 8, 10 and 11

Supplemental table 3

## Version 1 09/20/2022

In-Press Preview

## Version 2 11/15/2022

Electronic publication
