# Supplementary material for: H3.3-G34 mutations impair DNA repair and promote cGAS/STING-mediated immune responses in pediatric high-grade glioma models
Source: J Clin Invest. 2022 Nov 15;132(22):e154229. doi: 10.1172/JCI154229 (PMC9663161; doi:10.1172/JCI154229)
Supplement: Supplemental data [file jci-132-154229-s014.pdf]

Supplementary Methods, Figures, and Tables

S1. Bulk RNA-seq of human and mouse pHGG cells

For mouse pHGG cells, we used 5 pHGG tumors induced de novo using the SB system, to generate 5 independent H3.3-G34R and 5 independent H3.3-Wt primary cultures, which we used to perform RNA-seq. For human pHGG cells, four independently generated stably transfected cultures of SJ-GBM2-H3.3-Wt and three independently generated stably transfected cultures of SJ-GBM2-H3.3-G34R were used. Biological replicates were grown in T25 flasks and two million cells were used for RNA purification. RNA extraction was performed using RNeasy Plus Mini Kit (Qiagen, Cat #74134) following the manufacturer instructions. Purified RNA (100 ng/sample) were sent for analysis. RNA quality was assessed using a TapeStation (Agilent Technologies) following the manufacturer's recommended protocol. RNA sequencing libraries were prepared according to the TruSeq Stranded Total RNA Human/Mouse/Rat (Illumina) and were sequenced on the HiSeq 2000 platform.

RNA-seq annotation and differential gene expression analysis were performed using Kallisto [1] (https://pachterlab.github.io/kallisto). Kallisto counts and statistical analysis were used to generate the Volcano plots and heatmaps of relevant gene families. Kallisto differential analysis results were used to perform Gene set Ontology Analysis. For this, we generated a ranked list calculating a composed score (=Log_2(Fold Change[G34R/Wt])/qValue) for each gene in mouse and human cells. These ranked lists were used for GSEA using the GSEA v4.1.0 software (Broad Institute, Inc.). Gene

ontologies were filtered by False Discovery Rate generated by the GSEA software, for FDR < 0.05. Gene ontologies from the GSEA analysis were used to generate a gene ontology network using Cytoscape v3.7.1 [2] with the Enrichment Map plugin [3].

S2. Analysis of G34R pHGG from patient databases

To analyze the expression of genes belonging to the GO "DNA repair" and the copy number alterations and point mutations in H3.3-G34R/V pHGG patients, we used the PedcBioPortal database [4, 5]. Patient samples were selected from a pool of studies described in Table S4. Patient samples were selected by age (5-40 years), tumor location (cortical), and glioma grade (grades III/IV); only samples with specified mutation status on ATRX and P53 were included. The control group consisted of pHGG ATRX mutant, P53 mutant, and H3.3-Wt, whereas the G34R group consisted of ATRX mutant, P53 mutant, and H3.3-G34R/V. Within these groups, we interrogated for mutation counts and copy number alterations in the samples whose genomes were sequenced and for the normalized expression values of DNA repair GO genes expression on the samples where RNA-seq data was available. Z-scores were used to compare RNA expression values among different studies. The expression of each DNA repair gene within the Wt and G34R groups was compared using unpaired t-tests on the whole dataset which allowed to calculate the false discovery rate (FDR) values for each gene. The frequency of copy number alterations and mutations in G34R and Wt groups were compared with Two-way ANOVA analysis using GraphPad Prism 7.

S3. Immunohistochemistry (IHC) of paraffin-embedded brains

Following perfusion, mouse brains were fixed in 4% PFA (paraformaldehyde) for an additional 48 hours at 4°C, then transferred to 70% ethanol, and processed and embedded in paraffin at the University of Michigan Microscopy & Image Analysis Core Facility using a Leica ASP 300 paraffin tissue processor/Tissue-Tek paraffin tissue embedding station (Leica). Tissue was sectioned using a rotary microtome (Leica) set to 5 μm in the z-direction. Antigen retrieval and immunohistochemistry (IHC) of paraffin-embedded sections was performed using antibodies and dilutions listed in Table S1. Images were obtained using brightfield/epifluorescence (Zeiss Axioplan2, Carl Zeiss MicroImaging) or laser scanning confocal microscopy (Leica DMIRE2, Leica Microsystems) and analyzed using LSM5 software (Carl Zeiss MicroImaging). Immunostaining was performed using the Discovery XT processor (Ventana Medical Systems). Tissue sections were blocked for 30 minutes in 10% normal goat serum and 2% BSA in PBS. Sections were incubated for five hours with 0.1 μg/mL of rabbit polyclonal anti-G34R (Revmab, 31-1120-00-S) or anti-ATRX (Abcam, ab97508) antibodies. Tissue sections were then incubated for 60 minutes with biotinylated goat anti-rabbit IgG (Vector Laboratories, PK-6101) at 1:200 dilution. Blocker D, Streptavidin-HRP, and DAB detection kit (Ventana Medical Systems, 760-124) were used according to the manufacturer’s instructions. For automated scoring, each slide was scanned using an Aperio Scanscope Scanner (Aperio) and viewed through Aperio ImageScope software. A blinded individual (M.H) captured three randomly selected TIFF images from each slide at 10 X magnification on the Aperio ImageScope viewing program. Quantification of immunostaining on each image was conducted using ImageJ. The algorithm used color

segmentation with RGB color differentiation, K-Means Clustering, and background foreground separation with Otsu's thresholding. To arrive at a score for each marker, the number of extracted pixels was multiplied by their average intensity for each core.

S4. Limited dilution assay

H3.3-G34R and H3.3-Wt human pHGG cells were plated in 96-well plates at 1, 2, 4, 8, 16, 32, 64 and 128 cells/well/200 µL of the corresponding media (see methods 2 and 3). The number of wells containing live cells was evaluated after one week. LDA assay analysis was performed with software available at http://bioinf.wehi.edu.au/software/elda/ (44).

S5. Evaluation of Tumor Initiating Cell (TIC) frequency *in vivo*

To determine the tumor initiation capacity of H3.3-Wt and H3.3-G34R pHGG cells, 6- to 8-week-old C57BL/6J mice were stereotactically injected with 30, 100, 300, 1,000, 3,000, 10,000, 30,000 and 100,000 cells in the striatum in the following coordinates from bregma: 1.0 mm anterior; 2.0 mm lateral, and 3.0 mm deep). When the mice became symptomatic (lethargic, scruffy coat, hunched posture) due to tumor burden, they were anesthetized using 120 mg/kg of ketamine combined with 0.5 mg/kg of dexmedetomidine and then perfused with 4% PFA.

S6. Clonogenic assays

Mouse and human H3.3-G34R and H3.3-Wt pHGG cells were seeded into T25-well plates at a density of 10 × 10^6 cells per flask in DMEM:F12 (1:1) medium supplemented with 20% fetal bovine serum (Biowest, Cat. # 1520) (for mouse cells, 20% FBS was used instead of the defined media with B27 and N2 supplements to induce cell adherence to the flask surface), or in IMDM (Gibco, Cat # 12440061) supplemented with 20% FBS (Biowest, Cat. # 1520), and penicillin-streptomycin (100 U/mL) for human cells. After being incubated for 24 hours, the cells were treated with different doses of IR (mouse cells: 0, 2, 3, and 5 Gy; human cells: 0, 2, 5, and 10 Gy). Immediately after irradiation, the cells were detached, serially diluted, and seeded (cell numbers ranging from 100 to 100,000 cells per well depending on cell line and radiation dose) in triplicates into 6-well plates and then placed in an incubator at 37°C set at 5% CO2 for ten days to allow colony formation. The plates were then washed with PBS, and each well was fixed with glutaraldehyde (6.0% v/v), with crystal violet (0.5% w/v) to stain [6]. After washing and drying the plates, stained colonies with a cell number >50 were counted using a bright field microscope. The survival fraction was calculated relative to non-irradiated cells.

The same procedure was followed to assess the response of H3.3-G34R/Wt pHGG cells to temozolomide (TMZ). In that case, cells were irradiated, plated, and TMZ was added at the concentrations indicated in the figure (0;0.002;0.02 and 0.2 M TMZ).

The results were all normalized to non-treated controls, to subtract the efficiency of plating of each cell from the experiment, and only assess the differences owed to the irradiation. 200 non irradiated cells were plated, and the plating efficiency factor was calculated as [#colonies obtained/#cells plated] in the non-treated plates for each cell type. Cells were irradiated or TMZ added, counted and plated in triplicates with 3 different plating cell

numbers. The survival fraction for each irradiation dose was calculated as [plating efficiency]/[colonies obtained case plate/cells plated case plate] for each treated plate.

S7. Assessment of DNA repair Activity *in vitro*

A previously published DNA repair activity assay based on reporter plasmid was used [7]. This system allows to measure homologous recombination (HR) and non-homologous end joining (NHEJ) DNA repair activities independently [7-9]. These systems are designed to express enhanced green fluorescent protein (EGFP) when the plasmid is repaired via HR or NHEJ, respectively. HR and NHEJ plasmids are exhaustively linearized by digestion with I-*Sce*I endonuclease and transfected into cells. When the DNA repair machinery repair the linear break via the corresponding DNA repair mechanism (HR or NHEJ, respectively, for HR and NHEJ plasmids), EGFP expression is reconstituted. Ten µg of HR and NHEJ plasmids were linearized by I-*Sce*I digestion (50 U, New England BioLabs, R0649L) and purified on a 0.6% agarose Gel. The linearized digestion product band were excised from the gel and purified with QIAprep Spin Miniprep Kit columns (Qiagen, Cat. # 27104).

S8. Flow cytometry to assess expression of glioma stem cell markers

Cells were collected and detached into single cell suspensions via Accutase (Biolegend, Cat. # 423201) treatment, counted, and distributed in $1x10^5$ cells per well in a 96 well plate. Cells were washed with PBS and Fc-blocked with CD16/CD32 human/mouse antibodies for 15 minutes in ice. Cells were washed with PBS and stained with mouse CD44-Alexa Fluor 700 (Biolegend, Cat #103026) 1:200 and CD133-Cy7 (Biolegend, Cat

141209) 1:200 (mouse cells) or human CD44-FITC (Biolegend 103005) 1:200 and CD133-Cy7 (Biolegend, Cat # 141209) 1:200 (for human cells) and incubated for 30 minutes on ice, in dark. After that, cells were fixed and permeabilized using the Foxp3 Transcription Factor Staining Buffer Set (ThermoFisher, Cat. # 00-5523-00), according to the manufacturer instructions, and ALDH-PE/Alexa Fluor 700 (Abcam, Cat # ab209437 or R&D Systems, AAF5869-SP and conjugated after with anti-Goat Alexa Fluor 680, ThermoFisher Cat. # A-21084) antibody was added. Cells were washed and resuspended in flow cytometry buffer and run in a BD FACS-Aria II flow cytometer.

S9. *In vitro* and *in vivo* DNA-replicating cell frequency and mitotic rate analysis via flow cytometry

EdU was added to in vitro cultured cells at a final concentration of 10 μM. Mouse cells were incubated for 2 hours in presence of Edu, and human cells were incubated for 3 hours. After Edu incubation, cells were collected and detached into single cell suspensions via Accutase (Biolegend, Cat. # 423201) treatment, counted, and distributed in $1x10^5$ cells per well in a 96 well plate. Edu was stained using Click-iT™ EdU Cell Proliferation Kit for Imaging a Click reaction according to the manufacturer's protocol, and EDU-stained cells were run in a BD FACS-Aria II flow cytometer.

Mice harboring H3.3-Wt or H3.3-G34R SB tumors were injected intraperitoneally with 10 mg/kg EdU three hours before being euthanized. Tumors were dissociated into single cell suspensions and CD45 cells were labeled with magnetic beads (Miltenyi Biotec, Catalog # 130-052-301) following the manufacturer's protocol. After 15 minutes of incubation at 4°C, cells were washed and passed through a preconditioned MS column placed in the

magnetic field of a MACS Separator. The unlabeled (CD45 negative) cells derived from dissociated tumors were collected and resuspended in PBS containing 2% FBS for flow cytometry analysis. To examine the cell cycle properties of the tumors, cells were fixed, permeabilized, and stained for pH3 (Ser10) (1: 50, Thermofisher, PA5-17869) and a click reaction was performed to detect EdU following the manufacturer's instructions (C10418, Thermofisher). Stained cells were run in a BD FACS-Aria II flow cytometer.

S10. Immunofluorescence (IF) of *in vitro*-cultured cells,

For adherent cells (human SJGBM-derived pHGG cells), we placed sterilized round-shaped coverslips into 24-well plates (1 coverslip per well), and we seeded $2x10^4$ cells per well in 0.5 mL of media. For floating cells (mouse primary pHGG cells), $2x10^5$ cells per well in 6-well plates in two mL of media were plated. One day after plating, cells were irradiated (IR, 3 Gy), and collected at the indicated time post-IR. At the time of collection, for adherent cells, cells were washed twice with PBS and 300-400 µL of buffered 3% formaldehyde fixative solution were added to each well and cells were incubated at room temperature for 20 minutes. For floating cells, the media with cells was collected and loaded into a cytospin cassette and were centrifuged at 500 RPM for ten minutes in Cytocentrifuge (Thermo, Cytospin 4) to concentrate the cells in a slide. Afterwards, slides were washed twice with PBS and buffered 3% Formaldehyde Fixative solution was added on top of each slide and then incubated at room temperature for 20 minutes. Following fixation, the subsequent steps of the protocol are the same for adherent and floating cells. Wells/slides were washed twice with PBS and, after, washed twice with 400 µL of wash buffer (0.1% BSA in 1X PBS). Next, cells wells/slides were blocked for non-specific

staining with 400 µL of blocking buffer (10% normal horse serum, 0.3% Triton X-100) for 45 minutes at room temperature. Following blocking, samples were incubated with primary antibody prepared in dilution buffer (1% BSA, 1% normal horse serum, 0.3% Triton) for one hour at room temperature. Wells/slides were washed twice with 400 µL of wash buffer (0.1% BSA in 1X PBS), and incubated with fluorescent-conjugated secondary antibodies, for one hour at room temperature in dark. After, wells/slides were washed twice with 400 µL of wash buffer (0.1% BSA in 1X PBS) and stained with 300 µL DAPI solution (1:5000 of a 14.3 mM DAPI stock solution) for two minutes, rinsed with PBS and mounted with ProLong™ Gold Antifade Mountant (ThermoFisher, Cat. # P36930). Slides were imaged after 24 hours using an Olympus BX53 microscope and cellSens Dimension software (Olympus).

S11. Confocal microscopy and image analysis

Cell were placed into the incubator chamber of the microscope at 37°C with a 5% CO2 atmosphere. Microscopy confocal images were acquired using a single photon laser scanning inverted confocal microscope LSM 880 AxioObserver (Carl Zeiss, Jena, Germany). Two laser lines were used for simultaneous excitation with Plan-apochromat 63 x/1.4 numerical aperture (NA) oil DIC M27 objective. Cells stained with Hoechst33342 were excited at 361 nm and 497 nm wavelength respectively. Line-sequential scan mode and 1.4 scan zoom were used. The system was driven by ZEN Black software. Multichannel immunofluorescence images were processed and analyzed using Fiji (ImageJ2). Micronuclei were manually identified and quantified using the plugging Cell Counter.

S12. Western Blotting

For each experimental point, mouse and human H3.3-Wt and H3.3-G34R pHGG cells were seeded at density of 3.0 x 10^6 cells into one 75-cm^2. After 24 hours, cells were treated with IR (3 Gy). Cell lysates were generated by collecting cells at different time points post IR by incubating glioma cells and 0.2 mL RIPA lysis buffer (ThermoFisher, Catalog # 89900) containing protease inhibitors (ThermoFisher, Halt™ Protease and Phosphatase Inhibitor Cocktail (100X), Catalog # PI78440) on ice for five minutes. Resulting cell lysates were centrifuged at (13,000 RPM, 10', 4°C) and supernatants were collected. Protein concertation was determined via bicinchoninic acid assay (BCA) (Assay Kit Pierce BCA, Cat # PI23227, Thermo Scientific). For electrophoretic separation of proteins, 20 µg of total protein were resuspended in loading buffer (10% sodium dodecyl sulfate Sigma Aldrich 71736, 20% glycerol, and 0.1% bromophenol blue) incubated five minutes at 95°C and loaded onto a 4-12% Bis-Tris gel. Proteins from the gel were transferred to 0.2 µm nitrocellulose membrane and blocked with 5% nonfat milk in TBS-0.1% Tween-20. After blocking, membranes were incubated with primary antibodies overnight at 4°C. The next day, blots were washed with TBS-0.1% Tween-20 and incubated with secondary (1:4000) antibody for one hour at room temperature. Blots were washed several times again with TBS-0.1% tween-20 and visualized under Biorad gel imaging software. Band intensities were quantified using GelAnalyzer software. For Western blots involving histone marks, 5x10^7 cells were processed for histone extraction with the Histone Extraction Kit from Active Motif (Catalog # 40028) following the manufacturer protocol. 10 µg of histone extract were used per sample for each Western

blot experiment. A list of the antibodies used for WB and other experiments is provided in Table S8.

In the case of the DNA repair WB experiments, all the WB represented in Figure 6 (DNA damage response proteins) were performed using the same protein extracts. Cells were irradiated, and upon protein extraction, extracts were quantified via BCA assay, and the concentrations were normalized to 1.5 µg/µl, aliquoted into 15 µl aliquots and stored at -80oC. WB was performed for each DNA repair protein using an aliquot of each protein extract (corresponding to a time post-IR) per WB. For each WB, corresponding to a different DNA repair phospho-mark or total protein, a loading control protein was used, which was either actin or vinculin, depending on the molecular weight of the DNA repair protein developed. As the protein extracts used throughout all the WB in these experiments were the same for each experimental time point and cell type, we included only one representative Actin loading control in the figure.

S13. Micrococcal nuclease digestion assay

For the micrococcal nuclease digestion assay, nuclei were prepared by resuspending cell pellets in Nuclear Buffer A (85 mM KCl, 10 mM Tris–HCl, 0.2 mM spermidine, 0.2 mM EDTA, 160 mM sucrose, 250 µM phenylmethylsulfonyl fluoride) and then adding five mL of Nuclear Buffer B (Nuclear Buffer A supplemented with NP-40 0.1% vol/vol), mixing by inversion, and incubating ten minutes on ice to ensure complete lysis. Nuclei were collected via centrifugation (1,300 g, 5’, 4°C) and then resuspended in five mL Mnase digestion buffer (50 mM Tris-HCl: pH 8.0, 1 mM CaCl2, 0.2 % Triton X-100). To remove cellular debris, nuclei were passed through 7.5 mL of chilled sucrose cushion (10 mM HEPES: pH 7.9, 30% (w/v) sucrose, 1.5 mM MgCl2) and collected via centrifugation

(1300 g, 12', 4°C, swinging bucket tabletop). The homogenized nuclei were re-suspended in 600 µL of Mnase digestion buffer, and the absorbance at 260 nm was recorded using a nanodrop (NanoDrop OneC, Thermo). The concentration of nuclear DNA was adjusted to four absorbance units using MNase digestion buffer. Then the DNA extracts were divided in six microcentrifuge tubes, and 50 U/mL Mnase (Worthington, 9013-53-0) was added to each tube. The reaction was stopped 1, 2, 4, 8, 16, or 32 minutes after the addition of Mnase by using 300 µL of Mnase Stop Buffer (2% SDS, 200 µg/mL proteinase K, and 10 mM EDTA). Purified DNA was fractionated on a 1% agarose gel in the presence of an intercalating dye (SYBR Safe). Gel images were analyzed using GelAnalyzer 19.1 (www.gelanalyzer.com) by Istvan Lazar Jr., PhD and Istvan Lazar Sr., PhD, CSc, and the percentage of mononucleosomes was calculated using densitometry analysis.

S14. Chromatin relaxation to assess DNA repair activity in human and mouse pHGG cells

To assess the effect of chromatin relaxation in DNA repair kinetics, $1x10^6$ mouse or human cells were seeded in 25 cm^2 tissue culture flasks (eight flasks per experiment per cell type). Chromatin relaxation was induced by subjecting the cells to hypotonic conditions, as previously reported [10, 11]. Six flasks were irradiated (five Gy ionizing radiation, IR), and immediately after irradiation, half were subjected to hypotonic conditions, by diluting the osmolarity to 75 mM with one volume of ultrapure sterile H2O. The remaining half were maintained in isotonic conditions, by dilution with one volume of sterile 150 mM NaCl solution. One flask of cells per treatment condition (*I.e.,* hypotonic or isotonic conditions) was collected per each time post-IR time point assessed. Non-

irradiated flasks were subjected to either hypotonic or isotonic conditions for 24 hours and collected as non-IR controls. Floating mouse cells were collected by centrifugation and adherent human cells were collected by scraping and washing with PBS (ThermoFisher, Catalog #10010-023). Cell pellets were resuspended in 100 µL of RIPA buffer (ThermoFisher, Catalog # 89900) containing protease and phosphatase inhibitors and processed for Western blot as detailed in method S12).

S15. *In vitro* Dose-Response evaluation of DNA damage response inhibitors and to temozolomide (TMZ)

We assessed the susceptibility of H3.3-G34R and H3.3-Wt mouse and human cells to DDRi. We evaluated a novel PARP inhibitor, Pamiparib (BGB-290) and a cell cycle checkpoint (Chk1/2) inhibitor. We plated $2x10^3$ mouse or human cells per well on 96-well plates in 190 µL of media. We used five wells per inhibitor dose evaluated for each cell type. We evaluated seven concentrations of inhibitor in serial logarithmic dilutions (e.g., 1 µM, 3 µM, 10 µM, 30 µM, 100 µM and 300 µM). One day after plating the cells 10 µL of 20X concentrated inhibitor was added to each well for each dilution evaluated. Cells were incubated for three days, and viability was assessed using CellTiter Glo assay (Promega, Cat. # G7572). Additionally, we assessed cell survival combining DDRi with IR (two Gy). In those cases, cells were irradiated one hour before the addition of the DDRi. The results were evaluated with GraphPad Prism 7 using a sigmoidal regression model, which allows the IC_{50} calculation and the evaluation of the statistical differences of the curves between cells. In the case of the experiment comparing endogenous histone-Wt and histone-

mutant pHGG primary human cells (Figure S19, C-D), SJ-GBM2 cells (H3.3-Wt endogenous) and OPBG-GBM-001 (H3.3-G34R) cells were used.

S16. Neutral Comet Assay

Neutral Comet assay [12] was performed on mouse and human pHGG cells according to the protocol detailed in [13]. Cell were seeded at a density of 3.0 x 10^6 cells into five mL of media in a T25 flask and subjected to three Gy of IR. Cells were collected four hours after radiation, and 2x10^5 cells were embedded in 200 µL of 0.7% low melting Agarose ant 38°C and plated over 0.8% low melting point agarose (Sigma-Aldrich, Cat. # A9414) pre-coated microscope slides and covered with coverslips. Agarose was allowed to solidify at 4°C for 15 minutes, then topped with an agarose layer of (80 µL 0.8 %, 38°C), and slides were covered with coverslips again. After solidification of the top agarose layer, coverslips were removed, and slides were placed on Neutral Comet lysis solution overnight at 4°C. On the following day, slides were washed and subjected to electrophoresis (0.5 V/cm during 1 h). Each slide was developed by adding 100 µL of SYBR Safe DNA Gel Stain (ThermoFisher, Cat. # S33102), diluted 1:1000, and covered with coverslip for imaging. We counted 5 cells per microscope field, and we used 4 fields belonging to independent experimental replicates.

S17. Posttranslational modifications Phospho-Array

To assess the levels of posttranslational activation of DNA repair and cell cycle proteins, we used an antibody array (Cell Cycle Control Phospho Antibody Array, Cat. # PCC238

FullMoon BioSystems) encompassing 238 site-specific and phospho-specific antibodies involved in cell cycle regulation and DNA damage response/repair mechanisms. Protein was extracted from $2x10^6$ cells for each cell type (Human and Mouse H3.3-Wt and H3.3G34R pHGG cells, respectively), following the protocol from Antibody Array Assay Kit (FullMoon BioSystems, Cat. # KAS02). After purification, proteins were quantified by BCA assay (Assay Kit Pierce BCA, Cat # PI23227, Thermo Scientific) according to the manufacturer instructions, and 100 µg of protein per cell type were biotinylated according to the protocol from Antibody Array Assay Kit (FullMoon BioSystems, Cat. # KAS02). Antibody array slides were blocked, and biotinylated proteins were coupled to the arrays following (Cell Cycle Control Phospho Antibody Array, Cat # PCC238 FullMoon BioSystems) instructions. After protein coupling and washing, the biotinylated proteins that bound the antibodies spots on the array were stained with Cy3-streptavidin according to the manufacturer instructions, and the arrays were developed with a Genepix 4100A Microarray Scanner (UMICH Single Cell Analysis Resource Core). Images were analyzed using **ImageJ** software [14, 15], and spots were quantified into intensity values using the Plugin **Protein Array Analyzer** for ImageJ [16]. Each value was normalized by dividing by the Actin average intensity, and each phospho-specific mark was normalized to each corresponding total antibody average intensity. As the array provides six spots for each site-specific and phospho-specific antibody, the analysis resulted in six normalized intensity values per protein-specific and phospho-specific mark. The values were analyzed in GraphPad Prism 7, and Unpaired T-tests were performed to compare individual protein and phospo-site intensity between H3.3-G34R and H3.3-Wt, providing Significance Statistical analyses (False discovery rates) and Fold changes for each

protein and phospo-site mark between H3.3-G34R and H3.3-Wt mouse and human cells, respectively. The NES (normalized enrichment score) was determined using the Gene Ontology Set Analysis (GSEA) software from the Broad Institute (https://doi.org/10.1073/pnas.0506580102). The NES represents the deviation of the genes/mark on the signature toward being upregulated or downregulated, and an NES of 0 indicates that the genes/mark belonging to the signature are neither upregulated nor downregulated in comparison with the average of all the genes/marks. A negative NES indicates downregulation of the signature.

S18. Determination of micronuclei frequency

To access the micronuclei, cells (1 x 10^5) were placed on glass chambered coverslip and incubated overnight. Following incubation within the optimum growth condition, half of the cells were irradiated (3 Gy IR) while the rest were left as non-IR samples. After 72 hours the cells were harvested, and DNA was labelled with Hoechst 33342 dye 5 μM (ThermoFisher Catalog # H3570) for 20 minutes. Cells were subsequently rinsed with HBSS and placed in fresh growth medium before analysis through confocal microscopy. Micronuclei were defined as discrete DNA aggregates separated from the primary nucleus in cells where interphase primary nuclear morphology was normal. Cells with an apoptotic appearance were excluded.

S19. Determination of Type I Interferon levels and DAMPs

To evaluate the levels of type I interferons and DAMPs, mouse and human pHGG cells (1×10^6) were seeded into 6-well plates and allowed to settle overnight before treatment. On the next day, cells were treated with STING inhibitors: H151 (1 μM) and STING-dependent IRF3 activation inhibitor, GSK690693 (1 μM), JSH23, NF-κB activation inhibitor; and PDTC, NF-κB inhibitor, for two hours prior to receiving three Gy of irradiation. After 72 hours, the levels of IFN-β, HMGB1 and ATP in the cultures' supernatants were measured by ELISA according to manufacturer's instructions (R&D) at the Cancer Center Immunology Core, University of Michigan.

S20. Transplantable pHGG model

Injections were performed using a stereotactic fixation device (Stoelting). Mice were first anesthetized with intraperitoneal injections of ketamine (0.1 mg/g) and xylazine (0.01 mg/g). A local injection of bupivacaine was used as analgesic. One microliter of 5×10^4 cells in suspension was delivered using a 30-gauge needle attached to a Hamilton syringe. The location of injection, AP 1.7 mm from bregma, Lat 0.5 mm, and depth 3.5 mm from the dural surface, was determined according to the coordinates in the mouse brain atlas to target the right frontal striatum. Buprenorphine was administered to mice for pain relief post-injection. Mice were continually monitored and euthanized when at onset of visible symptoms of tumor burden (*I.e*, lethargy, scruffy coat, hunched posture).

S21. ***In vivo*** **Radio sensitivity study**

Male and female C57BL/6 mice (n = 24 total), aged 6-8 weeks, were implanted with 5 x 10^4 H3.3-Wt or H3.3-G34R cells (n = 12 each). We followed an adaptation of RT for mouse that has been previously optimized for maximum efficacy, and described in Leder et al., 2014, [17]. In this study, the authors we adopted a combined experimental and theoretical approach to identify treatment schedules that would lead to better survival in animal models of glioma, taking in account the dynamic transitions of cells between relatively radiosensitive and radioresistant pools. At 7 days post-implantation, mice with each tumor genotype were randomly assigned to: (i) 20 Gy of ionizing radiation (IR), or (ii) controls without radiation treatment. Sample size was n = 6 for each genotype/treatment group. Tumor growth was monitored three times per week after treatment up to a moribund state, at which point the animals were euthanized and included in the survival curve.

S22. H3.3-G34R pHGG Rechallenge

Mice implanted with H3.3-G34R pHGG mouse cells that survived with the IR or IR + DDRi treatments were rechallenged to assess the development of antitumor immunological memory. Six mice were rechallenged on the contralateral hemisphere (Coordinates of injection, AP 1.7 mm from bregma, Lat 0.5 mm, and depth 3.5 mm from the dural surface) with $5x10^4$ G34R pHGG mouse cells. Tumor growth was monitored twice a week after treatment. The experiment was terminated at day 100, the point at which animals were perfused and tissues were fixed for histological analysis.

S23. Inhibition of DNA damage response *In vivo*

CHK1/2 inhibitor AZD7762 was dissolved in 0.9% saline containing 11.3% (2-hydroxypropyl)- β-cyclodextrin (Sigma Aldrich, H107-5G) on a magnetic stirrer for 30 minutes and stored at 4°C. PARP inhibitor pamiparib was dissolved in containing 10% (2-hydroxypropyl)- β-cyclodextrin (Sigma Aldrich, H107-5G) on a magnetic stirrer for 30 minutes and stored at 4°C. Veliparib was prepared in 5% DMSO in 0.9% saline and pH was adjusted to 5.5.

Twelve days after tumor implantation, 15 mg/kg of AZD7762, 10 mg/kg of pamiparib or 25 mg/kg veliparib [18] was administered intraperitoneally to mice once a day along with two Gy radiation five days a week for two weeks. Mice were assessed for survival with the treatment, tumor growth was monitored by luminescence twice a week, and animals were euthanized when symptoms of tumor burden were identified.

S24. Pharmacological inhibition or stimulation of the cGAS-STING pathway *in vivo*

We performed pharmacological inhibition of STING using H151. H151 was prepared in PBS with 0.1% Tween-80, and 750 nmol in 200 µl of volume were administered daily intraperitoneally [19]. We performed pharmacological stimulation of the cGAS-STING pathway using the STING agonist diABZI (Selleckem, Cat. # S8796), which was resuspended in DMSO to a concentration of 1.25 µg/µl. 5 µg of diABZI were administered intracranially in 4 µl of volume on the site of the tumor implantation [20], twice at days 13 and 19 post-implantation. As vehicle control, 4 µl of DMSO were administered intracranially on the site of the tumor implantation administration (non-treated group).

References

1. Bray, N.L., et al., *Near-optimal probabilistic RNA-seq quantification.* Nat Biotechnol, 2016. **34**(5): p. 525-7.
2. Shannon, P., et al., *Cytoscape: a software environment for integrated models of biomolecular interaction networks.* Genome Res, 2003. **13**(11): p. 2498-504.
3. Merico, D., et al., *Enrichment map: a network-based method for gene-set enrichment visualization and interpretation.* PLoS One, 2010. **5**(11): p. e13984.
4. Gao, J., et al., *Integrative analysis of complex cancer genomics and clinical profiles using the cBioPortal.* Sci Signal, 2013. **6**(269): p. pl1.
5. Cerami, E., et al., *The cBio Cancer Genomics Portal: An Open Platform for Exploring Multidimensional Cancer Genomics Data.* Cancer Discovery, 2012. **2**(5): p. 401-404.
6. Franken, N.A.P., et al., *Clonogenic assay of cells in vitro.* Nature Protocols, 2006. **1**(5): p. 2315-2319.
7. Seluanov, A., Z. Mao, and V. Gorbunova, *Analysis of DNA double-strand break (DSB) repair in mammalian cells.* J Vis Exp, 2010(43).
8. Koschmann, C., et al., *ATRX loss promotes tumor growth and impairs nonhomologous end joining DNA repair in glioma.* Sci Transl Med, 2016. **8**(328): p. 328ra28.
9. Núñez, F.J., et al., *IDH1-R132H acts as a tumor suppressor in glioma via epigenetic up-regulation of the DNA damage response.* Sci Transl Med, 2019. **11**(479).
10. Murr, R., et al., *Histone acetylation by Trrap-Tip60 modulates loading of repair proteins and repair of DNA double-strand breaks.* Nat Cell Biol, 2006. **8**(1): p. 91-9.
11. Krieger, M.L., *Global chromatin changes induced by altered tonicity interferes with DNA damage response signaling and DNA double-strand break repair.* PhD Thesis Dissertation, Institute of Medical Radiation Biology, University of Duisburg-Essen, Germany, Online: https://duepublico2.uni-due.de/receive/duepublico_mods_00047701, 2018.
12. Courilleau, C., et al., *The chromatin remodeler p400 ATPase facilitates Rad51-mediated repair of DNA double-strand breaks.* J Cell Biol, 2012. **199**(7): p. 1067-81.
13. Boutet-Robinet, E., D. Trouche, and Y. Canitrot, *Neutral Comet Assay.* Bio-protocol, 2013. **3**(18): p. e915.
14. Rasband, W.S., *ImageJ. National Institutes of Health, Bethesda, Maryland, USA. http://imagej.nih.gov/ij.* (1997-2021)
15. Schneider, C.A., W.S. Rasband, and K.W. Eliceiri, *NIH Image to ImageJ: 25 years of image analysis.* Nat Methods, 2012. **9**(7): p. 671-5.
16. Carpentier, G. and E. Henault, *Protein Array Analyzer for ImageJ.* Proceedings of the ImageJ User and Developer Conference, Centre de Recherche Public Henri Tudor, ed., (ISBN 2-919941- 11-9), pp. 238-240, 2010.
17. Leder, K., et al., *Mathematical modeling of PDGF-driven glioblastoma reveals optimized radiation dosing schedules.* Cell, 2014. **156**(3): p. 603-616.
18. Owonikoko, T.K., et al., *Poly (ADP) ribose polymerase enzyme inhibitor, veliparib, potentiates chemotherapy and radiation in vitro and in vivo in small cell lung cancer.* Cancer Med, 2014. **3**(6): p. 1579-94.
19. Haag, S.M., et al., *Targeting STING with covalent small-molecule inhibitors.* Nature, 2018. **559**(7713): p. 269-273.
20. Ramanjulu, J.M., et al., *Design of amidobenzimidazole STING receptor agonists with systemic activity.* Nature, 2018. **564**(7736): p. 439-443.

Supplementary Figure 1

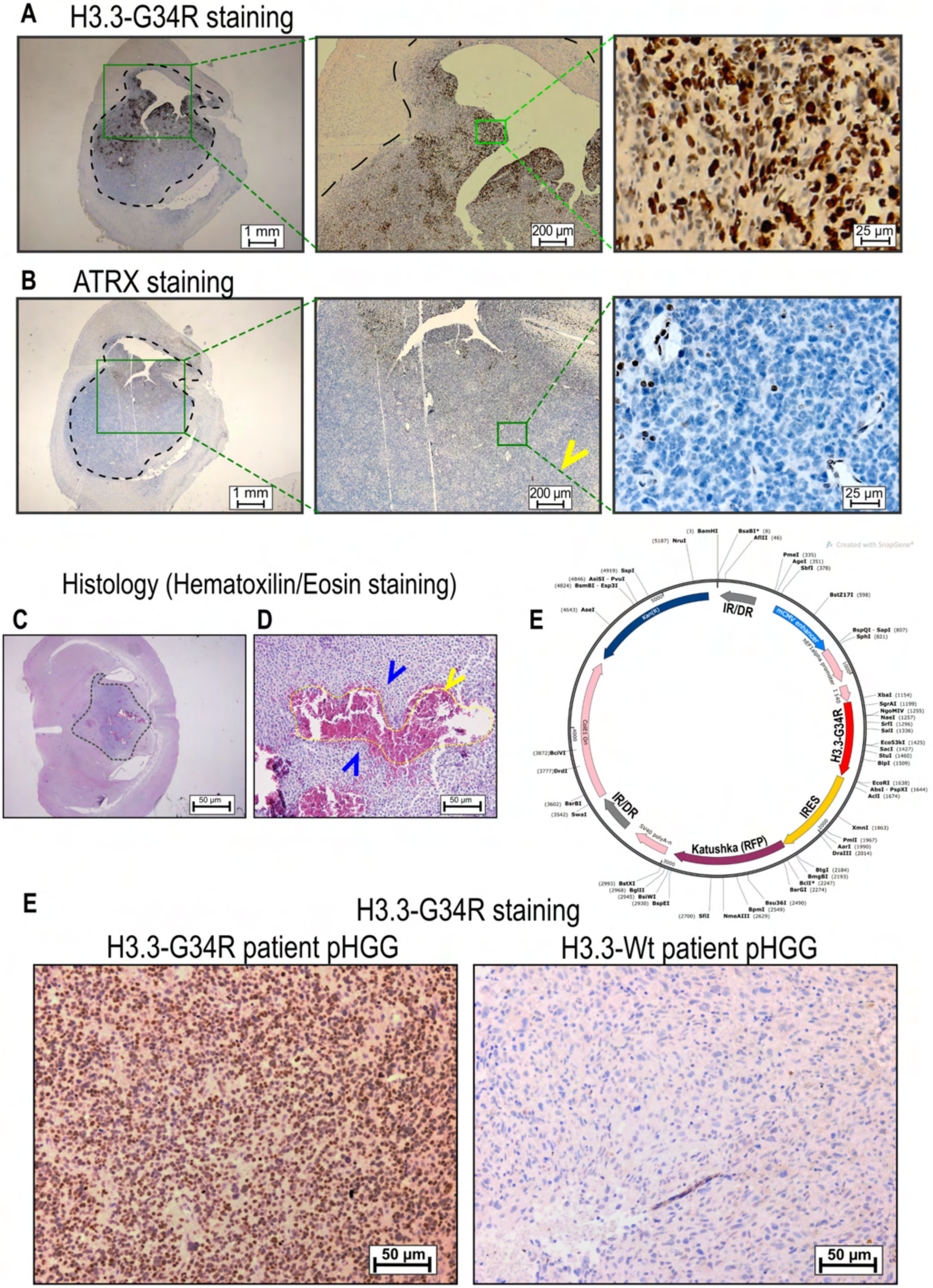

Figure S1.

A) Immunohistochemistry of a H3.3-G34R GEMM of pHGG showing the heterogeneous expression of the mutant histone in the tumor. **B)** Immunohistochemistry of a H3.3-G34R GEMM of pHGG showing the downregulation of ATRX expression in the tumor. **C-D)** Hematoxylin and Eosin staining of a *de novo* GEMM of H3.3-G34R pHGG depicting characteristics of a high-grade glioma, such as necrotic areas with hemorrhages (Yellow arrow) and palisades around the necrotic areas (Blue arrows). **E)** Map of the transposable plasmid used to integrate the H3.3-G34R expression cassette into brain cells. **F)** Immunohistochemistry of a H3.3-G34R pHGG patient-derived biopsied sample of showing the heterogenous expression of the mutant histone in the tumor (right), and comparison with a H3.3-Wt pHGG patient-derived biopsied sample (left).

Supplementary Figure 2

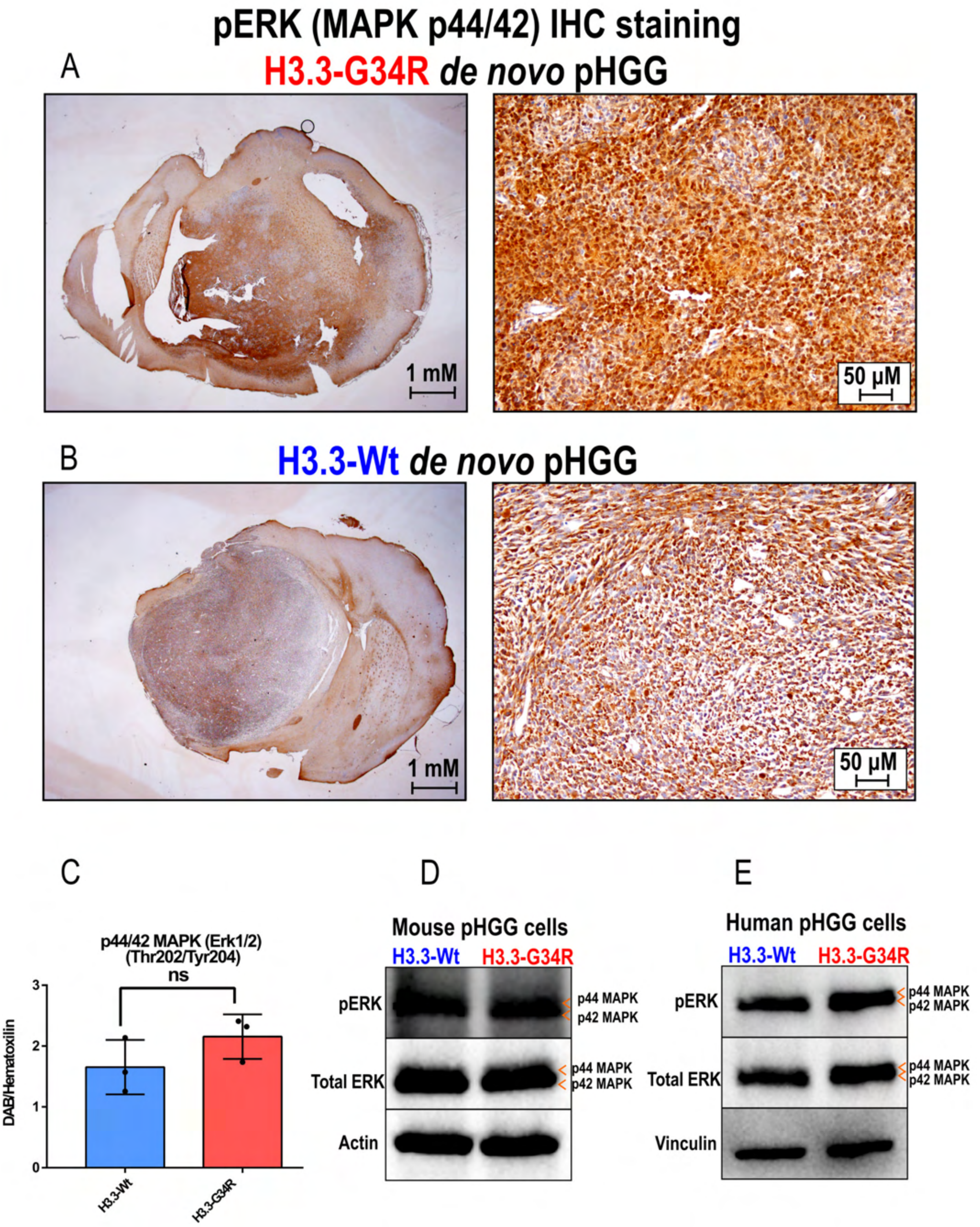

Figure S2

A-C), IHC staining of pERK in H3.3-G34R **(A)** and H3.3-Wt **(B)** pHGG de novo mouse pHGG, and quantification of the results **(C)**. **(D-E)** Evaluation of pERK levels in mouse **(D)** and human **(E)** pHGG cells through Western blotting. *(ns = non-significant; unpaired t test. Data represent mean ± SD of three different field of views)*

Supplementary Figure 3

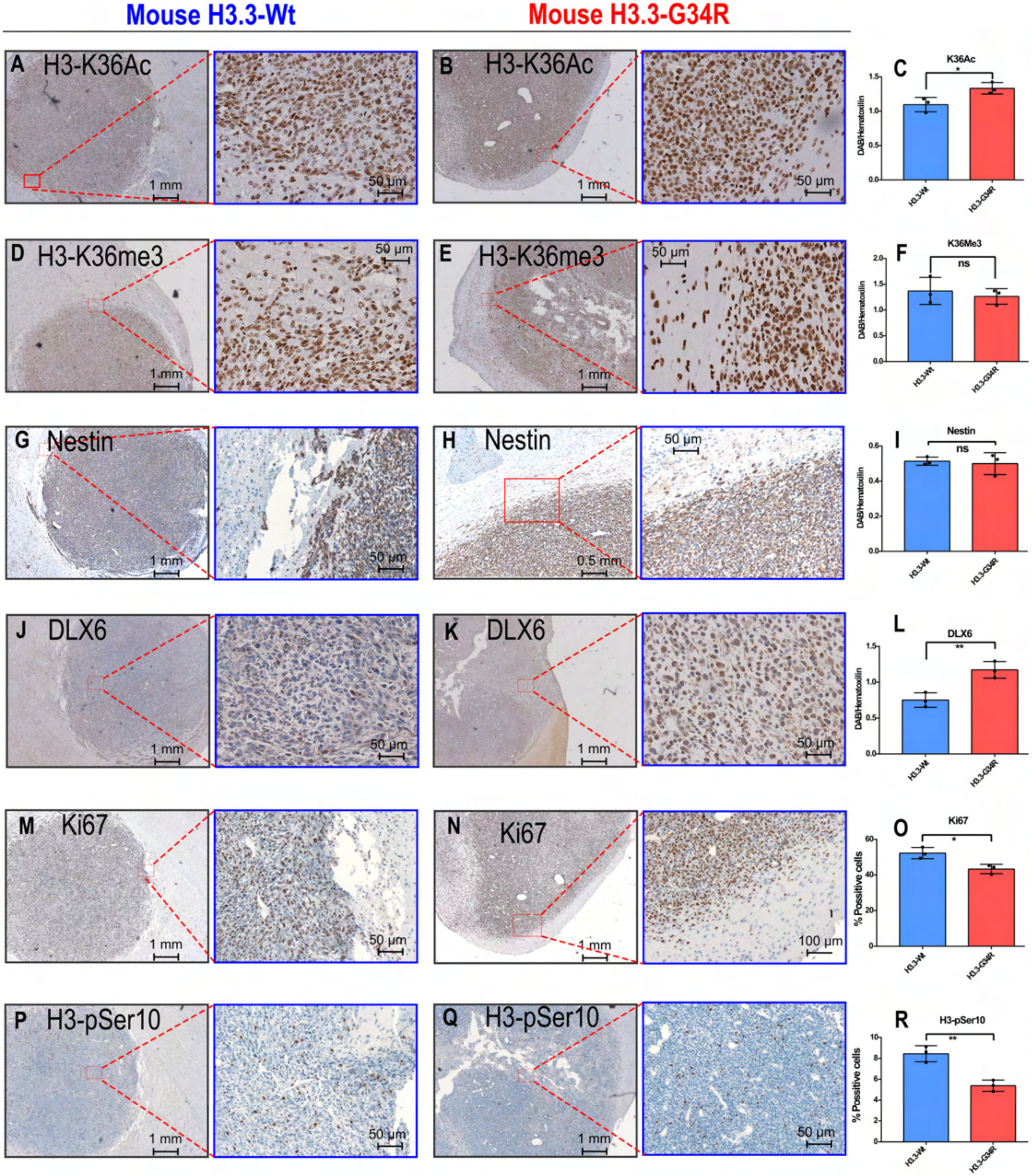

Figure S3

Immunohistochemistry of H3.3-G34R and H3.3-Wt GEMM of pHGG showing the expression and quantification of K36Ac **(A-C)**, K6me3 **(D-F)**, Nestin **(G-I)**, DLX6 **(J, L)**, KI67 **(M-O)**, and Histone3 pospho-serine10 **(P, R)**. *(*p<0.05, **p<0.01, ***p<0.005, ****p<0.001; unpaired t test. Data represent mean ± SD of three different field of views)*

Supplementary Figure 4

A

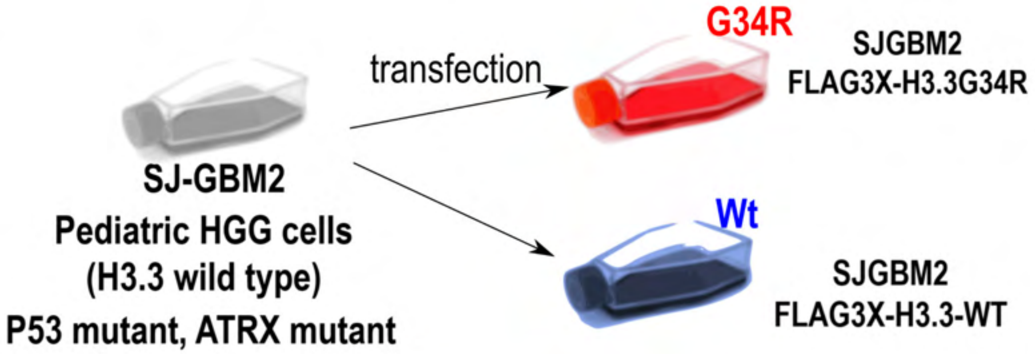

B

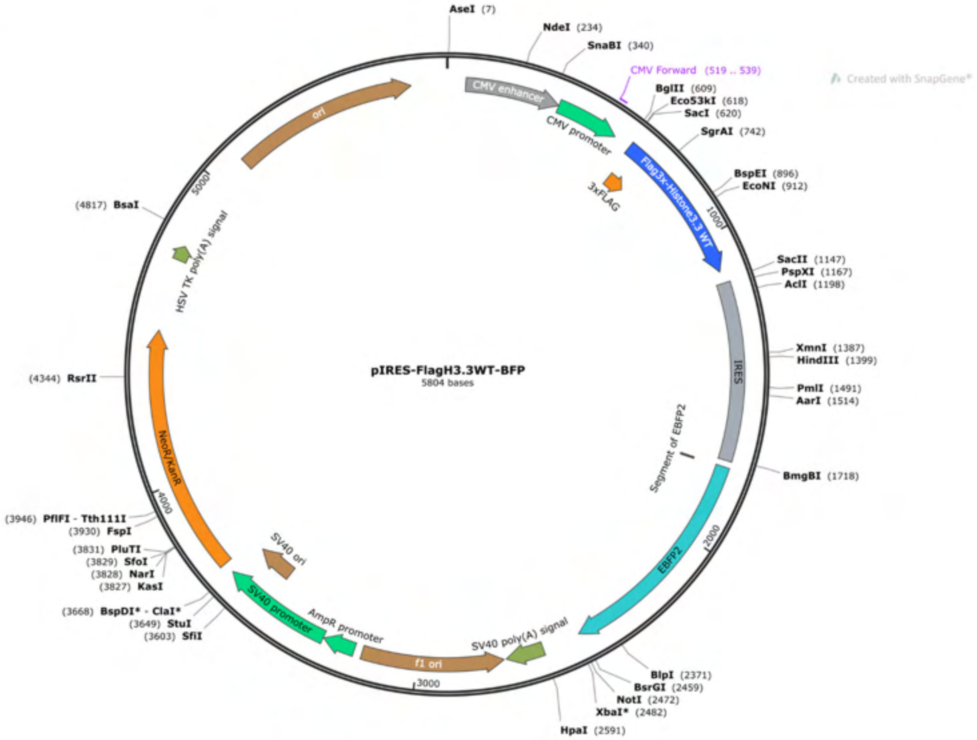

C

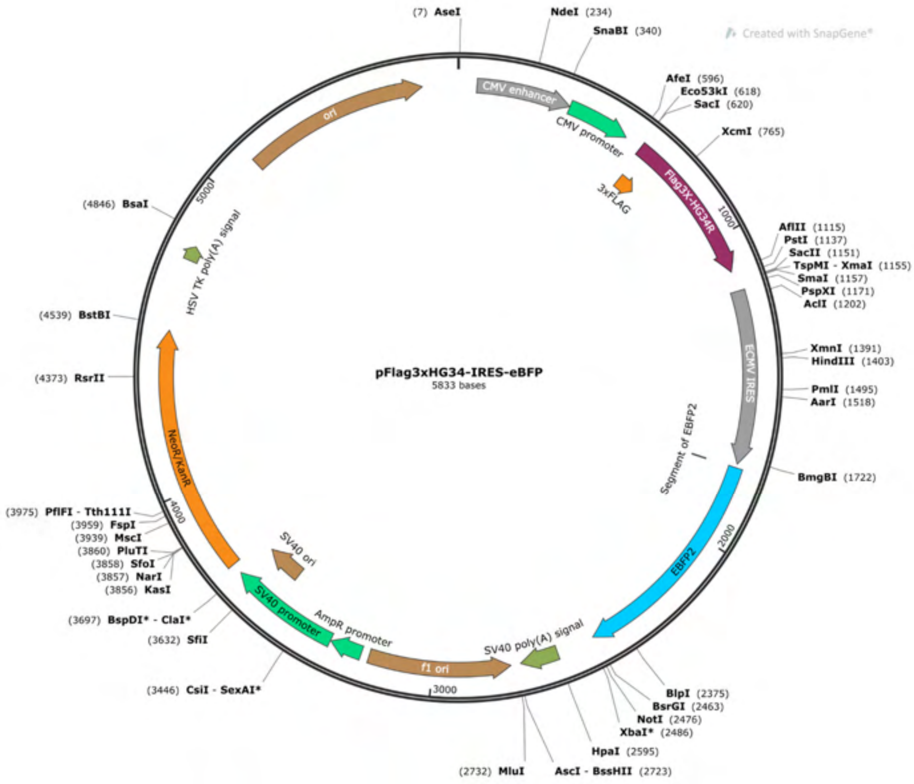

Figure S4

A) Illustration depicting the strategy followed to develop the model of human H3.3-G34R and H3.3-Wt pHGG based on the stable transfection of SJ-GBM2 cells with plasmids encoding FLAG-H3.3-G34R and FLAG-H3.3-Wt. **B-C)** Maps of the plasmids encoding FLAG-H3.3-G34R and FLAG-H3.3-Wt developed and utilized to generate the genetically engineered cells.

Supplementary Figure 5

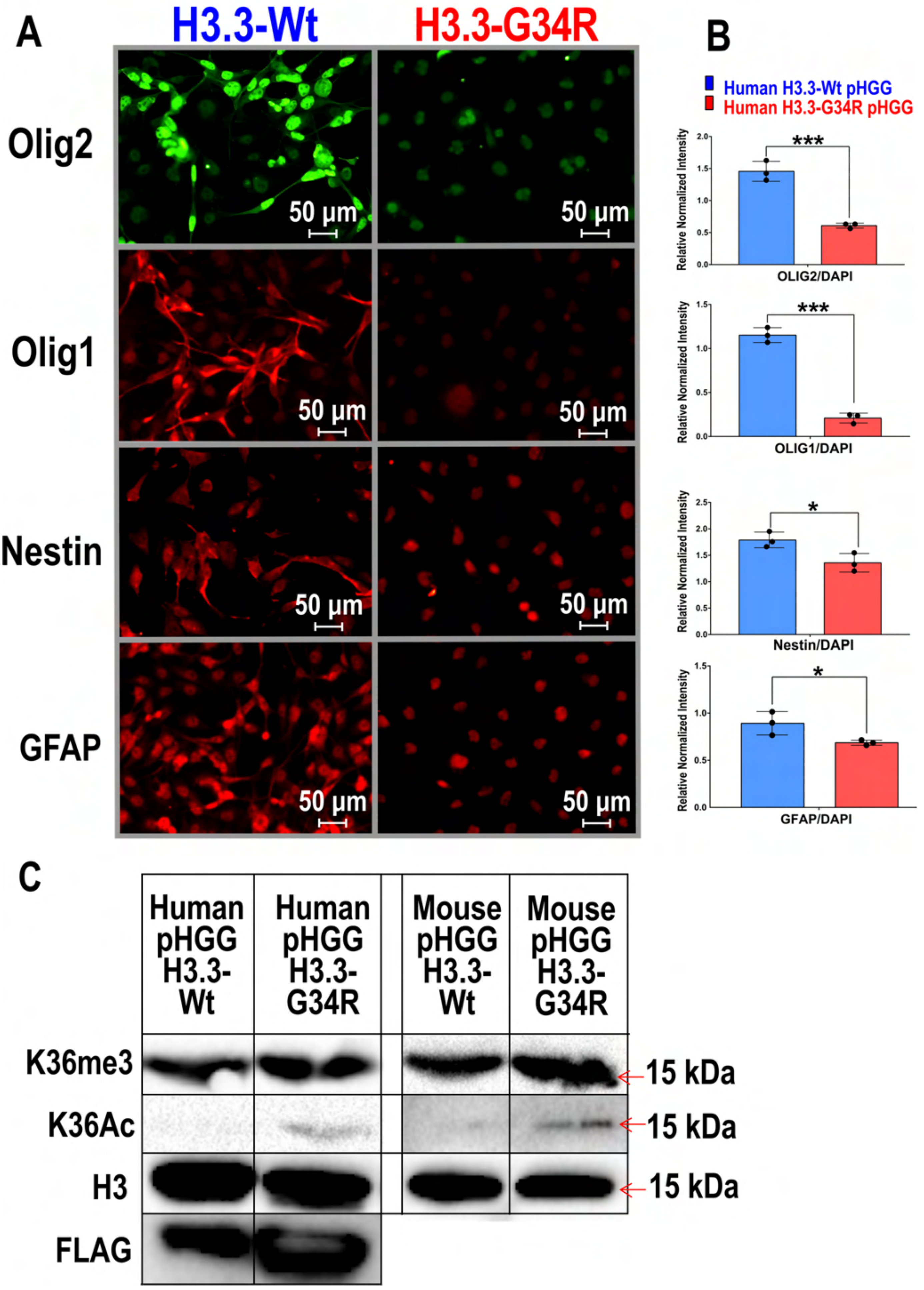

Figure S5

Characterization of the model of human H3.3-G34R and H3.3-Wt pHGG derived from SJ-GBM2 cells. A) Immunofluorescence staining for the neural markers OLIG2, OLIG1, NESTIN and GFAP, and **B)** quantification of the levels of expression based on image analysis. **C)** Western blotting for FLAG, peptide expressed as a fusion protein of H3.3-G34R and H3.3-Wt in human pHGG cells, and histone marks K36me3 and K36Ac, with total histone 3 as a histone loading control, in human and mouse pHGG cells. *(*p<0.05, **p<0.01, ***p<0.005, ****p<0.001; unpaired t test. Data represent mean ± SD of five different field of views)*

Supplementary Figure 6

Mouse pHGG: Full network of Differentially expressed Gene Ontologies (from RNA-seq)

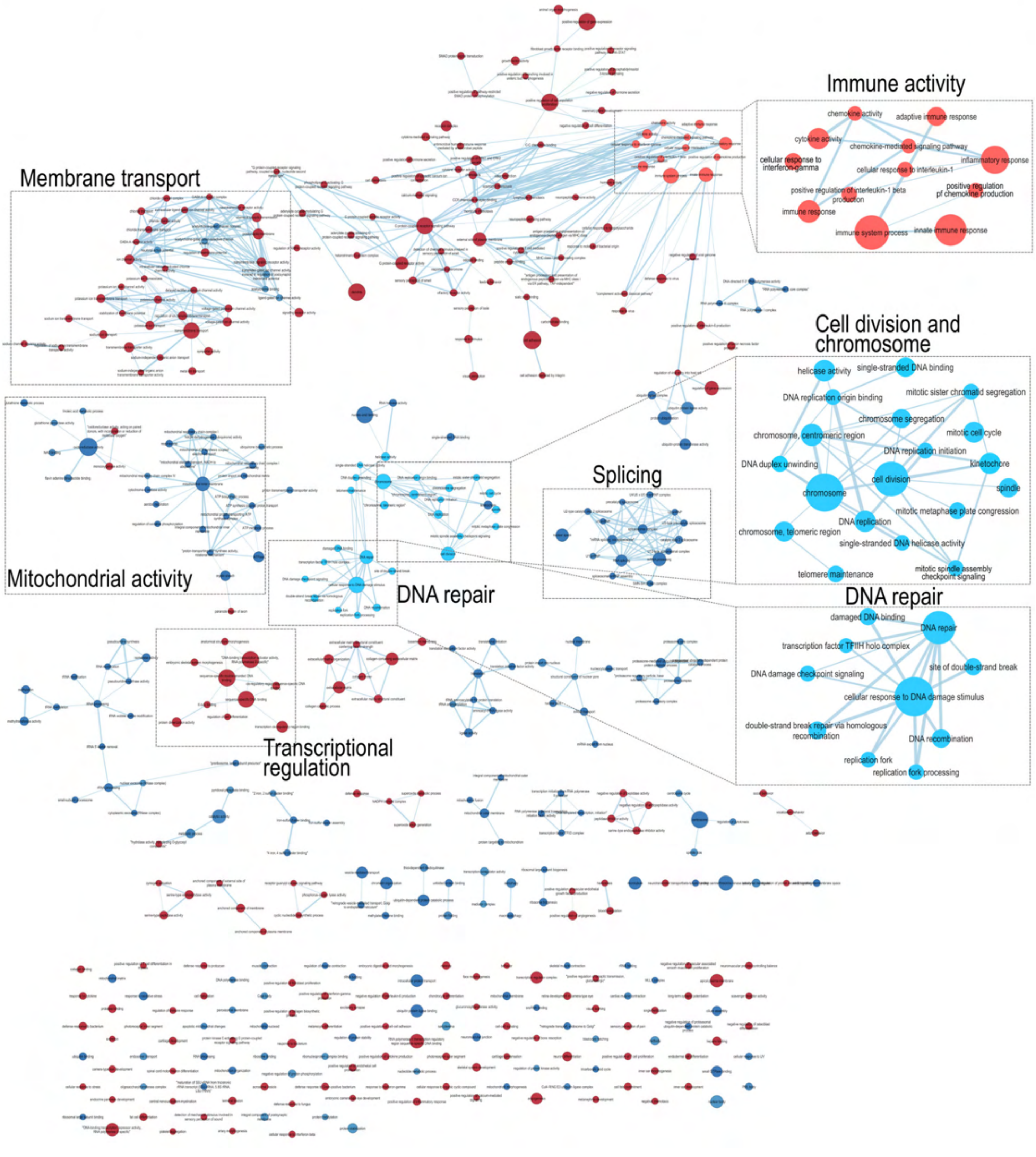

Figure S6

Complete results of the Gene Set Ontology Analysis based on the RNA-seq from H3.3-G34R versus H3.3-Wt mouse pHGG cells. The relevant families of gene ontologies are highlighted. The diameter of the node indicates the number of genes of the Gene ontology. Ontologies in blue are downregulated in H3.3-G34R, and ontologies in red are upregulated in H3.3-G34R pHGG mouse cells.

Supplementary Figure 7

Expression levels of DNA repair genes: mouse pHGG

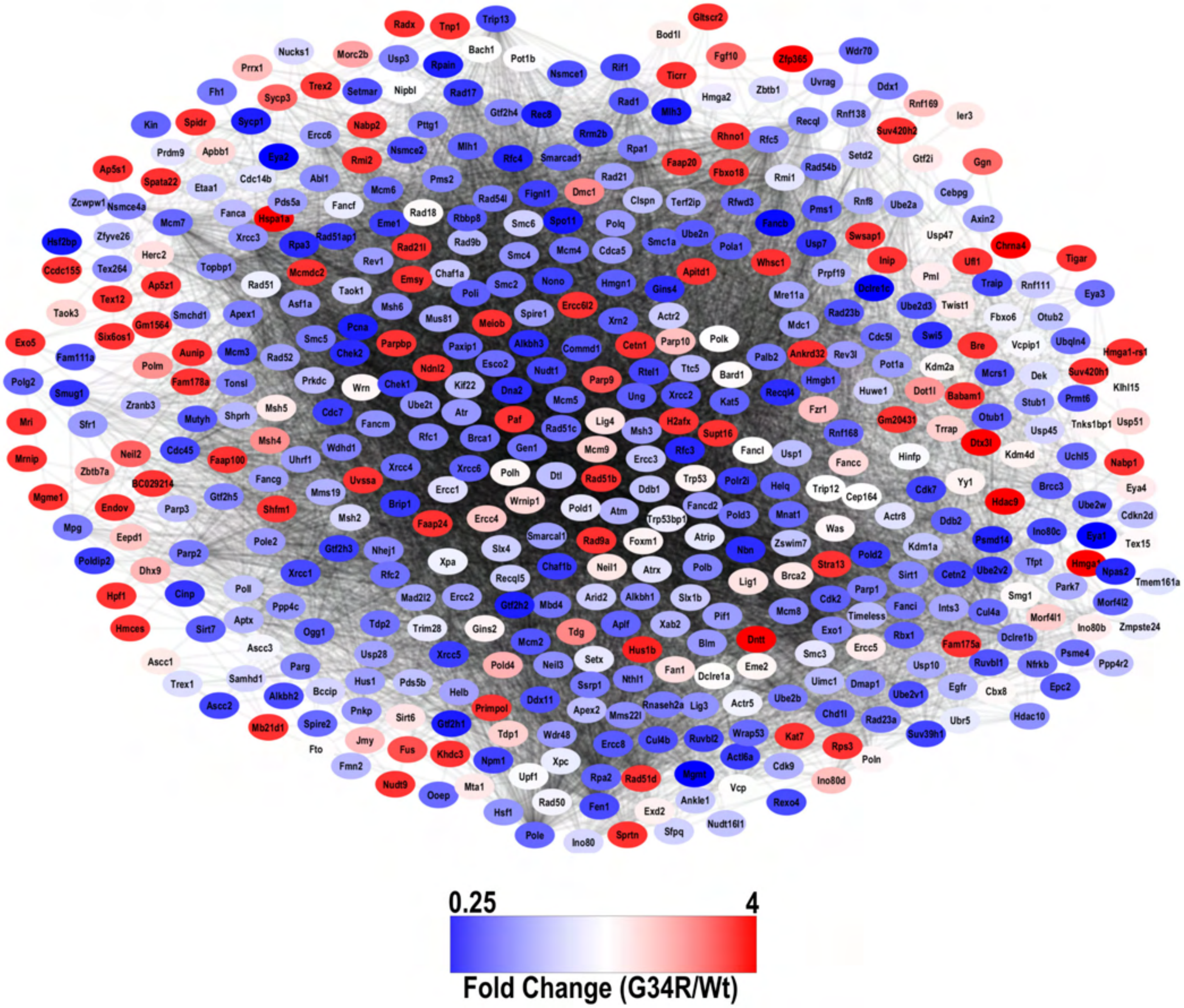

Figure S7

Full network of genes belonging to the Gene Ontology “DNA repair”, and their relative expression (G34R/Wt) expressed with the color code (Blue: downregulated in G34R, Red: upregulated in G34R)

Supplementary Figure 8

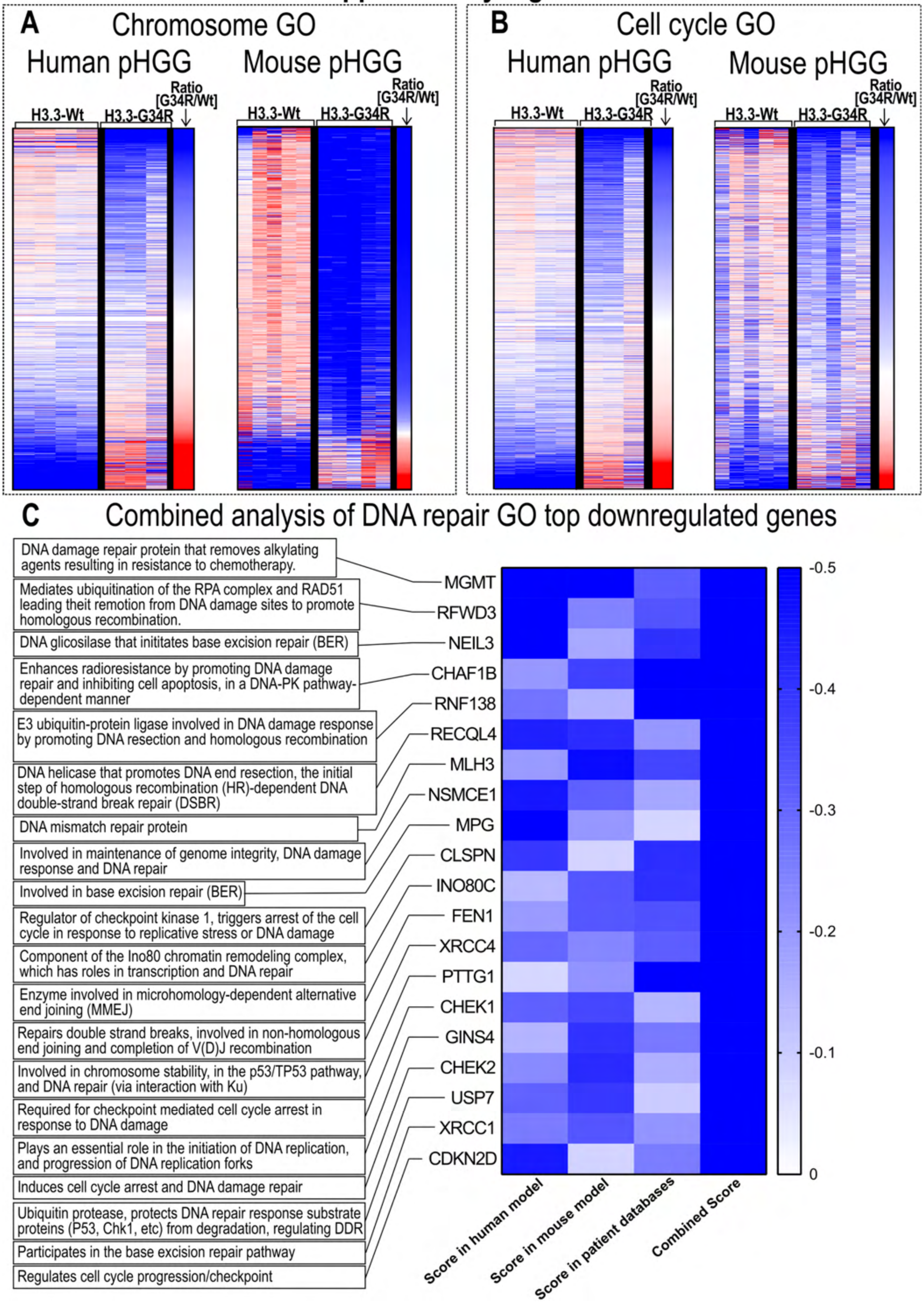

Figure S8

A) Heatmap of genes belonging to the Gene Ontology “Chromosome” in H3.3-G34R versus H3.3-Wt human and mouse pHGG models. **B)** Heatmap of genes belonging to the Gene Ontology “Cell Cycle” in H3.3-G34R versus H3.3-Wt human and mouse pHGG models. **C)** Heatmap depicting a combined analysis highlighting the DNA repair GO genes that are most downregulated in G34R pHGG in the mouse model, the human model and the pHGG patients.

Supplementary Figure 9

Human pHGG: Full network of Differentially expressed Gene Ontologies (from RNA-seq)

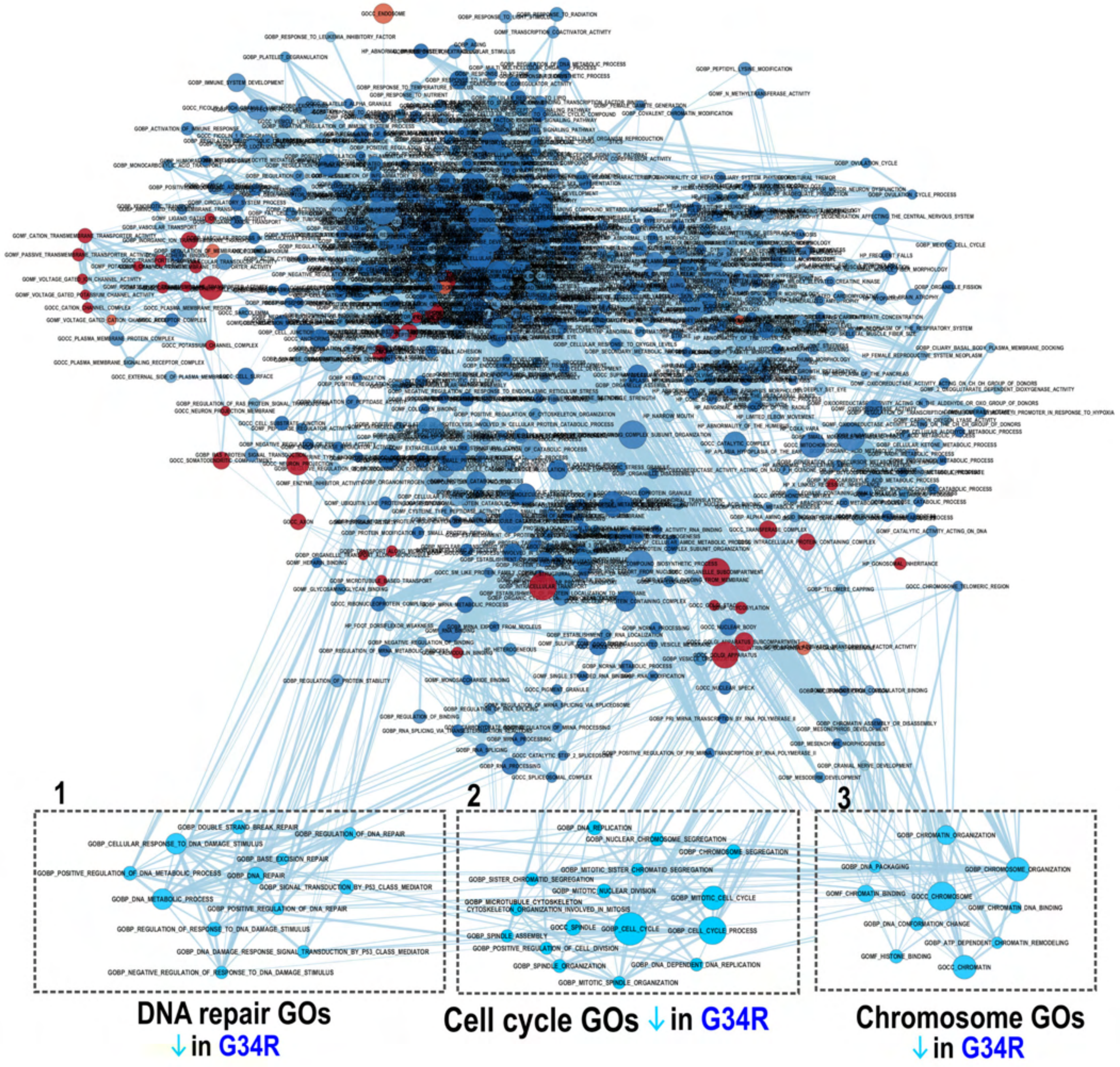

Figure S9

Full Gene Set Ontology Analysis (GSEA) based on RNA-seq of human H3.3-G34R vs. H3.3-Wt pHGG, depicting differentially expressed Gene Ontologies (GOs). The diameter of the node indicates the number of genes of the Gene ontology. Downregulated GOs in H3.3-G34R vs H3.3-Wt are depicted in blue, and upregulated GOs in H3.3-G34R vs H3.3-Wt are depicted in red. The most prominent GO groups are indicated (1= DNA repair-related GOs; 2= Cell cycle-related GOs; 3= Chromosome-related GOs).

Supplementary Figure 10

DNA repair Gene ontology analysis in human pHGG: gene expression network

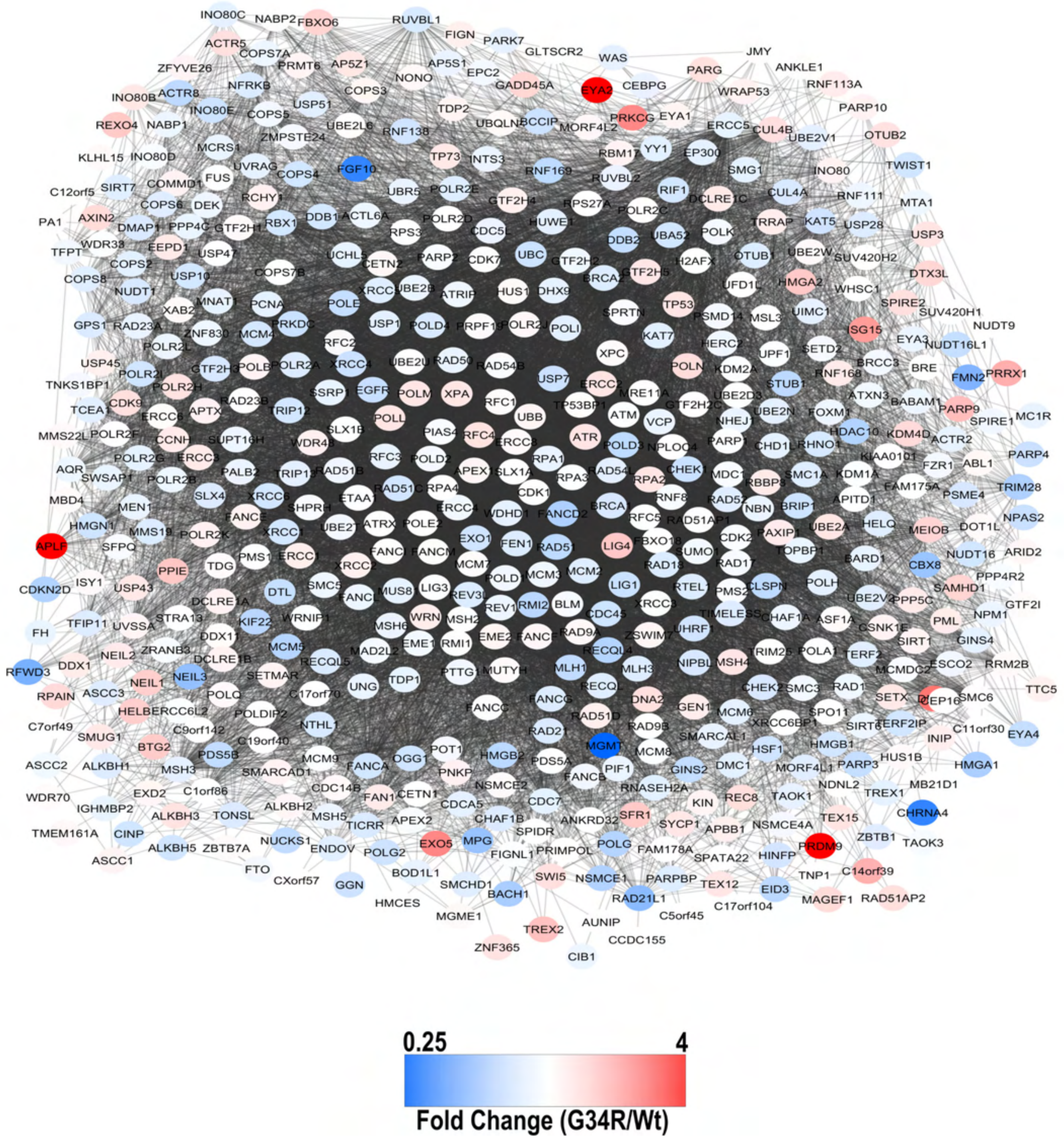

Figure S10

Full network of genes belonging to the Gene Ontology "DNA repair", and their relative expression in the human pHGG model (G34R/Wt) is expressed with the color code (Blue: genes downregulated in G34R, Red: genes upregulated in G34R)

Supplementary Figure 11

Transcriptional activity of DNA repair genes from patients databases

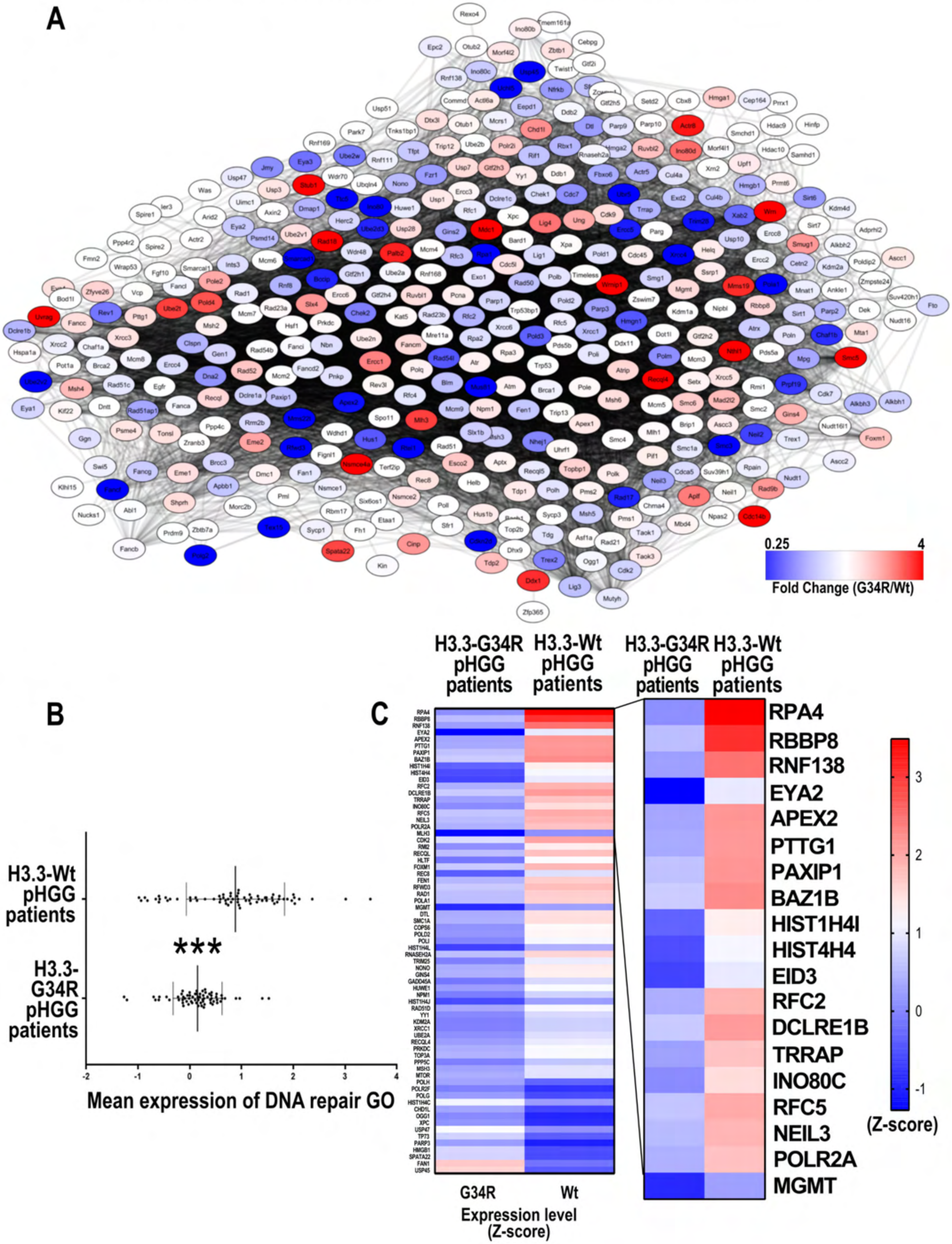

Figure S11

Analysis of the expression of genes belonging to the Gene Ontology "DNA repair" from patient databases. H3.3-G34R pHGG were compared with H3.3-Wt ATRX-mutant and P53-mutant patients. **A)** Full network of genes belonging to the Gene Ontology "DNA repair", where the relative expression in H3.3-G34R versus H3.3-Wt patients (G34R/Wt) is expressed with the color code (Blue: genes downregulated in G34R patients, Red: genes upregulated in G34R patients) **B)** Mean expression of genes belonging to the Gene Ontology "DNA repair" in H3.3-G34R and H3.3-Wt patients. **C)** Heatmap of genes belonging to the Gene Ontology "DNA repair" in H3.3-G34R versus H3.3-Wt patients (G34R/Wt) the most differential genes in G34R vs Wt patients are highlighted. (Blue: low relative expression; red: high relative expression) (***$p<0.005$, unpaired t test (B)).

Supplementary Figure 12

Gating strategy for flow cytometry proliferation experiments shown in Figure 3

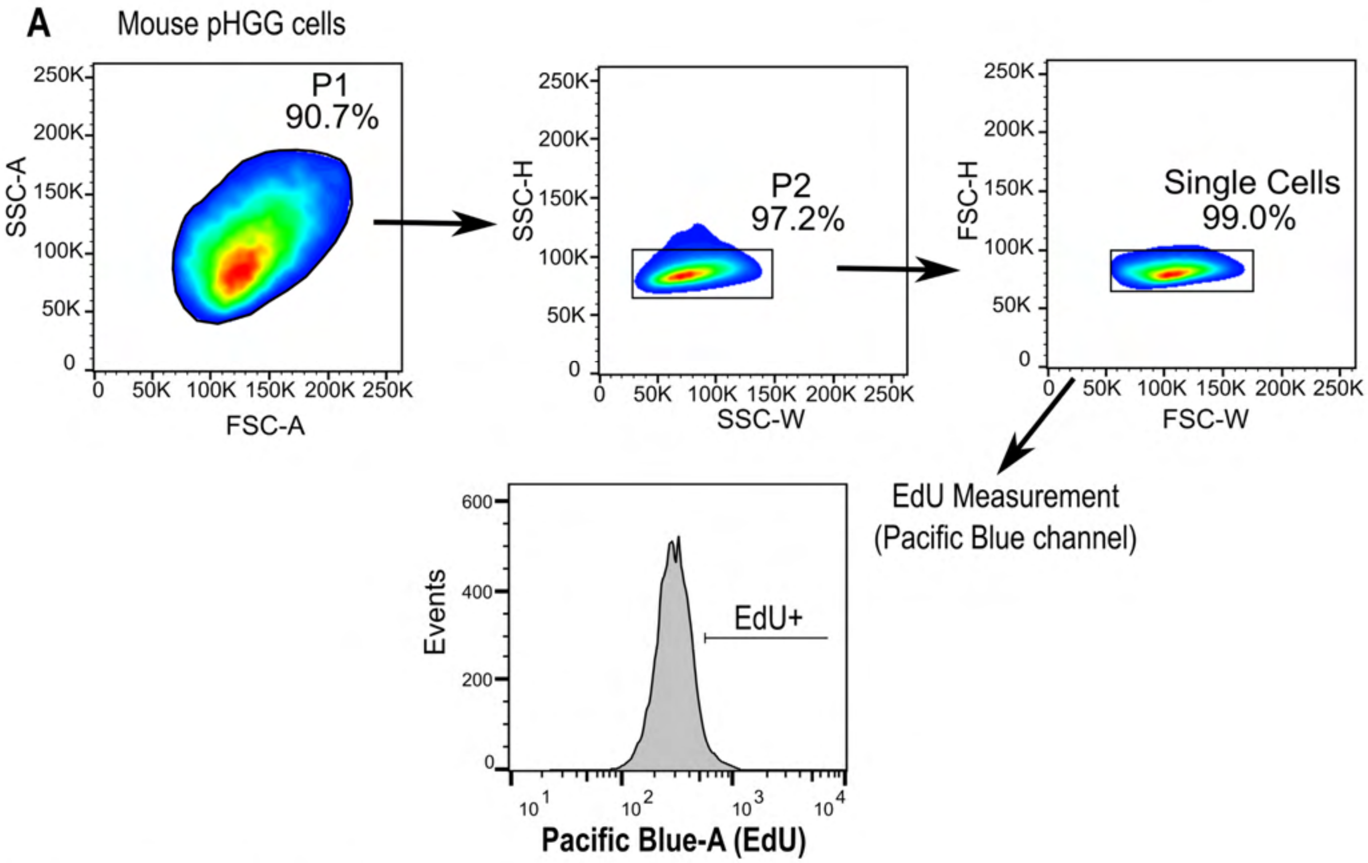

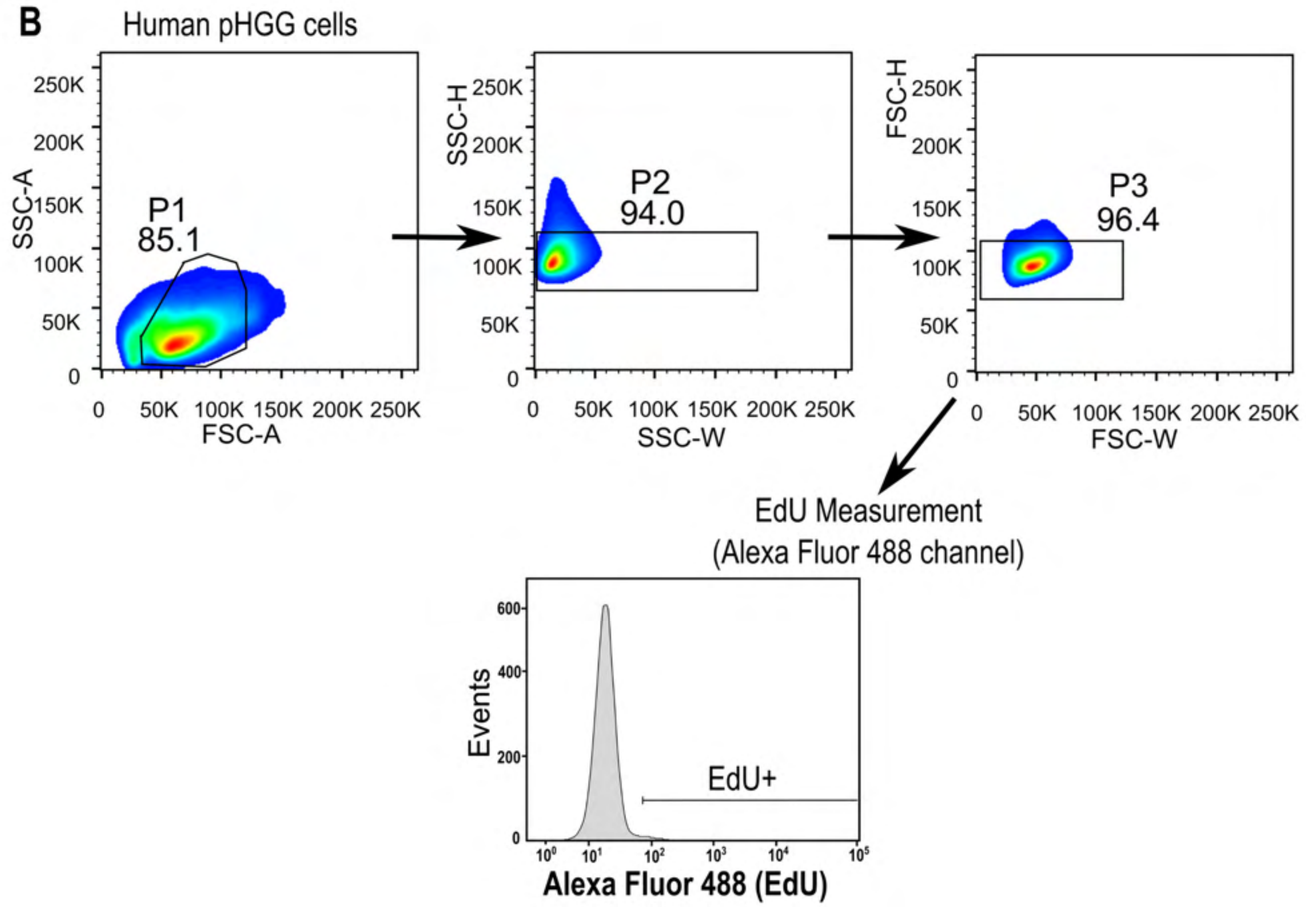

Figure S12

Representative sample depicting the gating strategy utilized for flow cytometry experiments from Figure 3, to assess EdU incorporation levels in mouse **(A)** and human **(B)** cells. EdU positive gating was set to represent <1% of the cells on the isotype negative control samples.

Supplementary Figure 13

Frequency of colony initiating cells *in vitro*, mouse and human pHGG cells

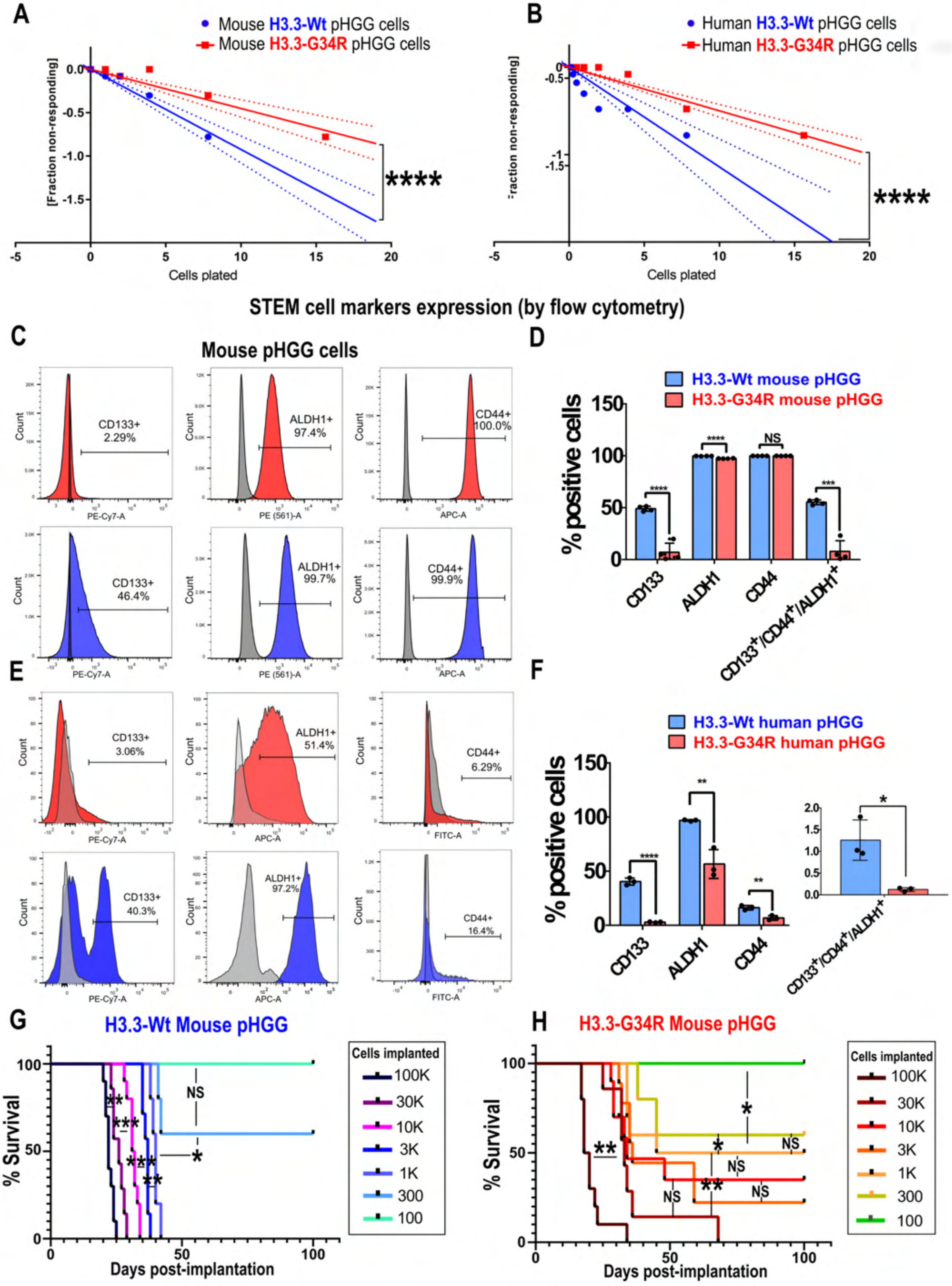

Figure S13: ***Analysis of stem cell properties of H3.3-G34R cells in vitro and in vivo.*** **A)** Frequency of colony initiating cells in mouse H3.3-G34R and H3.3-Wt pHGG cells. (Non-responding = Fraction of wells with no cells). **B)** Frequency of colony initiating cells in human H3.3-G34R and H3.3-Wt pHGG cells. (Non-responding = Fraction of wells with no cells). **(C-D)** Representative flow cytometry plots and quantification of the percentage of the CD133+, ALDH1+, CD44+, and triple positive cells in H3.3-wt (blue) and H3.3G34R (red) in mouse pHGG cells. **(E-F)** Representative flow cytometry plots and quantification of the percentage of the CD133+, ALDH1+, CD44+, and triple positive cells in H3.3-wt (blue) and H3.3G34R (red) in human pHGG cells. **G)** Tumor initiating capacity *in vivo* of H3.3-Wt mouse cells. (DPI = Days post-implantation) **H)** Tumor initiating capacity *in vivo* of H3.3-G34R mouse cells. (DPI = Days post-implantation) *(*$p<0.05$, **$p<0.01$, ***$p<0.005$, ****$p<0.001$; analysis of slope difference from linear regression model (A, B); unpaired t test (D, F); analysis of median survival from Kaplan–Meier model (G-H). Data represent mean ± SD of three experimental replicates (A, B, D, F)).*

Supplementary Figure 14

Determination of triple positive (CD133/ALDH1/CD44) cells by flow cytometry

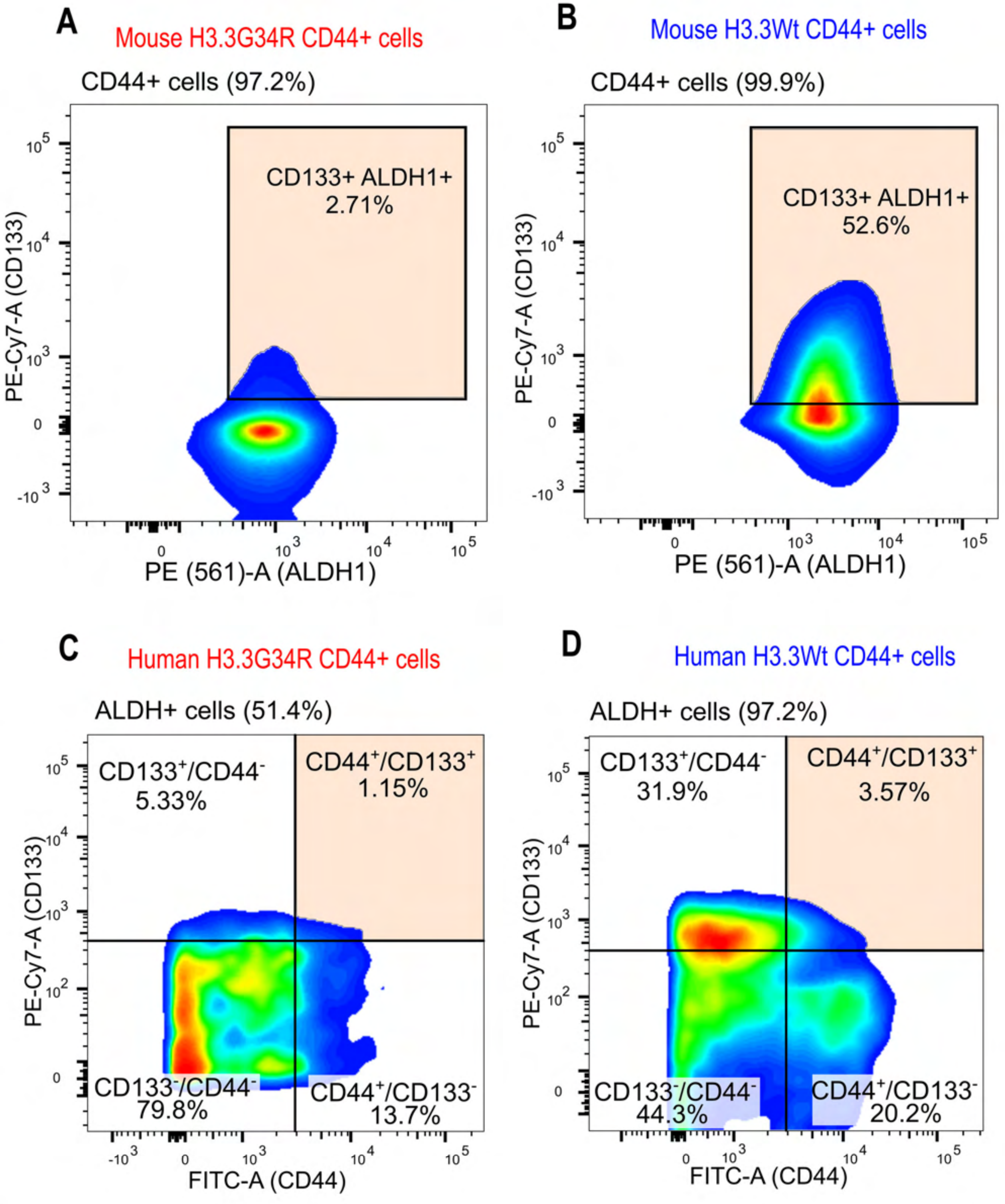

Figure S14

A-B) Representative sample depicting the percentages of triple positive cells (CD44+, CD133+, ALDH+) within the CD44+ cells in mouse H3.3-G34R and H3.3-Wt pHGG cells (from experiment in Fig 4). **C-D)** Representative sample depicting the percentages of triple positive cells (CD44+, CD133+, ALDH+) within the ALDH1+ cells in human H3.3-G34R and H3.3-Wt pHGG cells (from experiment in Figure s13).

Supplementary Figure 15

A Gating strategy for experiment shown in Supplementary Figure 13.C-D (mouse pHGG cells)

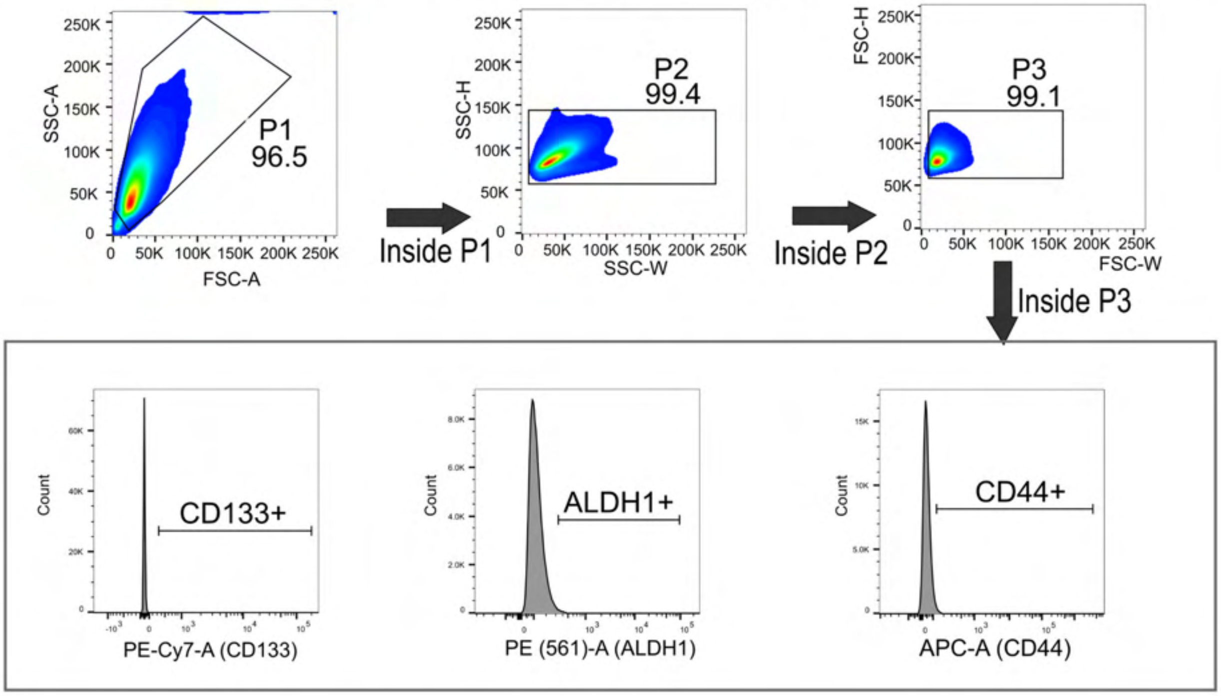

B Gating strategy for experiment shown in Supplementary Figure 13.E-F (human pHGG cells)

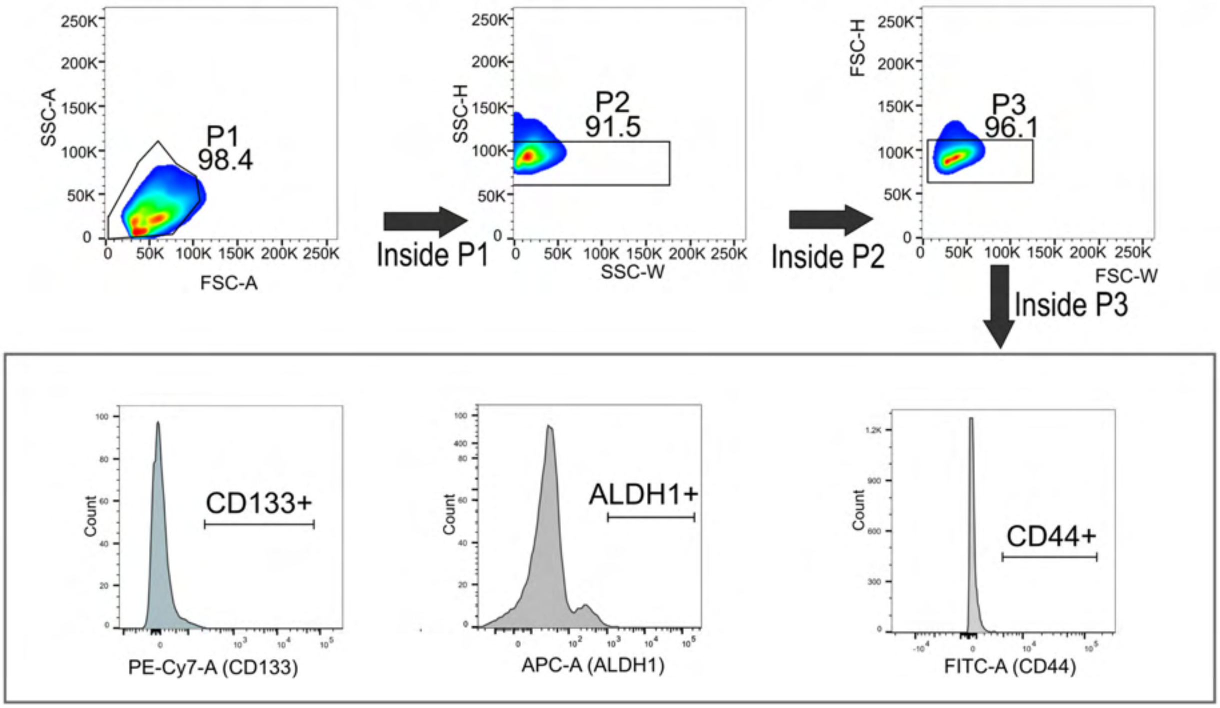

Figure S15

A) Representative sample depicting the gating strategy and fluorescence channels utilized for cytometry experiments in figure 4(C-D) **B**) Representative sample depicting the gating strategy and fluorescence channels utilized for cytometry experiments in figure 4(E-F). CD133, ALDH1 and CD44 positive gratings were set to represent <1% of the cells on the respective isotype negative control samples.

Supplementary Figure 16

DNA repair activity reporter assay: fluorescence microscopy

A

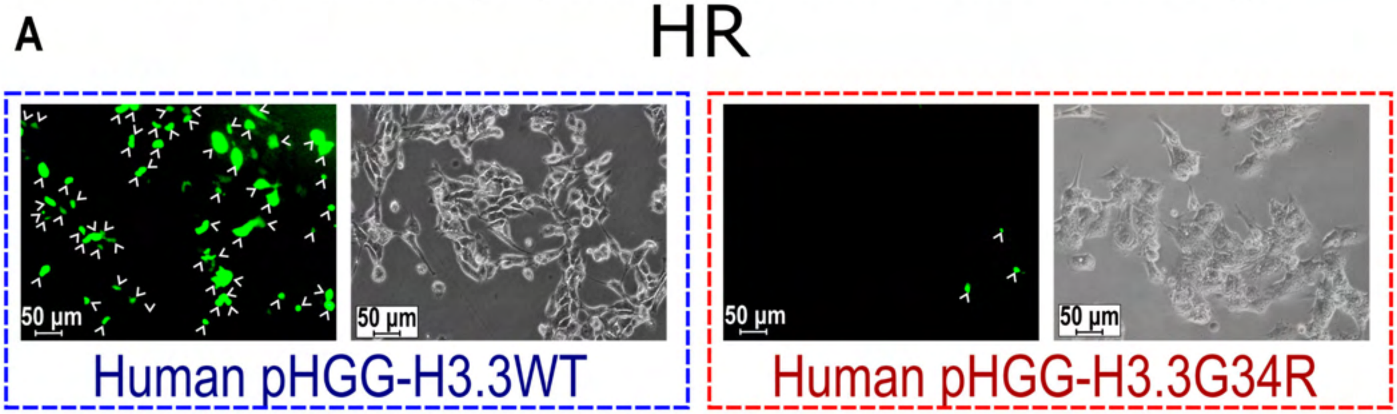

B

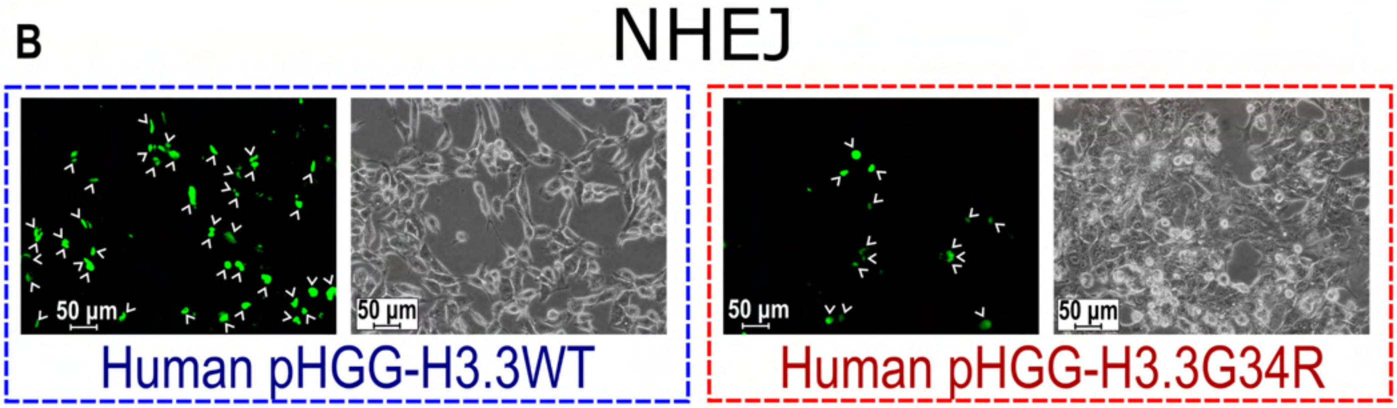

Figure S16

Fluorescence microscopy images of human pHGG cells transfected with HR (A) and NHEJ (B) reporter plasmids illustrating the DNA repair activities via HR and NHEJ in H3.3-G34R and H3.3-Wt cells. Arrowheads indicate GFP positive cells.

Supplementary Figure 17

Chromatin accessibility and DNA repair activity in human endogenous H3.3-G34R/H3.3-Wt pHGG cells

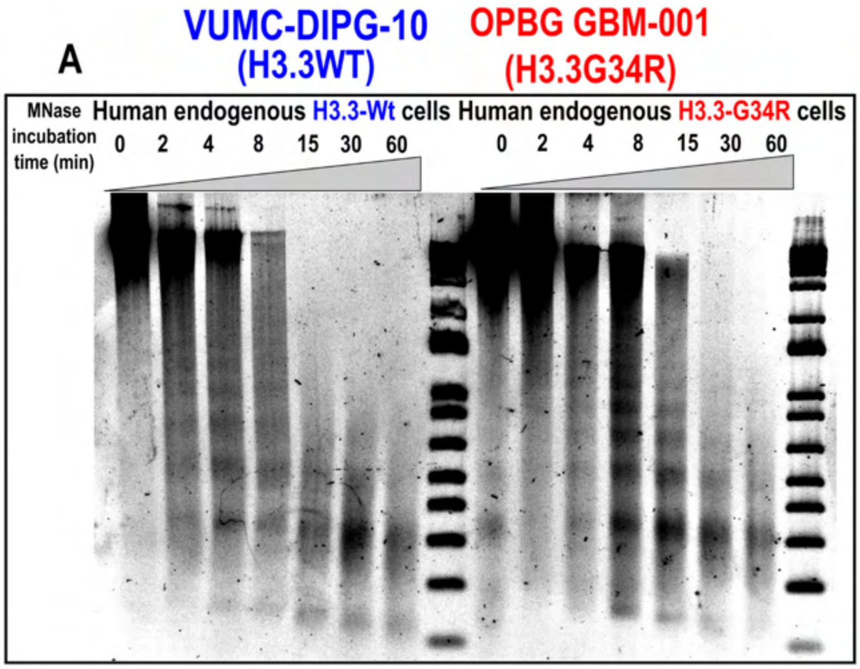

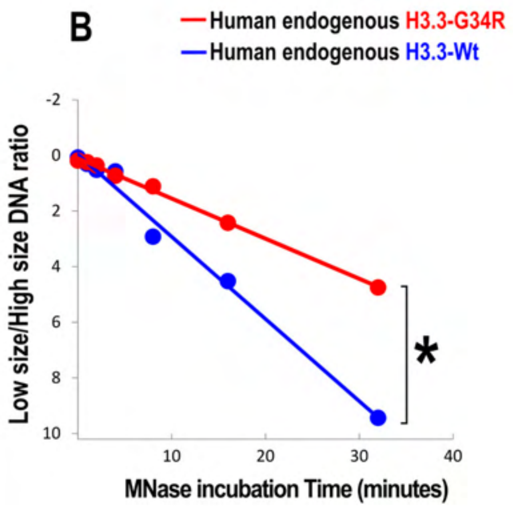

DNA repair activity: Human endogenous H3.3-G34R/H3.3-Wt pHGG

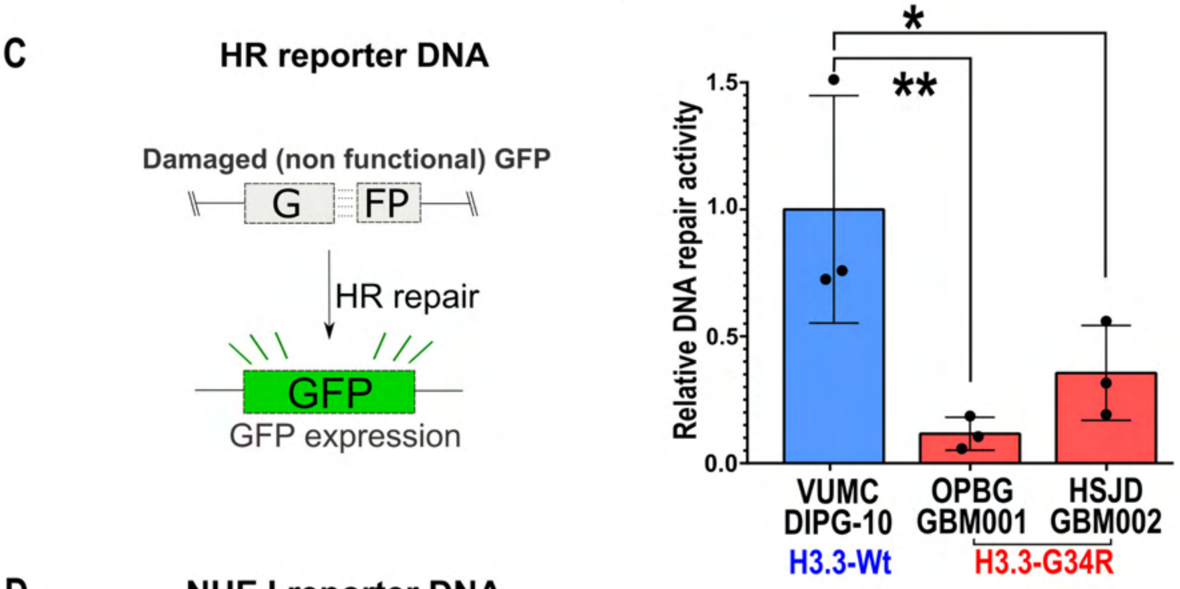

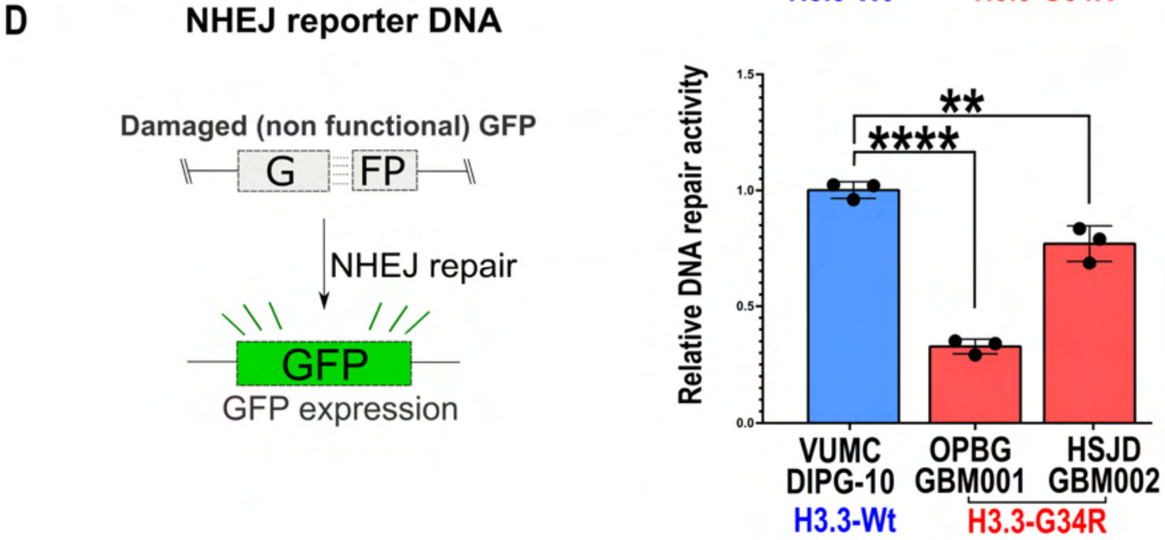

Figure S17

A) DNA electrophoresis gel depicting the results of a Micrococcal nuclease (MNase) chromatin accessibility assay performed in endogenous histone mutant (H3.3-G34R) and histone wild type (H3.3-Wt) patient derived cells, and **B)** statistical analysis of this experiment. **C-D)** Results of a DNA repair activity assay performed in endogenous histone mutant (H3.3-G34R) and histone wild type (H3.3-Wt) patient derived cells, showing the homologous recombination (HR) and non-homologous end joining NHEJ basal DNA repair activities of each cell. *(*p<0.05, **p<0.01, ***p<0.005, ****p<0.001; analysis of slope difference in nonlinear regression model (B); unpaired t test (C-D). Data represent mean ± SD of three experimental replicates (C-D)).*

Supplementary Figure 18

Analysis of double strand breaks by neutral comet assay

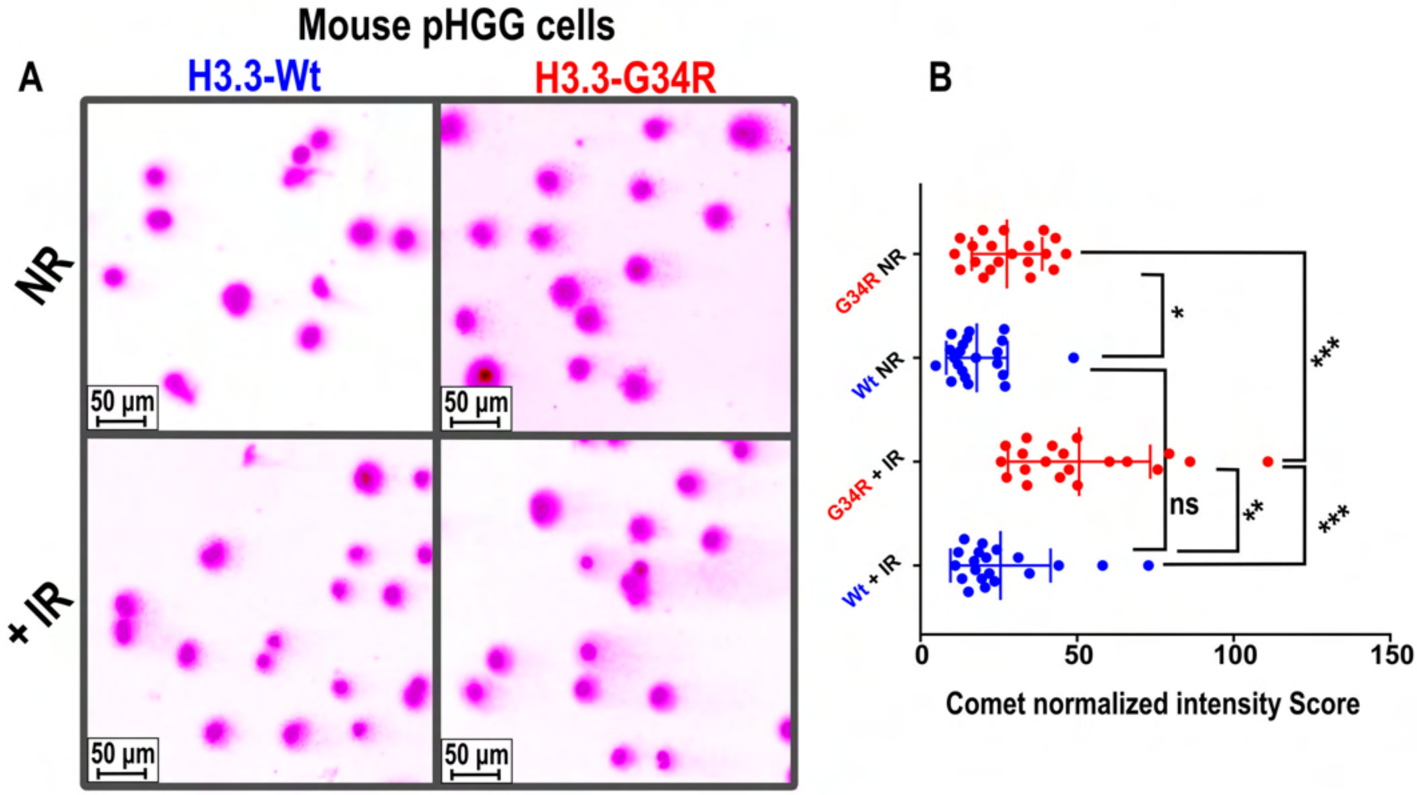

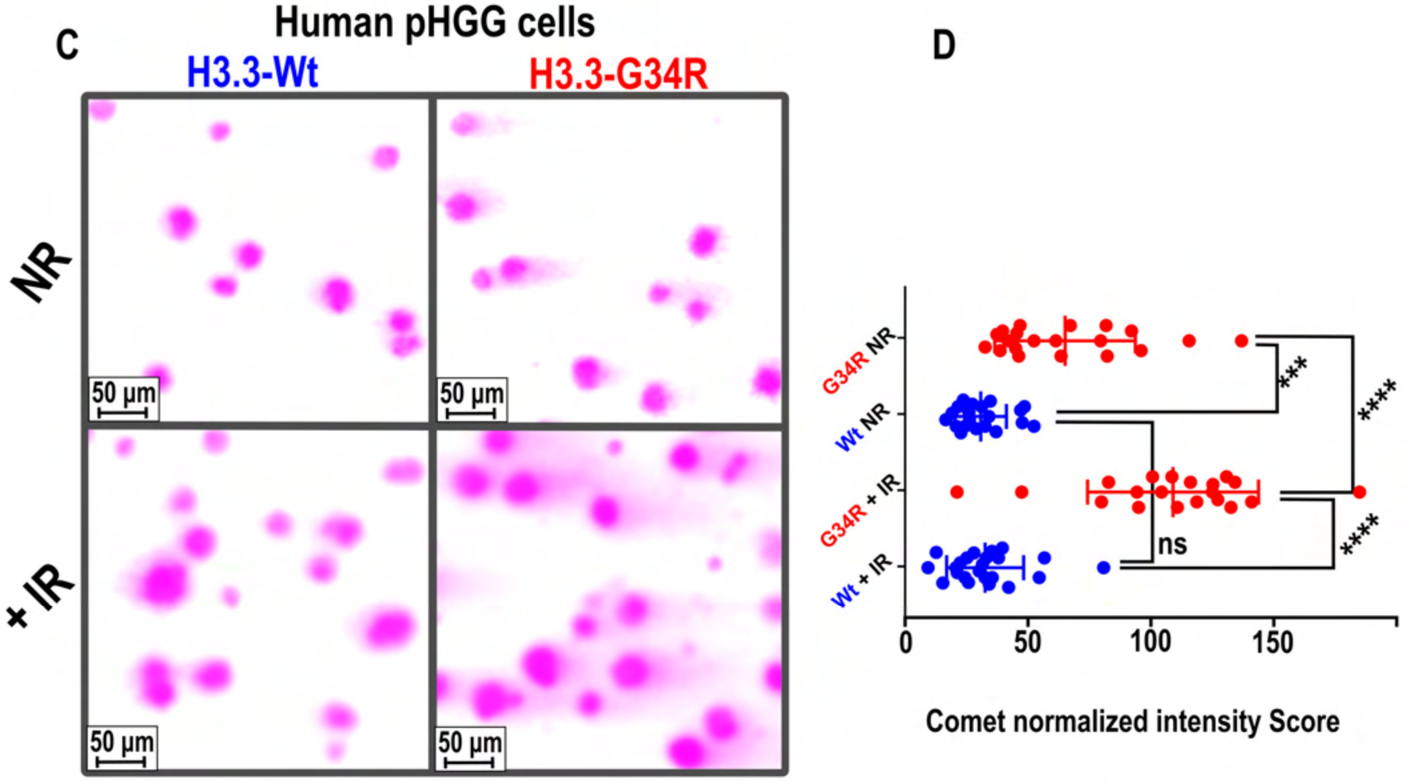

Figure S18

Fluorescence microscopy images of neutral comet assays in **A)** mouse and **C)** human cells in basal conditions and after ionizing radiation (4 hours post IR, 3 Gy), and quantification of the results **(B, D)**. (NR=non-irradiated). *(***p<0.005, ****p<0.001; One-way Anova test (B, D). Data represent the mean of the comet intensity of individual cells from four different fields of view).*

Supplementary Figure 19-A

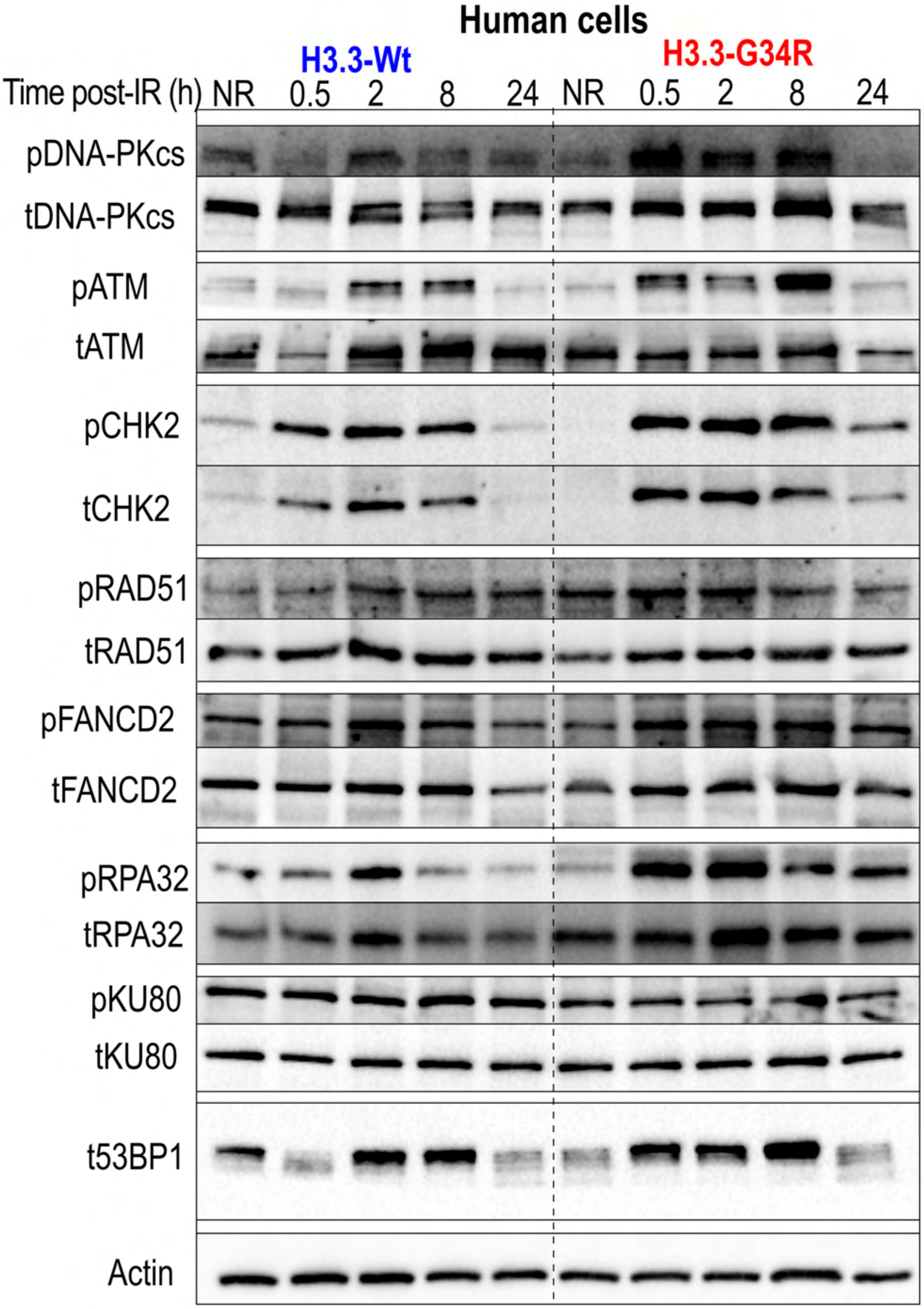

Figure S19-A: ***Expression of DNA repair proteins and posttranslational modifications (PTMs) levels in H3.3-G34R pHGG*****. A)** Western blotting of selected DNA repair proteins and posttranslational modifications (PTMs) assessing the activation of these proteins in human pHGG cells.

Supplementary Figure 19-B

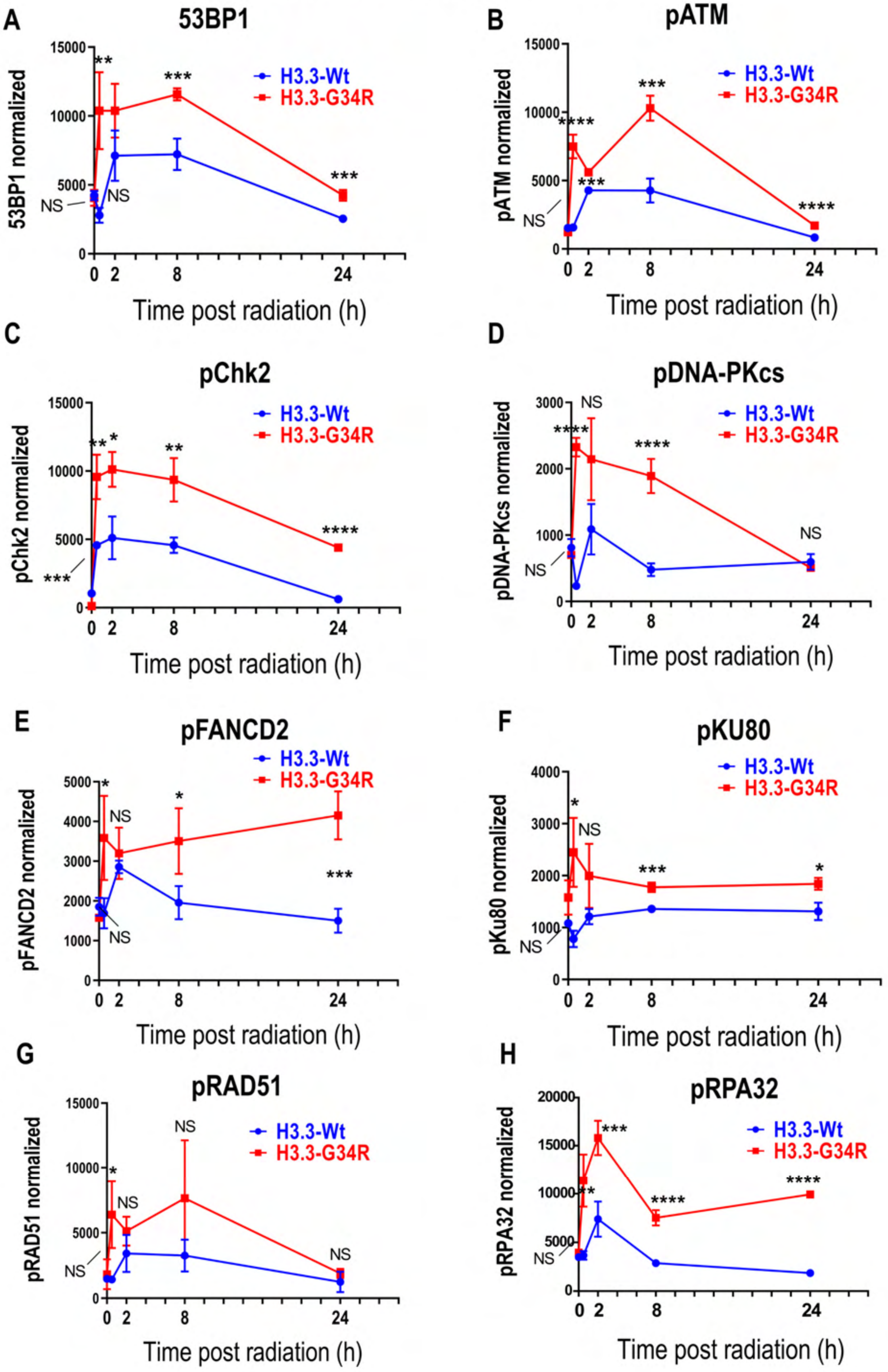

Figure S19-B: ***Expression of DNA repair proteins and posttranslational modifications (PTMs) levels in H3.3-G34R pHGG***. Quantification of Western blot results showing normalized levels of ATM-pSer1981 **(C)**, CHK2-pThr68 **(B)** (DNA damage sensing/Checkpoint activation), DNA-PKs-pSer2056 **(D)**, Ku80-p, **(F)** total 53BP1 **(A)** (NHEJ); and RAD51-pThr309 **(G)** RPA32-pSer33 **(H)** and FANCD2-pThr649 (HR). (*p<0.05, **p<0.01, ***p<0.005, ****p<0.001; unpaired t test (C-E)). Data represent mean ± SD. *(*p<0.05, **p<0.01, ***p<0.005, ****p<0.001; unpaired t test (C-E)). The actin control is a representative loading of all the protein samples. Data represent mean ± SD. N=3 technical replicates)*

Supplementary Figure 20

Cell cycle Chk2 activation in human pHGG upon ionizing radiation

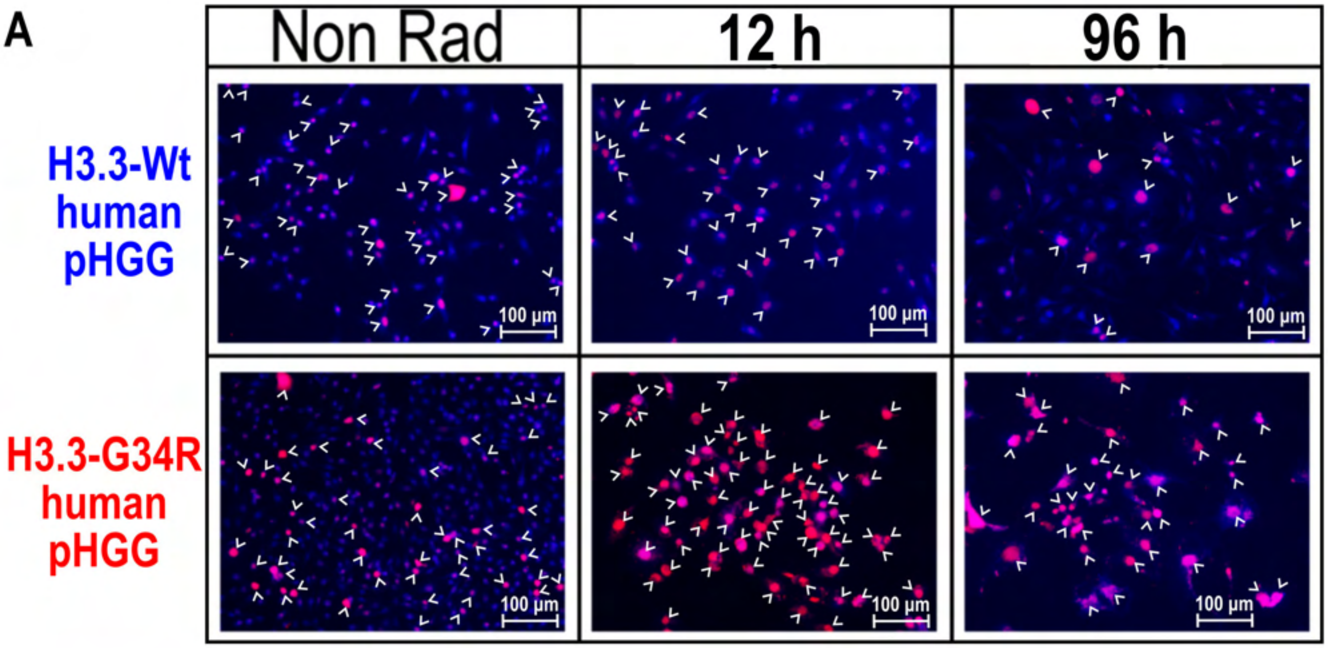

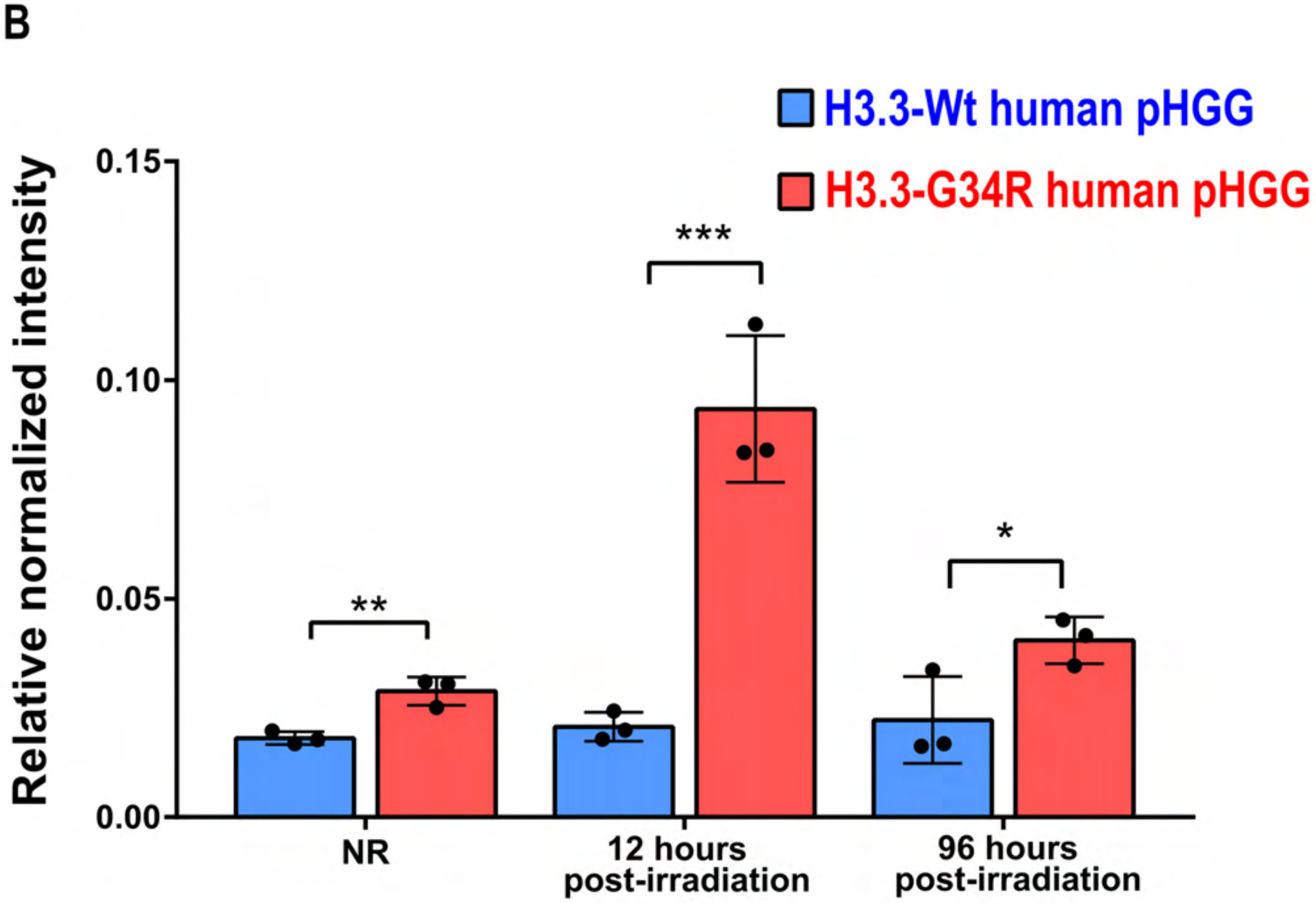

Figure S20

A) Immunofluorescence for Chk2-phosphoThr68 (in red and nuclei (in blue), assessing cell cycle checkpoint 2 activation in response to ionizing radiation (IR) in human H3.3-Wt and H3.3-G34R pHGG cells. Arrowheads indicate Chk2-pThr68 positive cells. **B)** Quantification and statistical analysis of the results in A). *(*p<0.05, **p<0.01, ***p<0.005, ****p<0.001; unpaired t test. Data represent mean ± SD of five different field of views).*

Supplementary Figure 21

A DNA repair pathway and protein/PTM levels [G34R/Wt], mouse model

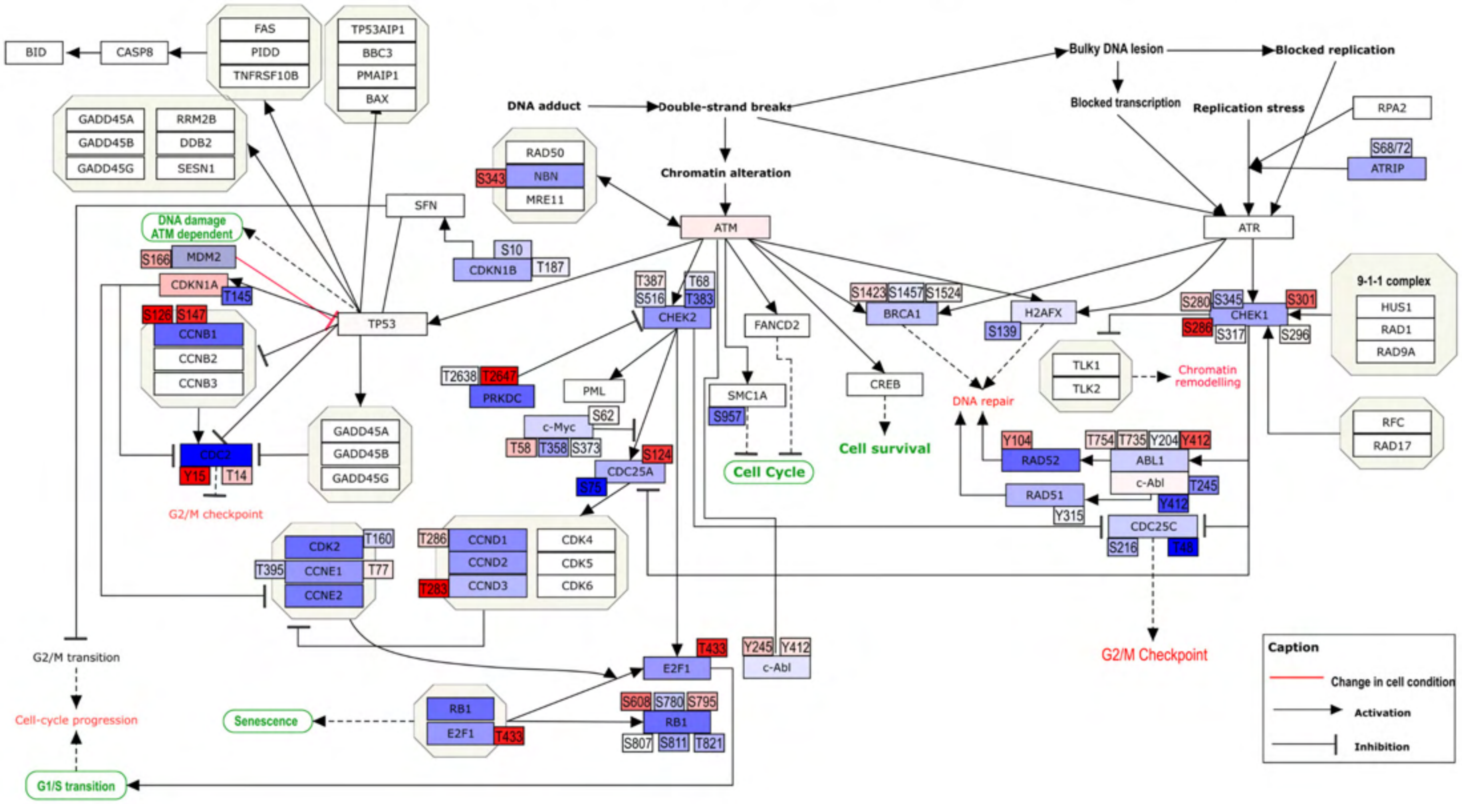

B DNA repair pathway and protein/PTM levels [G34R/Wt], human model

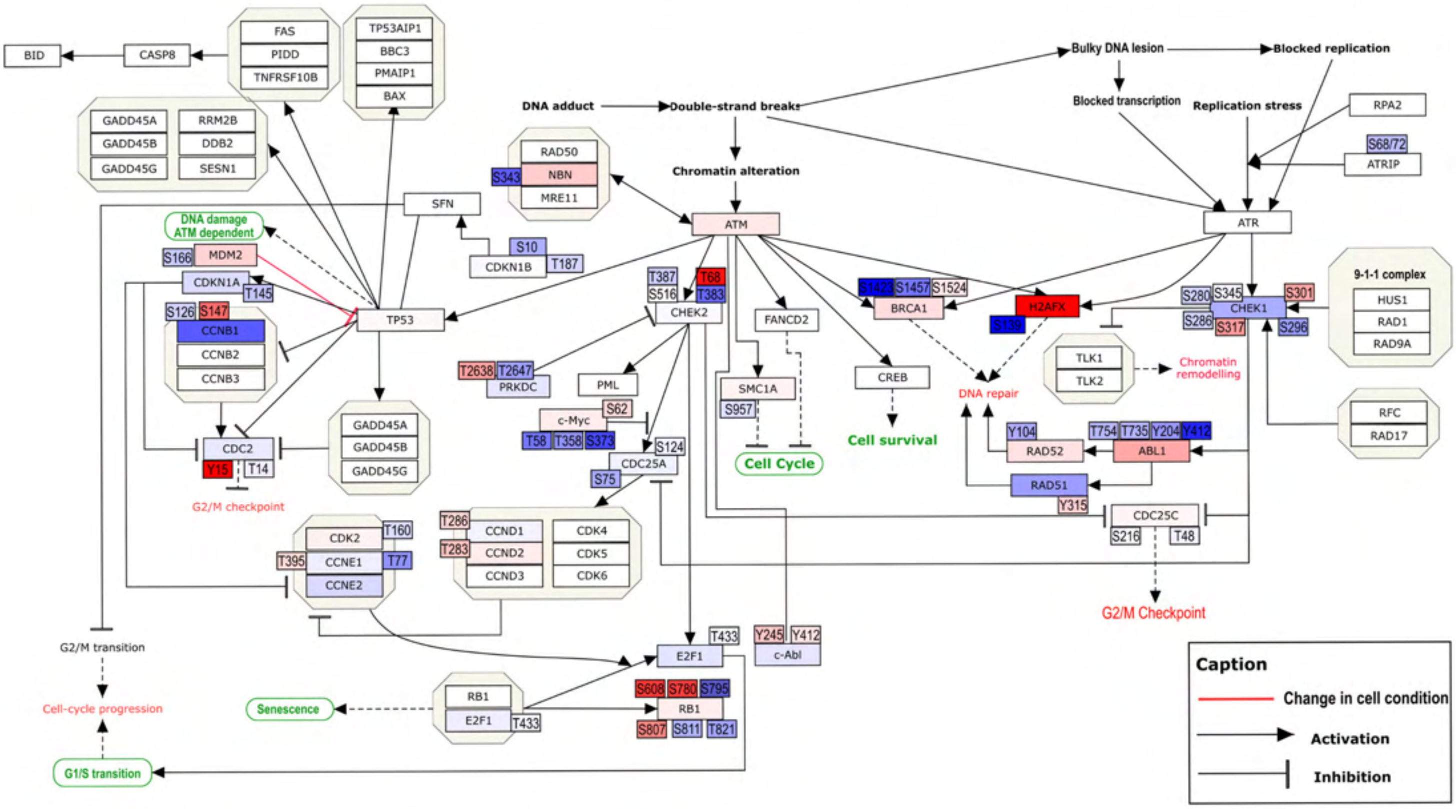

Figure S21

Analysis of DNA repair pathway activity by protein and PTMs levels in mouse and human pHGG G34R cells. **A-B)** Scheme depicting the DNA repair pathway, and the results of the array in each protein and PTM in a color code defined by relative expression (Fold change) between G34R versus Wt pHGG cells (Blue: downregulated, red: upregulated), in the mouse **(A)** and human **(B)** models.

Supplementary Figure 22

Response to Temozolomide

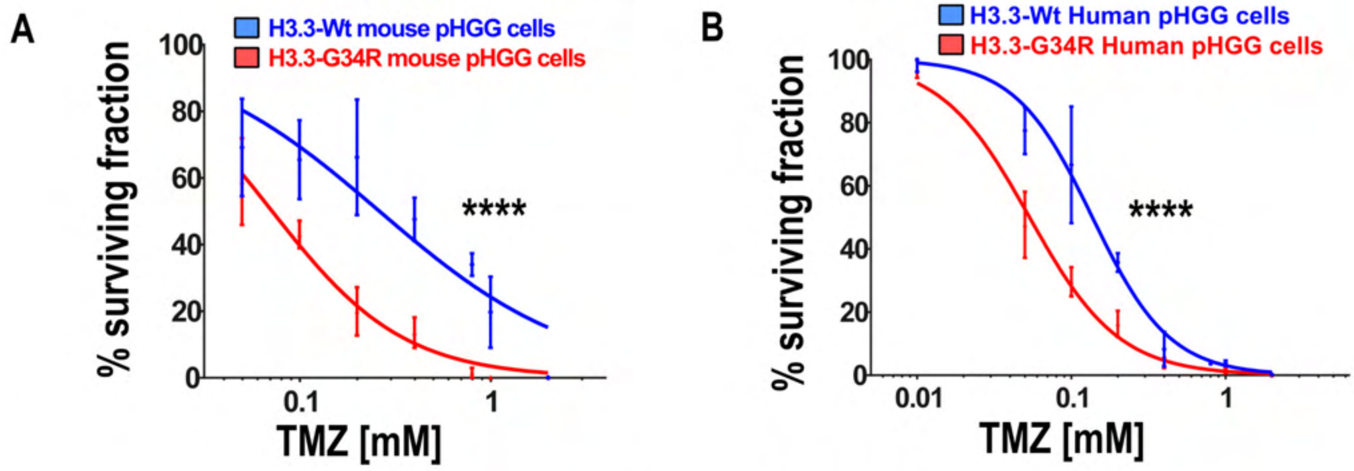

Dose response to IR and Pamiparib, CDKN2A-/- pHGG GEMM

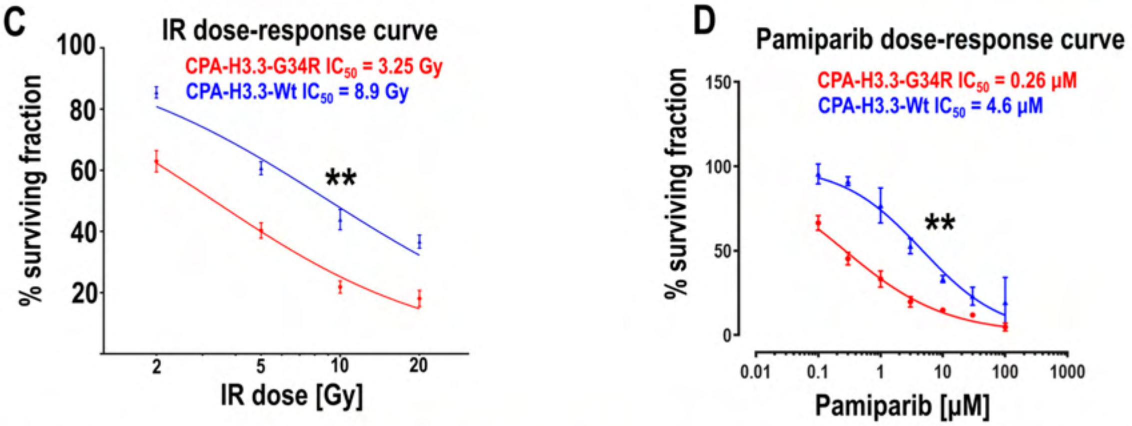

Dose response to CHK1/2i AZD7762 and PARPi Pamiparib in endogenous H3.3-G34R patient derived cells

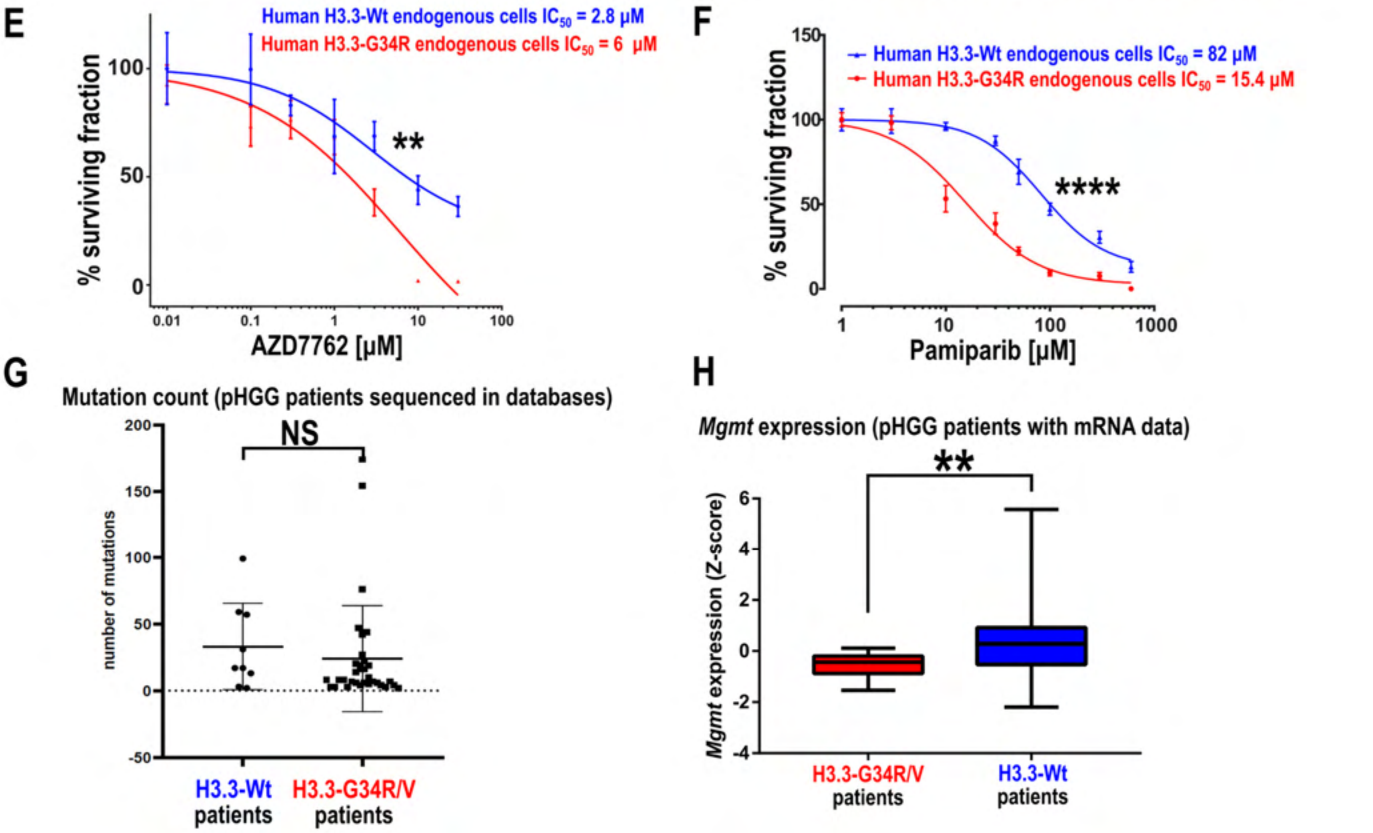

Figure S22

A-B) Analysis of the response to Temozolomide of H3.3-G34R and H3.3-Wt pHGG mouse **(A)** and human **(B)** cells. **C-D)** Analysis of the response to ionizing radiation (IR) **(C)** and PARPi pamiparib **(D)** of H3.3-G34R and H3.3-Wt pHGG cells developed using an alternative sleeping beauty-based Genetically Engineered Mouse Model (GEMM) of pHGG where tumors are induced with CDKN2A-/-, and expression of a constitutively activated PDGFRα (D842V mutation), and independent of receptor tyrosine kinase (RTK)/RAS/PI3K activation. **E-F)** Analysis of the response to cell cycle checkpoint inhibitor AZD7762 **(E)** and PARPi pamiparib **(F)** of endogenous H3.3-G34R-mutant and endogenous H3.3-Wt pHGG cells. **G)** Comparison of the number of point mutations in histone mutant and histone wild type pHGG, from patient databases. **H)** Analysis of expression of the *Mgmt* gene in human H3.3-G34R/V patients vs H3.3-Wt patients (From PedcBioportal database). *(*$p<0.05$, **$p<0.01$, ***$p<0.005$, ****$p<0.001$; analysis of Hill slope difference in nonlinear sigmoid regression model (A-F); Wilcoxon test (G-H). Data represent mean ± SD of three experimental replicates).*

Supplementary Figure 23

Clonogenic Assay in response to temozolomide (TMZ) combined with ionizing radiation (IR)

A

Mouse pHGG cells

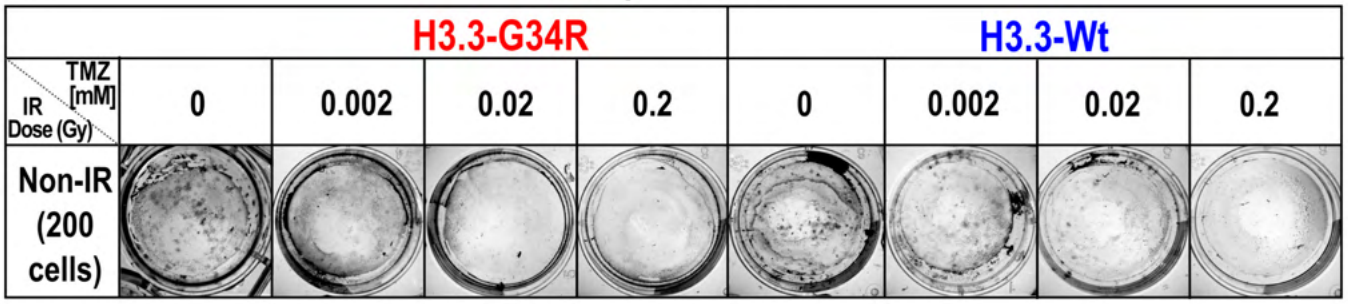

B

Human pHGG cells

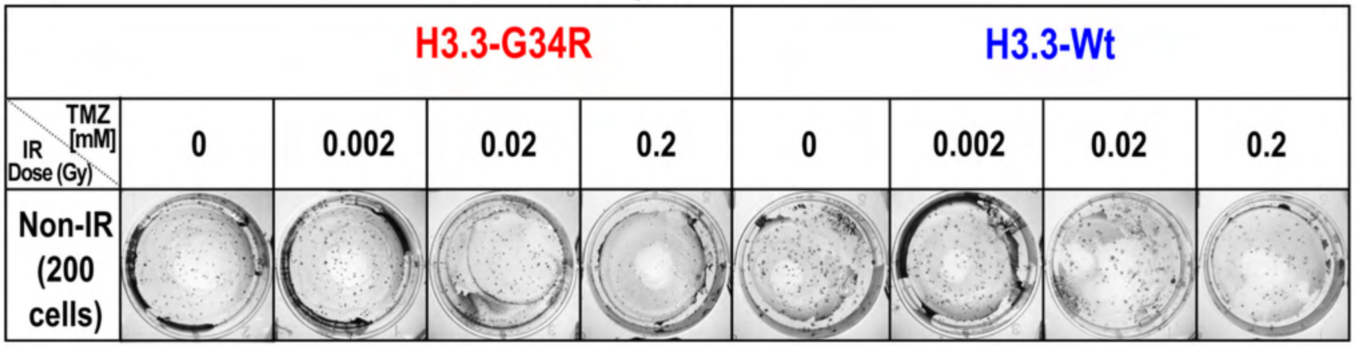

C

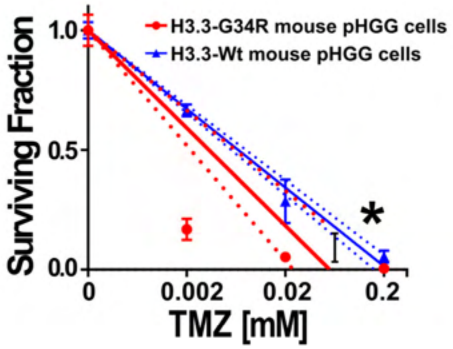

D

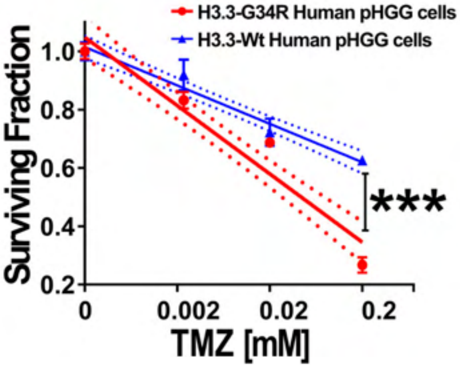

Figure S23

A-B) Clonogenic assay to assess the response of H3.3-G34R and H3.3-Wt pHGG mouse **(A)** and human **(B)** cells to Temozolomide **C-D)** Curves representing the fraction of surviving colonies in each condition. *(*p<0.05, **p<0.01, ***p<0.005, ****p<0.001; slope difference in linear regression model. Data represent mean ± SD of three experimental replicates. The surviving fraction is referred to the non-treated cells (0 mM TMZ).*

Supplementary Figure 24

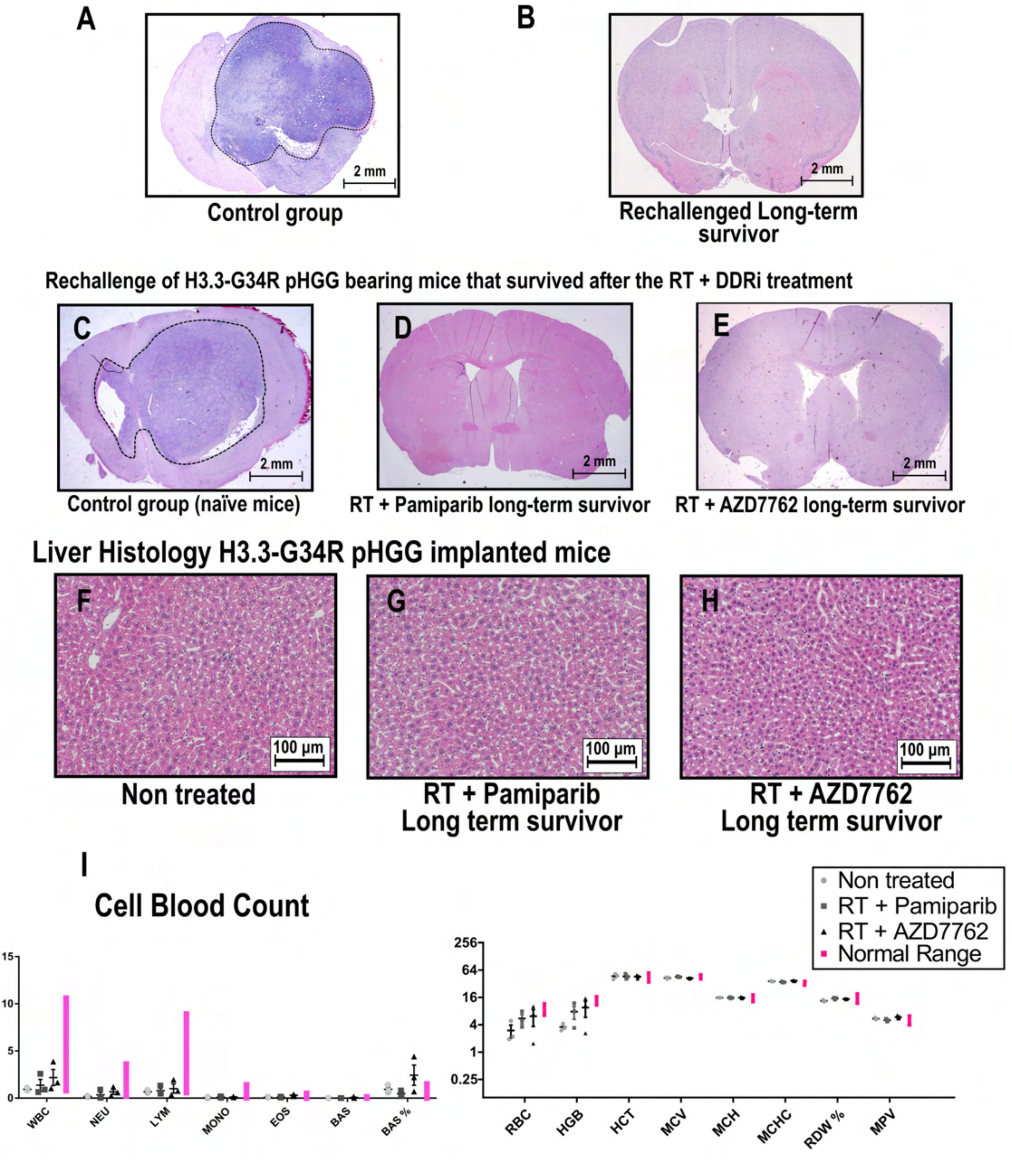

Figure S24

A-B) Representative Hematoxylin and Eosin stainings of fixed brain sections of A) naïve animals (Control group) or B) Long term survivors rechallenged with H3.3-G34R pHGG. **C-E)** Representative Hematoxylin and Eosin stainings of D) fixed brain sections of naïve animals (Control group) or E-F) long term survivors of RT + DDRi treated-mice rechallenged with H3.3-G34R pHGG. **F-H)** Representative Hematoxylin and Eosin stainings of fixed liver sections of animals implanted with H3.3-G34R pHGG, without treatment or subjected to RT + pamiparib or RT + AZD7762 treatments. **I)** Cell blood count of animals implanted with H3.3-G34R pHGG, without treatment or subjected to RT + pamiparib or RT + AZD7762 treatments. (WBC= Whole blood count, NEU= Neutrophiles, LYM= lymphocytes, MONO= Monocytes, EOS= eosinophiles, BAS= Basophiles, RBC = Red blood cells count, HGB= Hemoglobin, HTC= hematocrit, MCV= Mean corpuscular volume, MCH= Mean corpuscular hemoglobin, MCHC= mean corpuscular hemoglobin concentration, RDW%= red cell distribution width, MPV= Mean Platelet Volume.) Normal Ranges for each measure are represented with pink lines.

Supplementary Figure 25

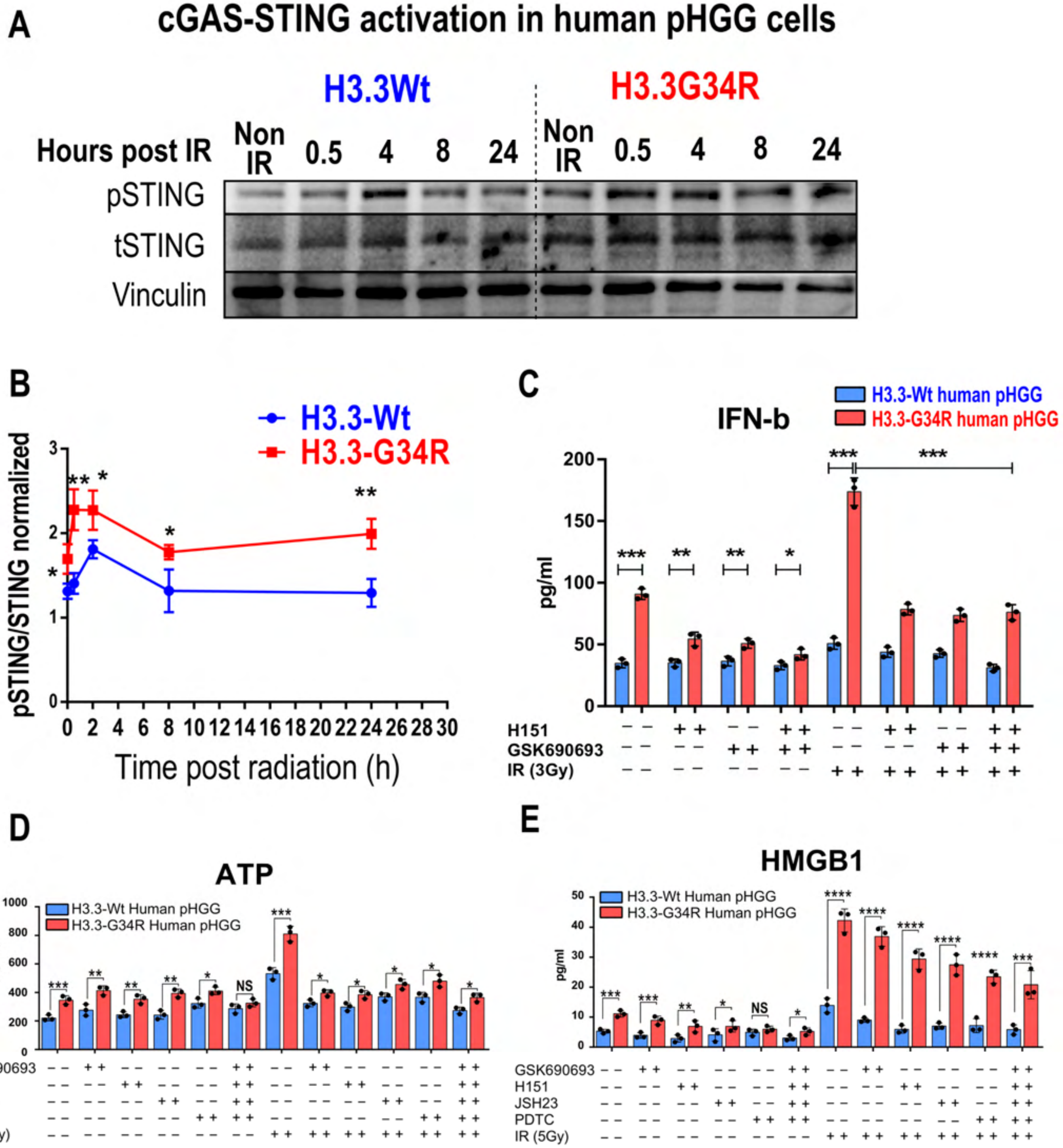

Figure S25

A) Western blotting depicting STING-pSer365 levels in H3.3-G34R and H3.3-Wt human pHGG cells at different times post IR (3 Gy). **B)** Quantification of Western blot results represented in A). **C)** Release of interferon-beta (IFN-β) in H3.3-G34R and H3.3-Wt mouse cells at different times post IR (3 Gy), and inhibition of IFN-β release by STING inhibitors GSK690693 and H151. (*$p<0.05$, **$p<0.01$, ***$p<0.005$, ****$p<0.001$; unpaired t test) **D)** Levels of soluble damage-associated molecular pattern (DAMP) ATP in H3.3-G34R and H3.3-Wt mouse cells in response to (3 Gy), and inhibition of cGAS-STING pathway with the inhibitors GSK690693, H151, JSH23 and PDTC. (*$p<0.05$, **$p<0.01$, ***$p<0.005$, ****$p<0.001$; unpaired t test. **E)** Levels of soluble damage-associated molecular pattern (DAMP) HMGB1 in H3.3-G34R and H3.3-Wt mouse cells in response to (3 Gy), and inhibition of cGAS-STING pathway with: GSK690693 STING-dependent IRF3 activation inhibitor; H151, STING inhibitor; JSH23, NF-κB activation inhibitor; and PDTC, NF-κB inhibitor. *(*$p<0.05$, **$p<0.01$, ***$p<0.005$, ****$p<0.001$; unpaired t test. Data represent mean ± SD of three experimental replicates).*

Supplementary Figure 26

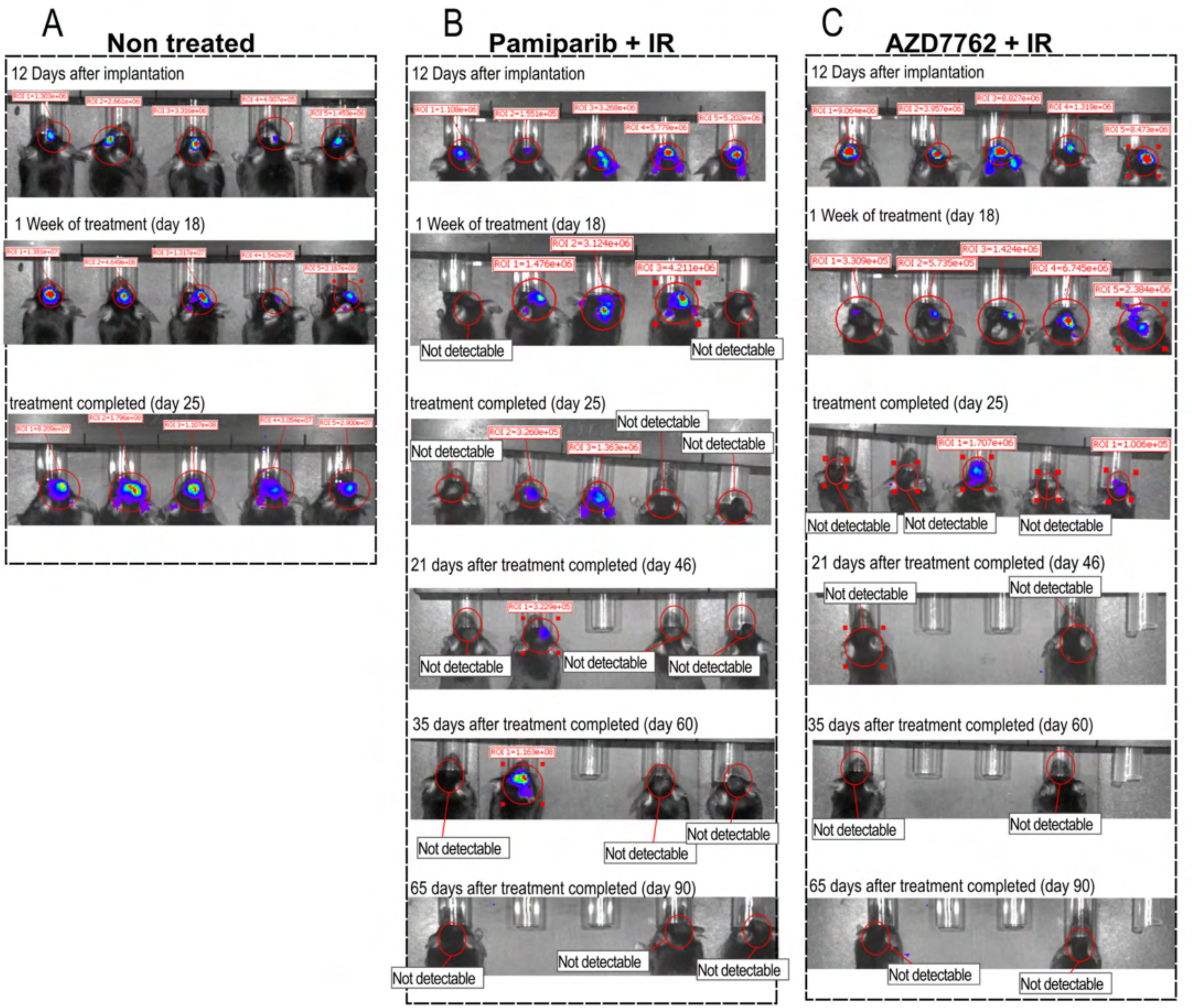

Figure S26

A) Bioluminescence (BLI) monitoring of the progression of H3.3-G34R pHGG in non-treated mice, and mice treated with pamiparib (PARP inhibitor) + RT or AZD7762 (cell cycle checkpoint inhibitor) + RT.

Supplementary Figure 27

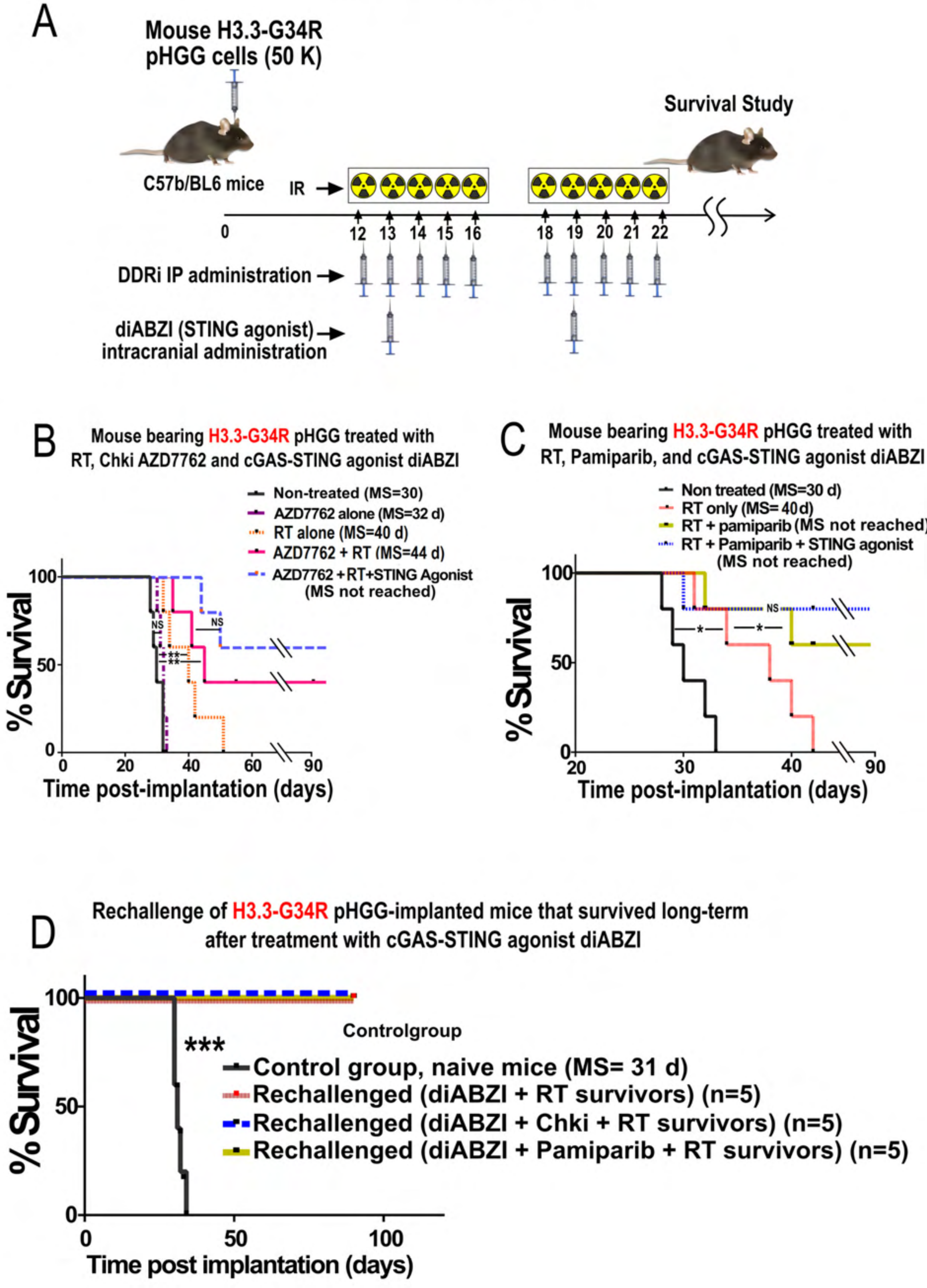

Figure S27

A) Illustration depicting the time frame of the combined treatment with DDRi, RT and the STING agonist diABZI. **B)** Survival of H3.3-G34R-bearing mice treated with RT alone or in combination with the cell cycle checkpoint inhibitor AZD7762 and with the STING agonist diABZI. **C)** Survival of H3.3-G34R-bearing mice treated with RT alone or in combination with the PARP inhibitor pamiparib and with the STING agonist diABZI. *Please note that the non-treated and RT experimental groups shown in B) and C) were included in the same experiment as shown in Figure 8, H.* **D)** Kaplan-Meier survival plot of H3.3-G34R-bearing mice that survived due to the radiotherapy (RT) + DNA damage response inhibitor (DDRi) therapies and rechallenged with H3.3-G34R pHGG cells, compared to naïve mice implanted with the same cells (Control group). *(N=5 mice/group; data were analyzed using the log-rank (Mantel-Cox) test.)*

Supplementary Figure 28

A

Model	H3.3-Wt		H3.3-G34R	
	Pamiparib IC_{50} (μM)	Veliparib IC_{50} (μM)	Pamiparib IC_{50} (μM)	Veliparib IC_{50} (μM)
Mouse cells	60.1	303.4	22.5	121.6
Human genetically engineered cells	87.5	175.3	31.1	86.23
Human endogenous-mutant cells	82	181	15.4	109.6

B

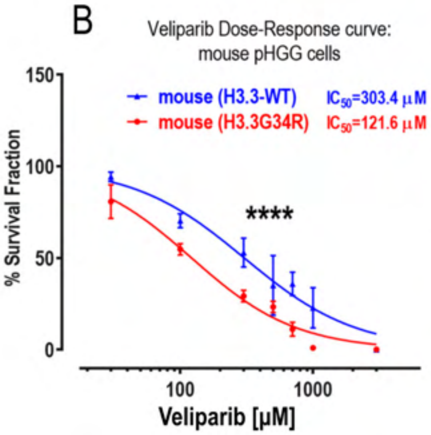

C

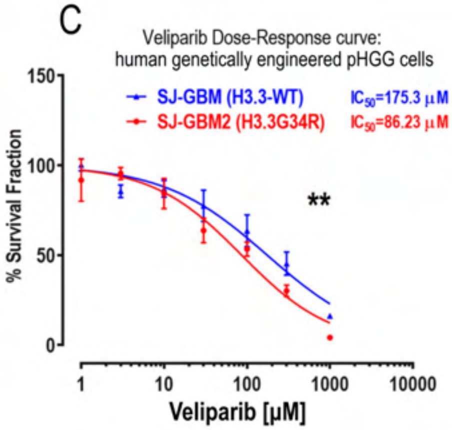

D

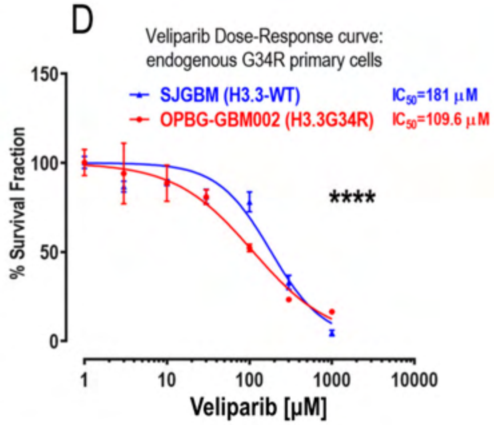

E

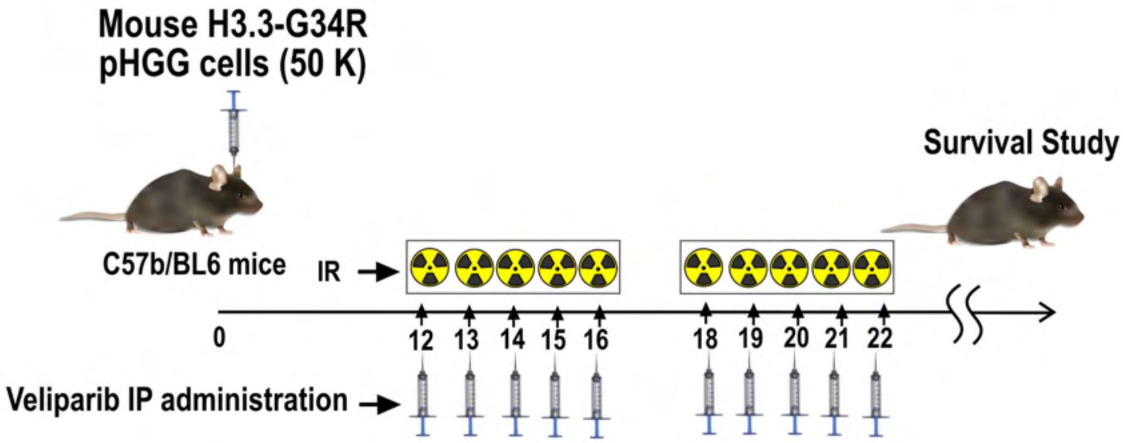

F

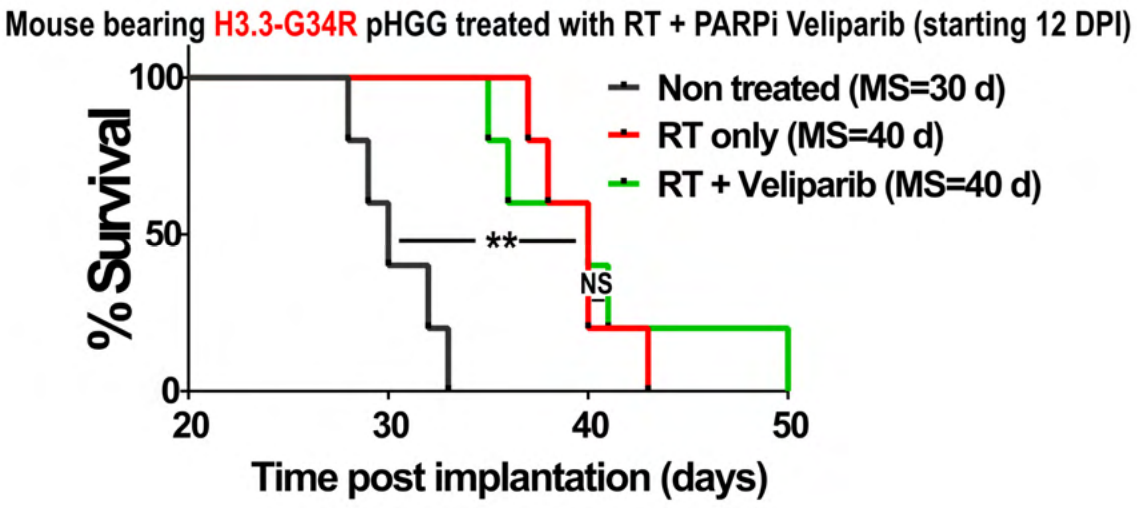

A. IC_{50} for Pamiparib and veliparib in different models. **B-D**. Dose-response curves of H3.3-G34R and H3.3-Wt mouse **(B),** genetically engineered human pHGG cells **(C)**, and patient-derived endogenous G34R-mutant and H3.3-Wt cells **(D)** in response to the PARP inhibitor veliparib. **E)** Illustration depicting the time frame of the combined treatment with veliparib and RT. **F)** Survival of H3.3-G34R-pHGG bearing mice treated with RT alone or in combination with the PARP inhibitor veliparib. (*N=5 mice/group; data were analyzed using the log-rank (Mantel-Cox) test.)*

Cell used in the study

	Genetic markers	Source	Availability
Mouse primary cells			
Mouse H3.3-Wt pHGG cells	P53 kd, ATRX kd, NRAS-G12V	This study	Upon request to MGC
Mouse H3.3-G34R pHGG cells	P53 kd, ATRX kd, NRAS-G12V, H3.3-G34R	This study	Upon request to MGC
Mouse CDKN2a -/-, PDGFRαD842V H3.3-Wt pHGG cells	CDKN2a -/-, PDGFRαD842V, P53 kd, ATRX kd	This study	Upon request to MGC
Mouse CDKN2a -/-, PDGFRαD842V H3.3-G34R pHGG cells	CDKN2a -/-, PDGFRαD842V, P53 kd, ATRX kd	This study	Upon request to MGC
Human primary cells			
SJ-GBM2	P53 lof mut, ATRX lof mut.		Children's Oncology Group (COG) Repository, Health Science Center, Texas Tech University
SJ-GBM2-H3.3-Wt (Human H3.3-Wt pHGG cells)	P53 lof mut, ATRX lof mut, H3.3-Wt ectopic expression	This study	Upon request to MGC
SJ-GBM2-H3.3G-34R	P53 lof mut, ATRX lof mut, H3.3-G34R ectopic expression	This study	Upon request to MGC
OPBG-GBM-001	H3.3-G34R	MV, Department of Onco-Haematology, Gene and Cell Therapy, Bambino Gesù Children's Hospital-IRCCS, Rome, Italy	MV
HSJD-GBM-002	H3.3-G34R	AMC, Institut de Recerca Sant Joan de Deu, Barcelona, 08950, Spain	AMC

Description of supplementary materials:

Table S1: Normalized counts of RNA-seq analysis comparing mouse H3.3-G34R vs H3.3-Wt mouse pHGG cells.

Table S2: Full Differential expression analysis results from mouse H3.3-G34R vs H3.3-Wt mouse pHGG cells RNA-seq.

Table S3: Full Differential expression analysis results from human H3.3-G34R vs H3.3-Wt mouse pHGG cells RNA-seq.

Table S4: List of H3.3-G34R/V and H3.3-Wt hemispherical pHGG patients' samples selected from PedcBioPortal, to perform RNA-seq, mutation count and copy number alteration analyses.

Table S5: Analysis of Differential expression of DNA repair GO genes comparing H3.3-G34R/V versus H3.3-Wt hemispherical pHGG patients' samples selected from PedcBioPortal.

Table S6: List of primers used in the study.

Table S7: List of reagents used in the study.

Table S8: List of antibodies used in the study.

Table S9: Full statistical analysis of Tumor initiating cell frequency presented in Figure, 4 G-H. (*$p<0.05$, **$p<0.01$, ***$p<0.005$, ****$p<0.001$; analysis of median survival from Kaplan–Meier model).pl

Table S10: Full differential expression results of DNA damage response/Cell cycle phospho-array comparing human H3.3-G34R vs H3.3-wt pHGG cells

Table S11: Full differential expression results of DNA damage response/Cell cycle phospho-array comparing mouse H3.3-G34R vs H3.3-wt pHGG cells

Table 4. List of H3.3-G34R and H3.3-WT patients

H3.3-G34R/V Patients

Study	Sample
phgg_cbttc	7316-1099-T-353730
pnet_cbttc	7316-1105-T-353821
pnet_cbttc	7316-158-T
phgg_cbttc	7316-2561-T-460404
phgg_herby	HERBY_005
phgg_herby	HERBY_011
phgg_herby	HERBY_020
phgg_herby	HERBY_021
phgg_herby	HERBY_026
phgg_herby	HERBY_036
phgg_herby	HERBY_055
phgg_herby	HERBY_111
lgg_tcga	TCGA-HT-7469-01
phgg_jones_meta_2017	pHGG_META_0158
phgg_jones_meta_2017	pHGG_META_0179
phgg_jones_meta_2017	pHGG_META_0182
phgg_jones_meta_2017	pHGG_META_0183
phgg_jones_meta_2017	pHGG_META_0203
phgg_jones_meta_2017	pHGG_META_0399
phgg_jones_meta_2017	pHGG_META_0434
phgg_jones_meta_2017	pHGG_META_0437
phgg_jones_meta_2017	pHGG_META_0448
phgg_jones_meta_2017	pHGG_META_0483
phgg_jones_meta_2017	pHGG_META_0522
phgg_jones_meta_2017	pHGG_META_0539
phgg_jones_meta_2017	pHGG_META_0541
phgg_jones_meta_2017	pHGG_META_0549
phgg_jones_meta_2017	pHGG_META_0576
phgg_jones_meta_2017	pHGG_META_0590
phgg_jones_meta_2017	pHGG_META_0829
phgg_jones_meta_2017	pHGG_META_1019
phgg_jones_meta_2017	pHGG_META_1020
phgg_jones_meta_2017	pHGG_META_1023

Wt patients (Hemispheric, ATRXmut, P53mut)

Study	Sample
phgg_cbttc	PT_3CHB9PK5
phgg_cbttc	PT_2WVW55DA
phgg_cbttc	PT_2WVW55DA
phgg_herby	HERBY_013
phgg_herby	HERBY_014
phgg_herby	HERBY_024
phgg_herby	HERBY_039
phgg_herby	HERBY_102
lgg_tcga	TCGA-DU-5852
phgg_jones_meta_2017	pHGG_META_0181
phgg_jones_meta_2017	pHGG_META_0455
phgg_jones_meta_2017	pHGG_META_1001

Table 9. Statistical analysis of tumor initiating cell experiment

Tumor initiating frequency: statistical analysis of median survival

Mouse-H3.3G34R								
Cells implanted	100 K	30 K	10 K	3 K	1 K	300	100	30
100 K		**	***	****	****	****	****	****
30 K	**		ns	ns	**	**	**	**
10 K	***	ns		ns	ns	ns	*	*
3 K	****	ns	ns		ns	ns	*	*
1 K	****	**	ns	ns		ns	*	*
300	****	**	ns	ns	ns		ns	ns
100	****	**	*	*	*	ns		ns
30	****	**	*	*	*	ns	ns	

Mouse-H3.3-Wt								
Cells implanted	100 K	30 K	10 K	3 K	1 K	300	100	30
100 K		**	****	****	****	****	****	****
30 K	**		****	****	****	****	****	****
10 K	****	****		****	****	****	****	****
3 K	****	****	****		**	**	**	**
1 K	****	****	****	**		*	**	**
300	****	****	****	**	*		ns	ns
100	****	****	****	**	**	ns		ns
30	****	****	****	**	**	ns	ns	
